# Impact of toothpaste use on the subgingival microbiome: a pilot randomized clinical trial

**DOI:** 10.1186/s12903-025-06159-z

**Published:** 2025-05-30

**Authors:** Margarita Iniesta, Viviane Vasconcelos, Florencia Laciar, Paula Matesanz, Mariano Sanz, David Herrera

**Affiliations:** 1 ETEP (Etiology and Therapy of Periodontal and Peri-Implant Diseases) Research Group, School of Dentistry, Complutense University of Madrid, Plaza Ramón y Cajal S/N (Ciudad Universitaria), 28040 Madrid, Spain; 2 Present Address: Department of Dental Clinic Specialties, School of Dentistry, Section of Graduate Periodontology, Complutense University of Madrid, Madrid, Spain

**Keywords:** Subgingival microbiome, Gingivitis, Toothpaste, Cetylpyridinium chloride, Cymenol

## Abstract

**Background:**

The subgingival microbiome plays a key role in the gingivitis development, but the impact of toothbrushing with toothpaste on the subgingival microbial composition is not well understood. Therefore, this study aimed to evaluate the microbiological safety and subgingival impact of a toothpaste containing CPC and cymenol, compared to a fluoride-based toothpaste, and assessed overall subgingival microbiome changes after 6 weeks of routine toothbrushing in patients with gingival inflammation.

**Methods:**

A 6-week randomized clinical trial was conducted in patients with gingival inflammation allocated to the use of either a toothpaste with cetylpyridinium chloride and cymenol or a toothpaste with sodium monofluorophosphate. Subgingival samples were collected at baseline and after 6 weeks and processed using high-throughput sequencing technology (Miseq®). Diversity metrics were calculated and the microbiome composition was analyzed using PERMANOVA, ANOSIM and PERMDISP.

**Results:**

A total of 116 samples from 60 patients were analyzed. No significant changes in diversity were observed in either group after 6 weeks. Among taxa with > 1% abundance, the toothpaste with cetylpyridinium chloride and cymenol exhibited a higher reduction in *Aggregatibacter* (*p* = 0.023) and a significant decrease in *Fusobacterium nucleatum* (*p* = 0.030), while the toothpaste with sodium monofluorophosphate showed a significant increase in the phylum *Firmicutes* (*p* = 0.033). The relative abundance of *Porphyromonas gingivalis*, *Prevotella intermedia* and *Tannerella forsythia* were not affected by either toothpaste (*p* > 0.05).

**Conclusions:**

The daily use of a CPC/cymenol toothpaste was microbiologically safe, with no negative effects on the composition of the subgingival microbiome in patients with gingival inflammation, when compared to a fluoride-based toothpaste. The overall composition of the subgingival microbiome was not significantly affected by the daily use of either toothpaste after 6 weeks. In both groups, the observed changes affected mainly the low-abundance taxa.

**Trial registration:**

Registration Number: ISRCTN17497809; Registration Date: 12/07/2023 (ISRCTN.org).

**Supplementary Information:**

The online version contains supplementary material available at 10.1186/s12903-025-06159-z.

## Background

The etiopathogenesis of periodontal diseases is currently understood as a disturbance in the symbiotic relationship between the oral microbiome and the host immune response [1]. Changes in either the quality and/or quantity of the biofilm, or in the host’s immune response, may lead to dysbiosis and the development of the inflammatory conditions defined as periodontal diseases [2, 3]. An increase in biofilm biomass will cause gingivitis, characterized by marginal gingival inflammation, which may be reversed by biofilm control [4], or become chronic and, if unresolved, it may progress to periodontitis [5].

The microbiome associated with gingivitis has been associated with an abundant and heterogeneous microbiological composition, since in some gingivitis patients their microbiome is indistinguishable from a health-associated microbiome, while in others, it shows profiles similar to what has been described in periodontitis [6]. These differences may reflect different degrees of gingival inflammation [7, 8], suggesting that gingivitis could act as a transient clinical state towards periodontitis in certain individuals. This hypothesis is supported by findings of polymicrobial synergy within the dysbiosis model, advocating that periodontitis is not driven by individual pathogens, but by a collaborative polymicrobial community in dysbiosis, which disrupts host homeostasis, probably through an unresolved chronic inflammation, eventually leading to tissue destruction [9, 10].

Within this context, one of the most efficient methods to prevent periodontitis is the treatment of biofilm-induced gingivitis by means of effective oral hygiene practices [11], typically achieved through mechanical methods such as toothbrushing, flossing, and interdental brushing. Mechanical approaches can be supported in some instances with the use of antiseptic formulations in toothpastes or mouthrinses [12]. However, there is limited knowledge on how oral hygiene practices influence the subgingival microbiome: it may be worth testing the impact of toothbrushing with different toothpastes, to explore the differential impact of microbial ecology, together with the evaluation of clinical efficacy and microbiological safety.

Toothpastes may include antiseptic agents in their formulation, such as cetylpyridinium chloride (CPC), which is frequently included in oral hygiene products. It has been suggested that its antimicrobial activity can be enhanced by combining it with other antiseptics, such as essential oils [13] or biguanides [14]. Cymenol, a phenolic compound derived from thymol, exhibits direct antimicrobial effects [15]. In vivo studies have shown antimicrobial efficacy of mouth rinses based on this essential oil in combination with CPC [16] and zinc salts [17].

However, to date, the microbiological impact of a toothpaste containing CPC and cymenol has not been evaluated. Therefore, the objective of the present study was to investigate the microbiological safety and ecological effects of daily toothbrushing with a new CPC/cymenol toothpaste on the subgingival microbiome in patients with gingival inflammation, comparing it with those occurring after the use of a fluoride-containing toothpaste. In addition, overall changes in the subgingival microbiome after routine toothbrushing over a 6-week period were assessed.

## Methods

### Study design

This randomized controlled clinical trial was designed as a single-center, double-blind, pilot study and is reported according to CONSORT guidelines. The study was conducted at the Postgraduate Periodontal Clinic at the School of Dentistry in the Complutense University of Madrid, Spain, following the principles of the Declaration of Helsinki, from February to December 2022. The protocol was first registered in the *Registro Investigación* at the School of Dentistry (registration #49–290421 [﻿Conformidad Centro 49–290421]) and subsequently in the ISRCTN registry (Springer Nature registration #ISRCTN17497809 (https://doi.org/10.1186/ISRCTN17497809). The clinical results of the study have been published in a separate paper [18].

The study was independently reviewed and approved by the Ethics Committee of *CEIC Hospital Clínico San Carlos*, Madrid, Spain (registration number 21/262-EC_X). All participants provided written informed consent before enrollment, acknowledging their understanding of the study procedures, risks, and benefits.

### Subjects

Consecutive subjects diagnosed with biofilm-induced gingivitis or gingival inflammation in previously treated periodontitis patients [4], were recruited after fulfilment of predefined inclusion/exclusion criteria.

#### Inclusion criteria

- over 18 years of age;
- systemically healthy;
- presence of at least three evaluable teeth in each quadrant;
- moderate gingival inflammation, defined as ≥ 40% bleeding on marginal probing (BOMP) [19];
- Turesky plaque index ≥ 1.5 [20];
- no probing depths (PD) ≥ 5 mm;
- no fixed orthodontic treatments or removable prostheses;
- tooth brushing habits of at least twice a day.

#### Exclusion criteria

- untreated or uncontrolled periodontitis;
- regular antiseptic mouth rinse users;
- antibiotic intake in the month prior to screening;
- pregnant or in lactation women;
- presence of chronic diseases or medication that may influence gingival inflammation;
- systemic conditions requiring antibiotic coverage.

After the clinical examination, subjects were randomly assigned to one of the two toothpastes: a toothpaste, with 0.05% CPC and 0.09% cymenol (cym) as main active ingredients (*Bexident® Encías Uso Diario* toothpaste, ISDIN, Barcelona, Spain) (CPC/cym); and a toothpaste sodium monofluorophosphate (MFP) (Colgate Protection Caries toothpaste, Colgate-Palmolive España S.A., Madrid, Spain), which has demonstrated a well-established safety profile [21]. Participants were provided with standardized verbal and written instructions requesting them to brush their teeth three times a day for two minutes, after each of the three main meals (breakfast, lunch, and dinner). Subjects were also provided with a manual toothbrush (UltraThin ProGumCare®, OralB, Madrid, Spain), together with the assigned toothpaste. No further interventions were carried out during the study period.

Subjects were identified through a unique trial number. Participants were randomly allocated to one of the two toothpaste groups. Randomization was performed using random numbers from a computer-generated list with Microsoft Excel® software, in blocks of six patients by a researcher not involved in the clinical evaluations. All subjects were blinded to their product assignment and received their product kits with a unique identification code per patient (code A or B) depending on whether they were assigned the CPC/cym or MFP toothpaste.

### Microbiological sampling

Microbiological subgingival samples were collected from the same sites, preferentially at the mesial site of first molars (or alternatively, of second molars or second premolars) depending on the presence of bleeding during the baseline evaluation, in both upper and lower jaws at two time points, baseline and 6 weeks. Two consecutive sterile paper points per site (#30 size) (Maillefer; Ballaigues, Switzerland) were inserted and held within the periodontal pocket for 10 s. Samples were then pooled resulting in unique sample with four paper points per patient and visit. The pooled samples were transferred to a cryogenic vial with DNA/RNA Shield™ reagent and subsequently sent by courier to Microomics® (IIB Sant Pau, Barcelona, Spain) for further processing.

### Microbiological processing

The composition and structure of the sampled microbial communities were assessed by Next Generation Sequencing (NGS) using amplification and sequencing of the V3-V4 variable regions of the *16S rRNA* gene. The Illumina® Miseq® sequencing 300 × 2 approach was used. Amplification was performed after 25 polymerase chain reaction (PCR) cycles. A negative control of the DNA extraction was included as well as a positive Mock Community control to ensure quality control.

Raw demultiplexed forward and reverse reads were processed using the methods and pipelines implemented in QIIME2 version 2019.4 with default parameters [22]. DADA2 was used for quality filtering, which avoids errors, and was processed with the fusion of paired-end reads and amplicon sequence variant (ASV, i.e., phylotypes) through the denoise-paired qiime dada2 method. ASVs were aligned using the mafft method in qiime alignment [23]. The alignment was used to create a tree and calculate the phylogenetic relationships among ASVs using the qiime phylogeny fasttree method (http://www.microbesonline.org/fasttree/, RRID:SCR_015501).

Taxonomic assignment of phylotypes was performed using a Bayesian Classifier trained with the Silva database version 138 (99% ASVs full-length sequences) (http://www.arb-silva.de, RRID:SCR_006423).

### Data analysis

#### Sample size calculation

This study was primarily designed as a pilot study to assess safety and patient tolerability of the CPC/cym toothpaste. A convenience sample of 60 patients (30 patients per group) was selected [18].

#### Statistical analysis

Quantitative data were expressed as means and standard deviations (SD) and analyzed using the Mann–Whitney U and Wilcoxon tests. Categorical data were expressed as percentages. Data on categorical variables were compared using the chi-square or Fisher’s exact tests. This part of the analysis was performed using IBM® SPSS® STATISTICS 27.0 (https://www.ibm.com/products/spss-statistics, RRID:SCR_016479).

Alpha diversity comparisons were performed using a linear model, and richness (number of observed ASVs) and evenness (Pielou’s Evenness index) were calculated. Beta diversity was measured using phylogenetic metrics (weighted and unweighted Unifrac distances) [24] and a compositional beta diversity metric, such as the Jaccard and Bray Curtis distances. Beta diversity distance matrices were used to calculate principal coordinate analysis (PCoA) and to create ordination plots using the R software package version 4.2.0 (http://www.r-project.org/, RRID:SCR_001905). The significance of groups present in the community structure was tested using PERMANOVA and ANOSIM tests. The PERMDISP test was used to identify location versus dispersion effects [25].

Differential relative abundance of taxa was tested using a linear model based on the negative binomial distribution. *P*-values were adjusted using the false discovery rate. The significance threshold was set at 0.05.

The packages BiodiveristyR version 2.14–1 [26], PMCMRplus version 1.9.4 [27], RVAideMemoire version 0.9–8 (http://CRAN.R-project.org/package=RVAideMemoire, RRID:SCR_015657), and vegan version 2.5–6 (http://cran.r-project.org/web/packages/vegan/index.html, RRID:SCR_011950) were used for the different statistical analyses performed.

Sequencing, bioinformatics processing, and statistical analysis of the microbiome were performed by Microomics® Systems S.L. (Barcelona, Spain).

## Results

### Subjects

A total of 116 samples from 60 patients were analyzed. In four subjects of the MFP group, subgingival samples could not be collected at 6 weeks for different logistic reasons. The CONSORT flow diagram was previously reported in detail (Vasconcelos et al., 2024). No significant differences were observed in terms of age, gender, and smoking habit between the two treatment groups at baseline (Table 1). No significant differences in plaque index or bleeding on probing, at the sampled sites, were observed between groups at baseline or at 6 weeks. However, significant intragroup differences were observed between baseline and 6 weeks at the site level, in both groups (Table 2).

**Table 1:** Demographic characteristics of patients providing subgingival samples for microbiological analysis

		**CPC/cym**	**MFP**	**All**	***p***
	*n*	30	30	60	
**Age**	Mean (SD)	38.83 (17.60)	42.13 (18.07)	40.48 (17.76)	
	Median (IQR)	31.00 (0.31)	40.00 (0.36)	33.00 (0.35)	0.515
**Gender**	Male, n (%)	12 (40.0)	12 (40.0)	24 (40.0)	1.000
	Female, n (%)	18 (60.0)	18 (60.0)	36 (60.0)	
**Smoking**	No, n (%)	27 (90.0)	28 (93.3)	55 (91.7)	1.000
	Yes, n (%)	3 (10.0)	2 (6.7)	5 (8.3)	

Treatment groups: CPC/cym, toothpaste with cetylpyridinium chloride (CPC) and cymenol (cym), as main active ingredients; MFP, toothpaste with sodium monofluorophosphate (MFP)

*SD *Standard deviation, *IQR *Interquartile range, *n *Sample size, *% *Percentage of patients

**Table 2:** Plaque index and bleeding on probing at the selected subgingival sampled sites, analyzed per protocol

**Variable**		**CPC/cym (*n* = 30)**		**MFP (***n*** = 26)**		***p***
		Mean (SD)	Median (IQR)	Mean (SD)	Median (IQR)	
**Plaque index**	Baseline	3.08 (0.55)	3.00 (0.25)	3.03 (0.44)	3.00 (0.50)	0.786
	6w	2.33 (0.99)	2.5 (1.00)**	2.63 (0.78)	3.00 (0.50)*	0.197
	Change BL-6w	0.75 (0.99)	0.50 (1.00)	0.40 (0.78)	0.25 (0.50)	0.143
**Bleeding on probing**	Baseline	0.93 (0.21)	1.00 (0.00)	0.96 (0.13)	1.00 (0.00)	0.740
	6w	0.41 (0.41)	0.50 (1.00)**	0.46 (0.31)	0.50 (0.13)**	0.559
	Change BL-6w	0.51 (0.42)	0.50 (1.00)	0.50 (0.34)	0.50 (0.25)	0.861

Treatment groups: CPC/cym, toothpaste with cetylpyridinium chloride (CPC) and cymenol (cym), as main active ingredients; MFP, toothpaste with sodium monofluorophosphate (MFP)

*n *sample size, *SD *Standard deviation, *IQR *Interquartile range, *BL *Baseline, *6w *6 weeks

^*^Statistically significant intragroup difference between baseline and 6 weeks (*p* < 0.05)

^**^Statistically significant intragroup difference between baseline and 6 weeks (*p* < 0.001)

### Summary of sequencing data

After quality control, a final number of 7,791 phylotypes were detected. Samples were subsampled up to 3,240 reads to equalize sample size and facilitate quantitative comparisons. A total of 115 samples were examined for diversity analysis, as the sample from patient #29 at baseline had to be removed due to insufficient depth subsampling. Rarefaction curves demonstrated good sequencing depth and appropriate subsampling size (see Supplementary Fig. 1).

### Pooled effect of toothbrushing on the subgingival microbiome

More than 98% of the relative abundance was attributable to eight major phyla, including *Bacteroidota* (27.91%), *Fusobacteriota* (20.37%), *Firmicutes* (19.37%), *Proteobacteria* (10.19%), *Actinobacteriota* (9.40%), *Spirochaetota* (5.46%), *Campilobacterota* (3.24%), and *Patescibacteria* (2.77%). The other phyla present were *Synergistota* (0.73%), *Desulfobacterota* (0.36%), *Chloroflexota* (0.12%), and *Verrucomicrobiota* (0.05%). At 6 weeks, the microbiome was still dominated by *Bacteroidota* (25.72%), followed by *Firmicutes* (22.13%) and *Fusobacteriota* (19.70%) (see Supplementary Table 1).

At the genus level, *Fusobacterium* (16.47%) was the most abundant genus, followed by *Prevotella* (10.07%), *Capnocytophaga* (6.08%), and *Treponema* (5.46%). Other genera with a relative abundance < 5% were *Porphyromonas* (4.32%), *Leptotrichia* (3.68%), *Streptococcus* (3.30%), *Campylobacter* (3.23%), *Veillonella* (3.07%), *Actinomyces* (2.90%), *Rothia* (2.78%), *Corynebacterium* (2.44%), *Neisseria* (2.12%), F0058 (1.94%), *Haemophilus* (1.82%), *Alloprevotella* (1.81%), *Aggregatibacter* (1.77%), *Selenomonas* (1.60%), *Tannerella* (1.59%), *Cardiobacterium* (1.47%), *Enterococcus* (1.38%), *Saccharimonadaceae* (1.31%), and *Alkalibacterium* (1.27%). At the end of the study, the top seven genera in relative abundance remained the most prevalent, with no major changes in their proportions. The prevalence data of all genera identified are shown in Supplementary Table 1.

At the species level, the species exhibiting the highest prevalence in patients with gingival inflammation were *Fusobacterium nucleatum* (7.51%) and other *Fusobacterium* spp. (8.88%). Other species were *Veillonella* spp. (2.81%), *Rothia* spp. (2.26%), *Campylobacter gracilis* (2.19%), *Corynebacterium matruchotii* (2.07%), F0058 uncultured bacterium (1.94%), *Actinomyces* spp. (1.91%), *Streptococcus* spp. (1.85%), *Treponema medium* (1.45%), *Porphyromonas gingivalis* (1.38%), *Porphyromonas endodontalis* (1.35%), *Prevotella nigrescens* (1.34%), *Haemophilus parainfluenzae* (1.32%), *Alkalibacterium* spp. (1.27%), *Capnocytophaga gingivalis* (1.25%), *Capnocytophaga ochracea* (1.23%), *Prevotella intermedia* (1.23%), *Alloprevotella tannerae* (1.22%), *Enterococcus* spp. (1.14%), *Treponema socranskii* (1.08%), *Treponema denticola* (1.05%), *Prevotella denticola* (1.04%), *Cardiobacterium* uncultured bacterium (1.04%), and *Tannerella forsythia* (1.02%). After 6 weeks, species of *Fusobacterium* continued to dominate the microbiome (15.11% of the total), followed by *C. matruchotii* (2.81%), *Veillonella* spp. (2.55%), and *Streptococcus* spp. (2.43%). The prevalence data of all species identified are shown in Supplementary Table 1.

### Alpha and beta diversity of the subgingival microbiome by visit and toothpaste group

**Fig.1:**
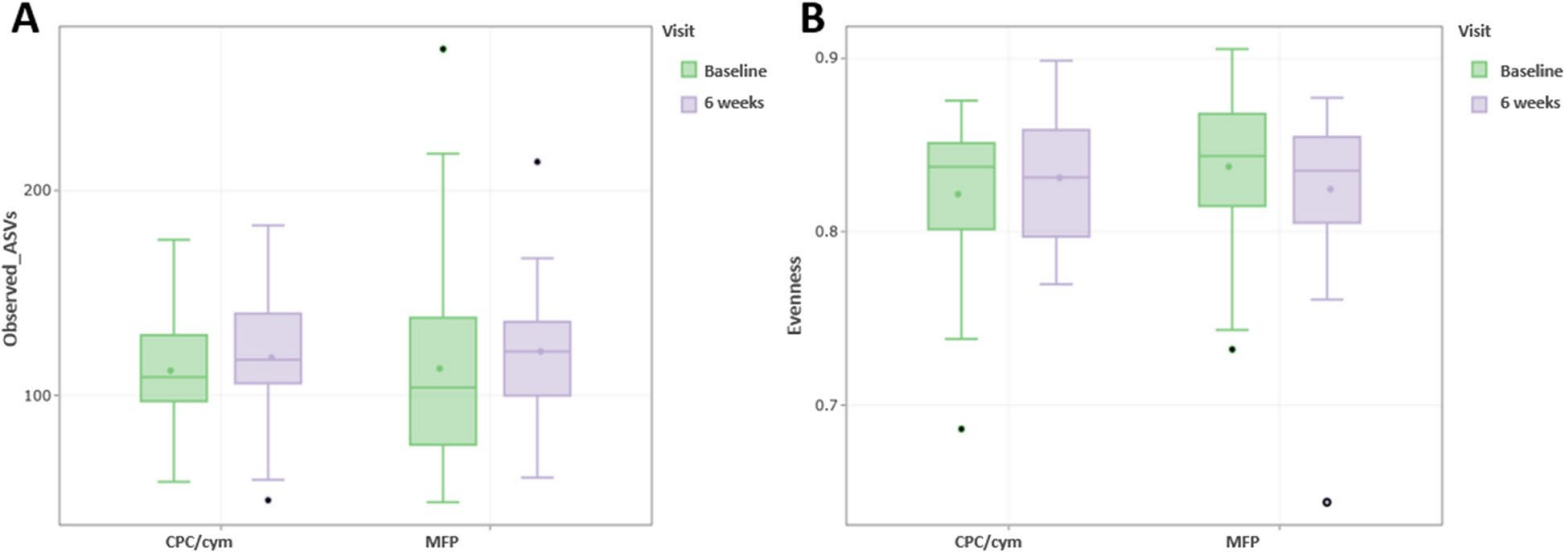
Comparison of the subgingival communities between toothpastes. The analysis did not show any difference in α-diversity metrics at the phylotype level. Richness (observed amplicon sequence variants, ASVs) (**A**) and evenness (Pielou’s Evenness index) (**B**). Treatment groups: CPC/cym, toothpaste with cetylpyridinium chloride (CPC) and cymenol (cym), as main active ingredients; MFP, toothpaste with sodium monofluorophosphate (MFP)

At baseline, samples from the CPC/cym and MFP groups had similar levels of species richness (number of observed ASVs) and evenness (Pielou’s Evenness index) (*p* = 0.907 and *p* = 0.136, respectively). At 6 weeks, the number of observed ASVs increased in both groups, and community evenness decreased in the MFP group, but the differences were not statistically significant (*p* = 0.740 and *p* = 0.585, respectively). The interaction between time and toothpaste showed no statistically significant effect on species richness or evenness (*p* = 0.701 and *p* = 0.084, respectively). No significant intragroup differences between baseline and final visits were also observed in the number of observed ASVs (test group, *p* = 0.424; control group, *p* = 0.195) or Pielou’s Evenness index (test group, *p* = 0.325; control group, *p* = 0.151) (Fig. 1).

**Fig. 2:**
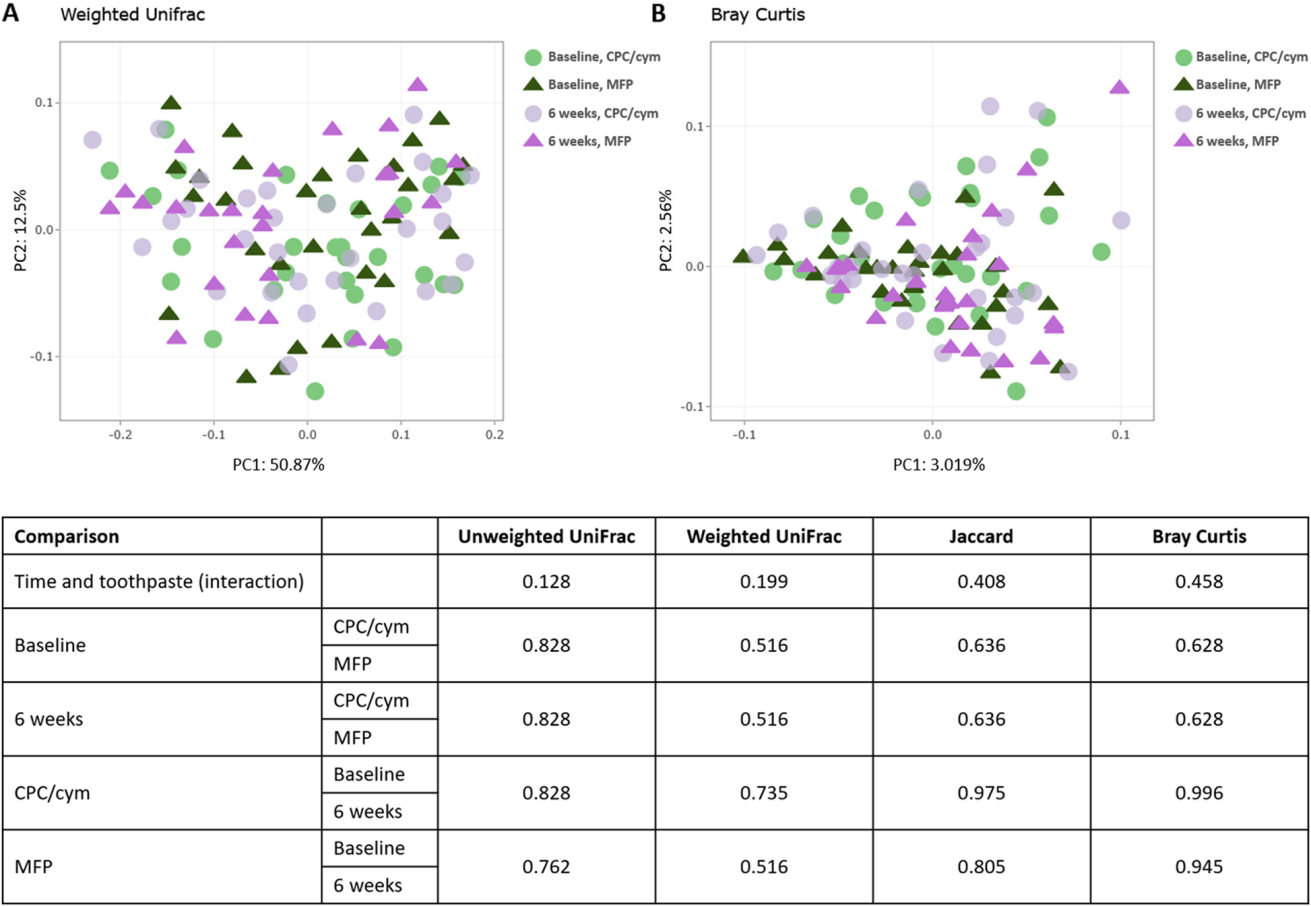
No variation in the subgingival communities was observed between or within groups based on principal coordinate analysis (PCoA) or different metrics such as weighted UniFrac (**A**) and Bray Curtis (**B**) distances. All samples were plotted on the first two principal components (PCs) of the phylotype profile. Treatment groups: CPC/cym, toothpaste with cetylpyridinium chloride (CPC) and cymenol (cym), as main active ingredients; MFP, toothpaste with sodium monofluorophosphate (MFP)

To identify changes in the structure of the biofilm microbiome, following the use of the assigned toothpastes, the 115 samples were grouped into four time points using PCoA, based on the relative abundance of phylotypes (Fig. 2). No changes were observed between the two groups during the study period. In addition, no significant differences in beta diversity metrics were detected between baseline and 6-week visits for either group (*p* < 0.05) (Fig. 2).

### Relative abundance after the toothpaste use

#### Phylum level

**Fig. 3:**
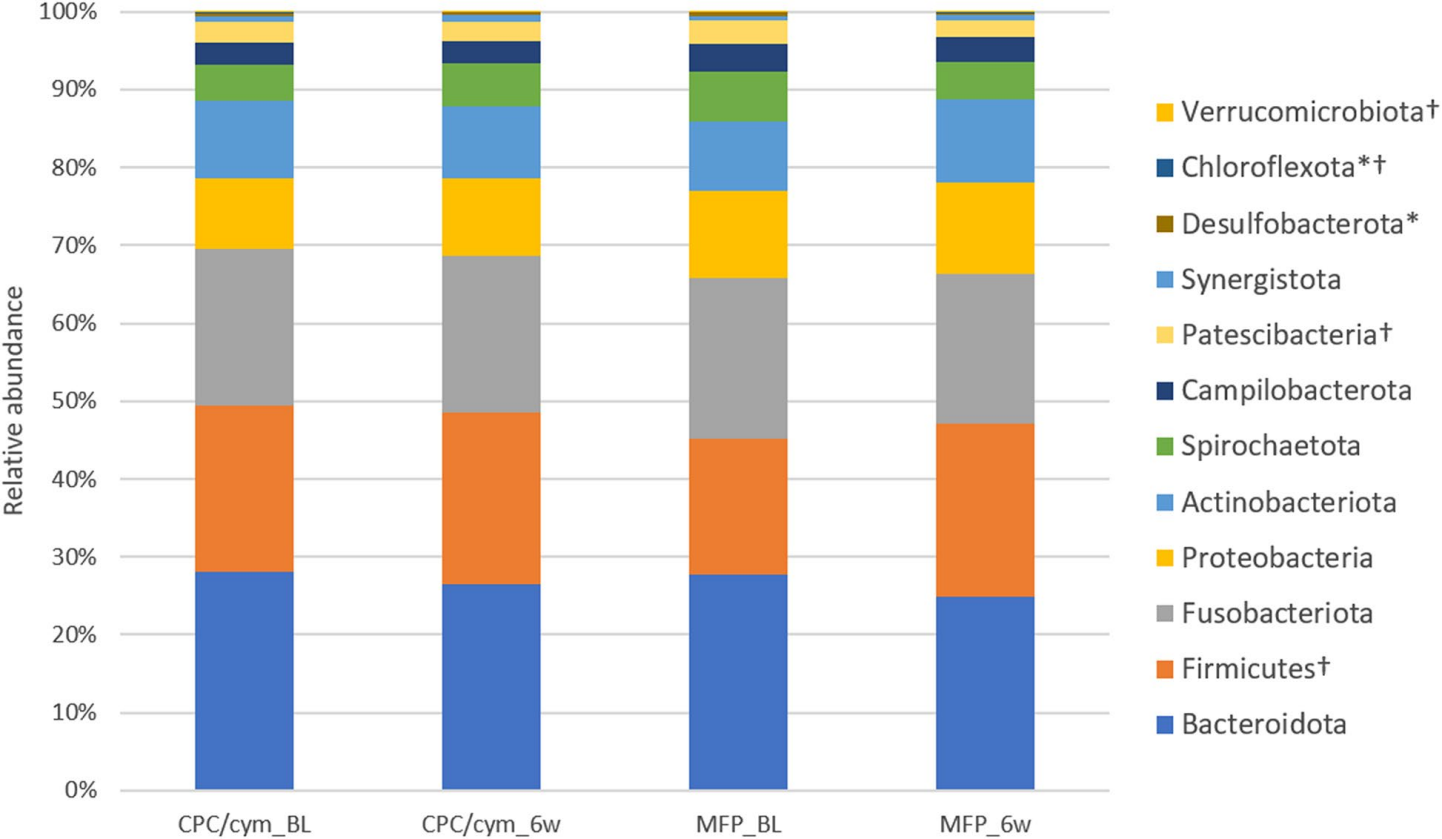
Changes in the relative abundance of the phyla with an abundance > 0.05%. Treatment groups: CPC/cym, toothpaste with cetylpyridinium chloride (CPC) and cymenol (cym), as main active ingredients; MFP, toothpaste with sodium monofluorophosphate (MFP). BL: baseline; 6w: 6 weeks. *: significant intragroup differences in CPC/cym; †: significant intragroup differences in MFP

Overall, no statistically significant differences were observed in the different phyla between groups (intergroup differences), neither at the initial (*p* > 0.05) nor at the final visit (*p* > 0.05). In terms of intragroup differences, after using the CPC/cym toothpaste, a significant decrease was observed in some phyla, such as *Desulfobacterota* and *Chloroflexota*. Following the use of the MFP toothpaste, a statistically significant increase in *Firmicutes* and *Verrucomicrobiota,* and a significant decrease in *Patescibacteria* and *Chloroflexota,* were observed (Fig. 3).

#### Genus level

**Fig. 4:**
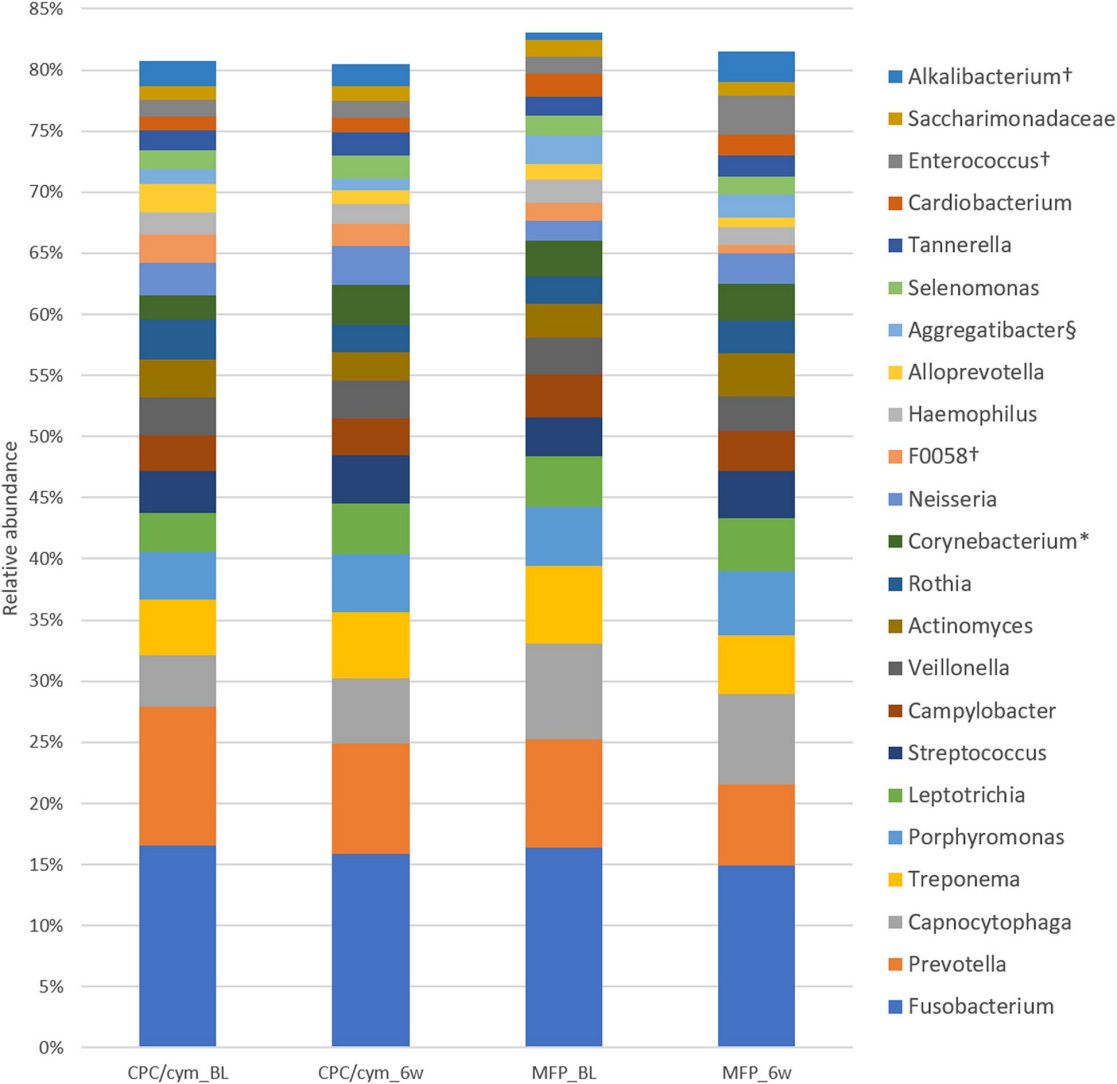
Changes in the relative abundance of the genera with an abundance > 1%. Treatment groups: CPC/cym, toothpaste with cetylpyridinium chloride (CPC) and cymenol (cym), as main active ingredients; MFP, toothpaste with sodium monofluorophosphate (MFP). BL: baseline; 6w: 6 weeks. *: significant intragroup differences in CPC/cym; †: significant intragroup differences in MFP. §: significant intergroup differences at week 6

The relative abundance at the genus level, by toothpaste and visit, is shown in the heat map (see Supplementary Fig. 2); and changes in the genera with a relative abundance > 1% are shown in Fig. 4.

At the 6-week visit, a significantly higher relative abundance was observed in the CPC/cym group, compared with the MFP group in the genera *Granulicatella*, *Mycoplasma*, *Roseburia,* and *Alkalimonas*; and a significant lower abundance in *Lactococcus*, *Staphylococcus*, *Pseudoramibacter*, *Lachnospiraceae* uncultured, *Candidatus* Riegeria, and *Aggregatibacter*. The dynamics of genera that increased or decreased from baseline to 6 weeks for both groups are shown in Supplementary Table 2.

#### Species level

**Fig. 5:**
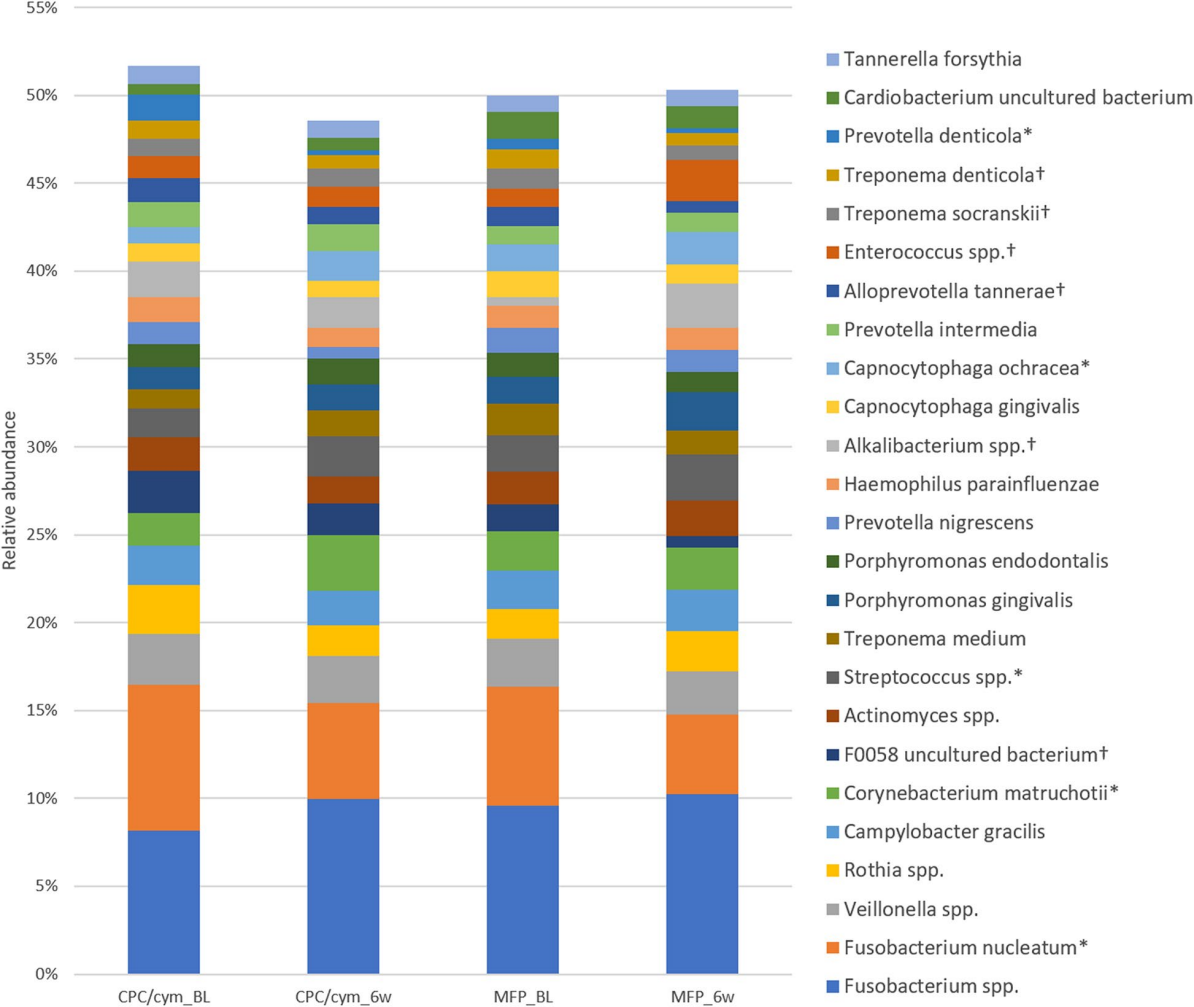
Changes in the relative abundance of the species with an abundance > 1%. Treatment groups: CPC/cym, toothpaste with cetylpyridinium chloride (CPC) and cymenol (cym), as main active ingredients; MFP, toothpaste with sodium monofluorophosphate (MFP). BL: baseline; 6w: 6 weeks. *: significant intragroup differences in CPC/cym; †: significant intragroup differences in MFP

At the final visit, a significant higher relative abundance was observed in the CPC/cym group, compared with the MFP group, in *Prevotella buccae*, *Prevotella* spp., *Granulicatella* spp., *Lachnospiraceae bacterium*, *Schwartzia* spp., and *Saccharimonadaceae* spp.; and a lower abundance in *Capnocytophaga granulosa*, *Capnocytophaga leadbetteri*, uncultured *Capnocytophaga*, uncultured *Bergeyella*, *Campylobacter rectus*, *Staphylococcus* spp., *Pseudoramibacter* spp., *Centipeda* uncultured bacterium, TM7x uncultured *Candidatus*, *Candidatus* Riegeria uncultured bacterium, and *Alishewanella* uncultured *Rheinheimera*. Figure 5 shows the changes in the species with a relative abundance greater than 1%. The changes in species that either increased or decreased from baseline to the 6-week visit for both groups are detailed in Supplementary Table 3.

## Discussion

The present study aimed to evaluate the microbiological safety and ecological effects of daily toothbrushing using a toothpaste containing CPC and cymenol, in comparison with the use of a MFP-based toothpaste, in patients with gingival inflammation. Overall, the subgingival microbiome in these patients showed limited changes during the 6-week of toothbrushing period with the assigned toothpastes, without evidencing a significant impact on the diversity of the subgingival microbiome. In parallel, a previous clinical analysis from the same trial reported statistically significant higher reductions in plaque and bleeding levels with the CPC/cym toothpaste, when compared to the MFP toothpaste [18].

The effects of the two tested toothpastes on the subgingival microbiome diversity were similar, with no significant intergroup or intragroup differences observed. Similarly, a recent study evaluating the effect on the supragingival biofilm of 12 weeks of using a mouth rinse containing CPC plus essential oils, compared with a control containing water with cinnamaldehyde, did not detect differences in alpha diversity [13]. However, significant differences in beta diversity were reported. Differences in the sampled niche (supragingival versus subgingival), delivery format (mouth rinse versus toothpaste), as well as the formulations of the tested products and the interventions [professional mechanical plaque removal (PMPR) versus no PMPR] may explain the differences in the results. In another study evaluating the subgingival microbiome after PMPR and the daily use a toothpaste containing MFP [28], a significant decrease in both alpha and beta diversity was reported. These results may suggest that both CPC/cym and MFP-based toothpastes, without PMPR, may exert a similar moderate impact on the overall subgingival community structure, affecting only a limited number of taxa without significantly altering diversity. These findings are also consistent with the conclusions of a previous comprehensive review on the impact of biofilm control on the microbiome composition associated with gingivitis [29].

At 6 weeks, no statistically significant intergroup differences were found at the phyla level. Among taxa with > 1% of relative abundance, only the CPC/cym toothpaste demonstrated lower levels of the genus *Aggregatibacter* compared to MFP, potentially due to the effects of CPC, as observed in a previous in vitro study [30]. Differences between groups were mainly observed in taxa < 1% relative abundance. In the CPC/cym group, a higher abundance of the genus *Granulicatella* was observed, when compared to MFP, which is associated with periodontal health [6, 31]. In addition, CPC/cym showed a significant lower abundance in certain genera, such as *Pseudoramibacter* and *Lachnospiraceae* uncultured, among others, which are associated with periodontitis [6, 31]. These shifts suggest that daily use of the CPC/cym toothpaste may help modulate the subgingival microbiota by reducing potential pathogenic genera and promoting the abundance of taxa associated with periodontal health.

Regarding intragroup differences, in the MFP toothpaste group there was an increase in phyla with > 1% relative abundance, specifically *Firmicutes* and *Patescibacteria*. Bamashmous et al. (2021) observed a similar increase in *Firmicutes* following PMPR and the use of a MFP-containing toothpaste [28]. Meanwhile, some known periodontal pathogens, such as *P. gingivalis*, *P. intermedia*, and *T. forsythia*, were not affected by either toothpaste. However, species with antimicrobial activity against *P. gingivalis* and *T. denticola,* like *S. gordonii* [32], and against *P. intermedia*, *P. gingivalis*, and *F. nucleatum,* such as *S. salivarius* [33], were increased with both toothpastes. Both groups also showed an increase in various *Actinomyces* species, while CPC/cym specifically showed an increase in *N. bacilliformis*, *V. parvula*, and *Granulicatella* spp., all associated with oral health [34]. Previous studies have also observed increases in the genus *Actinomyces* and several related species with MFP-based toothpastes [28] and CPC-based mouth rinses [13]. *T. denticola* was not affected in the CPC/cym toothpaste, consistent with findings from Kang et al. (2015), which observed that CPC had no effect on this microorganism [35]. Different species within the same genus exhibited distinct responses to the same toothpaste, suggesting species-level variability.

*F. nucleatum*, one of the most abundant species in patients with gingival inflammation and a key player in biofilm formation by bridging early and late colonizers [36], was significantly reduced only in the CPC/cym group. This effect may be due to a synergistic action of the two active components in the CPC/cym toothpaste, as both cymenol [15] and CPC [35] have shown a direct inhibitory effect on *F. nucleatum *in vitro*.* Another possible explanation could be the greater reduction in the plaque index observed in this toothpaste group, as reported in the clinical publication [18].

The present study has different limitations that must be acknowledged. Firstly, the follow-up period was limited to 6 weeks, precluding an evaluation of the long-term effect of the tested toothpastes. Secondly, as a pilot study focused on microbiological safety, the sample size was relatively small. In addition, the absence of standardized toothbrushing instructions may have influenced the microbial outcomes. Finally, the ribosomal RNA database used was not specific to the human oral microbiome. This fact provides both advantages and disadvantages. While the SILVA database includes taxa not present in the Human Oral Microbiome Database (HOMD), such as *Acholeplasma*, *Flexilinea,* and *Serpentinicella*, which were affected by CPC/cym, it can also result in taxa being named with numbers, like F0058 or F0332, which correspond to the genera *Bacteroidales*_[G-2] and *Peptidiphaga*, respectively, in the HOMD.

Future studies should include a larger number of subjects and extend the study period. In addition, it would be useful to evaluate the microbiome at the functional level, as the metabolic activity of certain taxa may not correlate with their presence in the biofilm, and taxa with reduced abundance could have high metabolic activity [37].

## Conclusions

With the limitations of this pilot study, it can be concluded that daily toothbrushing with a CPC plus cymenol-containing toothpaste was microbiologically safe, similar to that of the reference MFP-based toothpaste. These changes occurred mainly in low-abundance taxa, so the microbiome diversity was not significantly affected. Both toothpastes induced compositional changes, yet the subgingival biofilm remained relatively stable over the 6-week period, suggesting that while the subgingival microbiome exhibits interesting dynamics, its overall structure is not easily altered by short-term interventions or regular toothbrushing alone.

## Supplementary Information

Supplementary Material 1.

## Data Availability

Sequencing, bioinformatics processing, and statistical analysis of the microbiome were performed by Microomics Systems S.L. (Barcelona, Spain). The datasets analyzed during the current study are available at Zenodo (10.5281/zenodo.15115140).

## Abbreviations

CPC: Cetylpyridinium chloride
Cym: Cymenol
MFP: Monofluorophosphate
BOMP: Bleeding on marginal probing
PD: Probing depth
PCR: Polymerase chain reaction
SD: Standard deviation
ASV: Amplicon sequence variant
PCoA: Principal coordinate analysis
spp: Species
PMPR: Professional mechanical plaque removal
HOMD: Human Oral Microbiome Database
