# Supplementary material for: Impact of toothpaste use on the subgingival microbiome: a pilot randomized clinical trial
Source: BMC Oral Health. 2025 May 30;25:854. doi: 10.1186/s12903-025-06159-z (PMC12125941; doi:10.1186/s12903-025-06159-z)
Supplement: Supplementary file 1 — Supplementary Material 1. [file 12903_2025_6159_MOESM1_ESM.pdf]

Impact of toothpaste use on the subgingival microbiome: a pilot randomized clinical trial

Margarita Iniesta,[a, b]# Viviane Vasconcelos,[b] Florencia Laciar,[b] Paula Matesanz,[a, b] Mariano Sanz,[a, b] David Herrera[a, b]

[a]ETEP (Etiology and Therapy of Periodontal and Peri-implant Diseases) Research Group, School of Dentistry, Complutense University of Madrid, Spain

[b] Section of Graduate Periodontology, Department of Dental Clinic Specialties, School of Dentistry, Complutense University of Madrid, Madrid, Spain

#Address correspondence to Margarita Iniesta, margaini@ucm.es.

Supplementary information

Supplementary Table 1. Subgingival microbiome of gingival inflammation at the phylum, genus, and species levels**.**

Supplementary Table 2. Dynamics of different genera according to toothpaste group, after 6 weeks of use of the respective toothpastes.

Supplementary Table 3. Dynamics of different species according to toothpaste group, after 6 weeks of use of the respective toothpastes.

Supplementary Figure 1. Rarefaction curves calculated for the observed amplifier sequence variants (ASVs) (A), Shannon diversity (B) and Faith's index (C).

Supplementary Figure 2. Heat map grouping samples by genus abundance.

Abundance data have been centered log-ratio transformed. Row labels add phylogenetic information. Colors represent the standardized abundances. Red indicates high abundance of the given genus, while blue indicates low abundance.

Supplementary Table 1. Subgingival microbiome of gingival inflammation at the phylum, genus, and species levels.

Supplementary Table 1a. Phylum level.

**Taxa**	**Baseline**	**6 weeks**
d__Bacteria;p__Bacteroidota	27.90967	25.72982
d__Bacteria;p__Firmicutes	19.37483	22.13607
d__Bacteria;p__Fusobacteriota	20.37417	19.70875
d__Bacteria;p__Proteobacteria	10.19133	10.80107
d__Bacteria;p__Actinobacteriota	9.40383	9.87464
d__Bacteria;p__Spirochaetota	5.4585	5.12196
d__Bacteria;p__Campilobacterota	3.24033	3.11804
d__Bacteria;p__Patescibacteria	2.768	2.34518
d__Bacteria;p__Synergistota	0.72867	0.76161
d__Bacteria;p__Desulfobacterota	0.3645	0.25857
d__Bacteria;p__Verrucomicrobiota	0.05017	0.09411
d__Bacteria;p__Chloroflexi	0.1245	0.04714
d__Bacteria;p__Elusimicrobiota	0.00583	0.00107
d__Eukaryota;p__Parabasalia	0.00133	0.00089
d__Archaea;p__Thermoplasmatota	0.00267	0
d__Archaea;p__Euryarchaeota	0.00083	0

Supplementary Table 1. Subgingival microbiome of gingival inflammation at the phylum, genus, and species levels.

Supplementary Table 1b. Genus level.

Taxa	Baseline	6 weeks
d__Bacteria;p__Fusobacteriota;c__Fusobacteriia;o__Fusobacteriales;f__Fusobacteriaceae;g__Fusobacterium	16.47417	15.415
d__Bacteria;p__Bacteroidota;c__Bacteroidia;o__Bacteroidales;f__Prevotellaceae;g__Prevotella	10.0705	7.92911
d__Bacteria;p__Bacteroidota;c__Bacteroidia;o__Flavobacteriales;f__Flavobacteriaceae;g__Capnocytophaga	6.07517	6.33089
d__Bacteria;p__Spirochaetota;c__Spirochaetia;o__Spirochaetales;f__Spirochaetaceae;g__Treponema	5.4585	5.11482
d__Bacteria;p__Bacteroidota;c__Bacteroidia;o__Bacteroidales;f__Porphyromonadaceae;g__Porphyromonas	4.31683	4.95036
d__Bacteria;p__Fusobacteriota;c__Fusobacteriia;o__Fusobacteriales;f__Leptotrichiaceae;g__Leptotrichia	3.67933	4.20268
d__Bacteria;p__Firmicutes;c__Bacilli;o__Lactobacillales;f__Streptococcaceae;g__Streptococcus	3.29783	3.9425
d__Bacteria;p__Actinobacteriota;c__Actinobacteria;o__Corynebacteriales;f__Corynebacteriaceae;g__Corynebacterium	2.43767	3.14857
d__Bacteria;p__Campilobacterota;c__Campylobacteria;o__Campylobacterales;f__Campylobacteraceae;g__Campylobacter	3.23467	3.09964
d__Bacteria;p__Firmicutes;c__Negativicutes;o__Veillonellales-Selenomonadales;f__Veillonellaceae;g__Veillonella	3.06633	2.96714
d__Bacteria;p__Actinobacteriota;c__Actinobacteria;o__Actinomycetales;f__Actinomycetaceae;g__Actinomyces	2.90117	2.92036
d__Bacteria;p__Proteobacteria;c__Gammaproteobacteria;o__Burkholderiales;f__Neisseriaceae;g__Neisseria	2.1225	2.88696
d__Bacteria;p__Actinobacteriota;c__Actinobacteria;o__Micrococcales;f__Micrococcaceae;g__Rothia	2.77733	2.41339
d__Bacteria;p__Firmicutes;c__Bacilli;o__Lactobacillales;f__Enterococcaceae;g__Enterococcus	1.37817	2.20375
d__Bacteria;p__Firmicutes;c__Bacilli;o__Lactobacillales;f__Carnobacteriaceae;g__Alkalibacterium	1.2725	2.11054
d__Bacteria;p__Bacteroidota;c__Bacteroidia;o__Bacteroidales;f__Tannerellaceae;g__Tannerella	1.59167	1.80607
d__Bacteria;p__Firmicutes;c__Negativicutes;o__Veillonellales-Selenomonadales;f__Selenomonadaceae;g__Selenomonas	1.602	1.68304
d__Bacteria;p__Proteobacteria;c__Gammaproteobacteria;o__Pasteurellales;f__Pasteurellaceae;g__Haemophilus	1.817	1.57786
d__Bacteria;p__Proteobacteria;c__Gammaproteobacteria;o__Cardiobacteriales;f__Cardiobacteriaceae;g__Cardiobacterium	1.474	1.45107
d__Bacteria;p__Proteobacteria;c__Gammaproteobacteria;o__Pasteurellales;f__Pasteurellaceae;g__Aggregatibacter	1.766	1.37464
d__Bacteria;p__Bacteroidota;c__Bacteroidia;o__Bacteroidales;f__Paludibacteraceae;g__F0058	1.93767	1.27286
d__Bacteria;p__Patescibacteria;c__Saccharimonadia;o__Saccharimonadales;f__Saccharimonadaceae;g__Saccharimonadaceae	1.30517	1.16839
d__Bacteria;p__Proteobacteria;c__Gammaproteobacteria;o__Burkholderiales;f__Neisseriaceae;g__Kingella	0.71717	0.97964
d__Bacteria;p__Bacteroidota;c__Bacteroidia;o__Bacteroidales;f__Prevotellaceae;g__Alloprevotella	1.81267	0.96732
d__Bacteria;p__Bacteroidota;c__Bacteroidia;o__Sphingobacteriales;f__Lentimicrobiaceae;g__Lentimicrobium	0.849	0.87786
d__Bacteria;p__Synergistota;c__Synergistia;o__Synergistales;f__Synergistaceae;g__Fretibacterium	0.72867	0.76161
d__Bacteria;p__Firmicutes;c__Bacilli;o__Exiguobacterales;f__Exiguobacteraceae;g__Exiguobacterium	0.50167	0.68518
d__Bacteria;p__Firmicutes;c__Bacilli;o__Bacillales;f__Bacillaceae;g__Anaerobacillus	0.4235	0.67625
d__Bacteria;p__Actinobacteriota;c__Actinobacteria;o__Actinomycetales;f__Actinomycetaceae;g__F0332	0.67717	0.63036
d__Bacteria;p__Proteobacteria;c__Gammaproteobacteria;o__Burkholderiales;f__Neisseriaceae;g__Eikenella	0.55033	0.61625

d__Bacteria;p__Firmicutes;c__Clostridia;o__Lachnospirales;f__Lachnospiraceae;g__Johnsonella	0.46867	0.59786
d__Bacteria;p__Firmicutes;c__Clostridia;o__Peptostreptococcales-Tissierellales;f__Peptostreptococcaceae;g__Filifactor	0.58283	0.58518
d__Bacteria;p__Firmicutes;c__Negativicutes;o__Veillonellales-Selenomonadales;f__Veillonellaceae;g__Dialister	0.9055	0.55411
d__Bacteria;p__Firmicutes;c__Clostridia;o__Clostridia_UCG-014;f__Clostridia_UCG-014;g__Clostridia_UCG-014	0.217	0.55304
d__Bacteria;p__Bacteroidota;c__Bacteroidia;o__Flavobacteriales;f__Weeksellaceae;g__Bergeyella	0.58067	0.54089
d__Bacteria;p__Proteobacteria;c__Gammaproteobacteria;o__Burkholderiales;f__Burkholderiaceae;g__Lautropia	0.80683	0.51411
d__Bacteria;p__Firmicutes;c__Bacilli;o__Mycoplasmatales;f__Mycoplasmataceae;g__Mycoplasma	0.31133	0.43964
d__Bacteria;p__Patescibacteria;c__Saccharimonadia;o__Saccharimonadales;f__Saccharimonadaceae;g__TM7x	0.3995	0.40839
d__Bacteria;p__Firmicutes;c__Clostridia;o__Lachnospirales;f__Lachnospiraceae;g__Lachnoanaerobaculum	0.36667	0.39179
d__Bacteria;p__Firmicutes;c__Clostridia;o__Lachnospirales;f__Lachnospiraceae;g__Catonella	0.346	0.34339
d__Bacteria;p__Proteobacteria;c__Alphaproteobacteria;o__Rhodobacterales;f__Rhodobacteraceae;g__Ketogulonicigenium	0.19033	0.33554
d__Bacteria;p__Firmicutes;c__Clostridia;o__Peptostreptococcales-Tissierellales;f__Peptostreptococcaceae;g__[Eubacterium]_yurii_group	0.31233	0.30357
d__Bacteria;p__Firmicutes;c__Bacilli;o__Bacillales;f__Salisediminibacteriaceae;g__Salipaludibacillus	0.2695	0.30071
d__Bacteria;p__Firmicutes;c__Clostridia;o__Peptostreptococcales-Tissierellales;f__Peptostreptococcales-Tissierellales;g__Parvimonas	0.42883	0.29107
d__Bacteria;p__Actinobacteriota;c__Actinobacteria;o__Micrococcales;f__Cellulomonadaceae;g__Actinotalea	0.11333	0.27375
d__Bacteria;p__Proteobacteria;c__Gammaproteobacteria;o__Alteromonadales;f__Alteromonadaceae;g__Alishewanella	0.16417	0.27125
d__Bacteria;p__Firmicutes;c__Bacilli;o__Staphylococcales;f__Gemellaceae;g__Gemella	0.19683	0.25268
d__Bacteria;p__Patescibacteria;c__Saccharimonadia;o__Saccharimonadales;f__Saccharimonadales;g__Saccharimonadales	0.287	0.24911
d__Bacteria;p__Firmicutes;c__Bacilli;o__Lactobacillales;f__Carnobacteriaceae;g__Granulicatella	0.12167	0.245
d__Bacteria;p__Firmicutes;c__Clostridia;o__Lachnospirales;f__Lachnospiraceae;g__Oribacterium	0.24033	0.21696
d__Bacteria;p__Actinobacteriota;c__Actinobacteria;o__Propionibacteriales;f__Propionibacteriaceae;g__Pseudopropionibacterium	0.158	0.21036
d__Bacteria;p__Proteobacteria;c__Alphaproteobacteria;o__Rhodobacterales;f__Rhodobacteraceae;g__Paracoccus	0.07967	0.20054
d__Bacteria;p__Bacteroidota;c__Bacteroidia;o__Bacteroidales;f__Paludibacteraceae;g__Paludibacter	0.11283	0.18161
d__Bacteria;p__Firmicutes;c__Clostridia;o__Lachnospirales;f__Defluviitaleaceae;g__Defluviitaleaceae_UCG-011	0.16983	0.18036
d__Bacteria;p__Actinobacteriota;c__Coriobacteriia;o__Coriobacteriales;f__Atopobiaceae;g__Olsenella	0.16567	0.17982
d__Bacteria;p__Bacteroidota;c__Bacteroidia;o__Bacteroidales;f__Rikenellaceae;g__Rikenellaceae_RC9_gut_group	0.20167	0.17071
d__Bacteria;p__Firmicutes;c__Negativicutes;o__Veillonellales-Selenomonadales;f__Selenomonadaceae;__	0.19433	0.16679
d__Bacteria;p__Desulfobacterota;c__Desulfobulbia;o__Desulfobulbales;f__Desulfobulbaceae;g__Desulfobulbus	0.33067	0.16375
d__Bacteria;p__Proteobacteria;c__Gammaproteobacteria;o__Burkholderiales;f__Neisseriaceae;__	0.017	0.16321
d__Bacteria;p__Patescibacteria;c__Saccharimonadia;o__Saccharimonadales;f__Saccharimonadaceae;g__Candidatus_Saccharimonas	0.21	0.16196
d__Bacteria;p__Patescibacteria;c__Gracilibacteria;o__JGI_0000069-P22;f__JGI_0000069-P22;g__JGI_0000069-P22	0.057	0.15571
d__Bacteria;p__Firmicutes;c__Clostridia;o__Peptostreptococcales-Tissierellales;f__Anaerovoracaceae;g__[Eubacterium]_brachy_group	0.16483	0.14589
d__Bacteria;p__Firmicutes;c__Negativicutes;o__Veillonellales-Selenomonadales;f__Selenomonadaceae;g__Centipeda	0.22417	0.14393
d__Bacteria;p__Bacteroidota;c__Bacteroidia;o__Bacteroidales;f__Tannerellaceae;g__Parabacteroides	0.01617	0.13893
d__Bacteria;p__Firmicutes;c__Bacilli;o__Lactobacillales;f__Lactobacillaceae;g__Lactobacillus	0.323	0.13786
d__Bacteria;p__Firmicutes;c__Clostridia;o__Lachnospirales;f__Lachnospiraceae;g__Anaerocolumna	0.064	0.13589

d__Bacteria;p__Firmicutes;c__Bacilli;o__Acholeplasmatales;f__Acholeplasmataceae;g__Acholeplasma	0.2945	0.12679
d__Bacteria;p__Patescibacteria;c__Gracilibacteria;o__Absconditabacteriales_(SR1);f__Absconditabacteriales_(SR1);g__Absconditabacteriales_(SR1)	0.28233	0.10804
d__Bacteria;p__Bacteroidota;c__Bacteroidia;o__Bacteroidales;f__uncultured;g__uncultured	0.01317	0.10643
d__Bacteria;p__Firmicutes;c__Clostridia;o__Lachnospirales;f__Lachnospiraceae;g__Lachnoclostridium	0.04867	0.10071
d__Bacteria;p__Bacteroidota;c__Bacteroidia;o__Bacteroidales;f__Dysgonomonadaceae;g__Dysgonomonas	0.042	0.09857
d__Bacteria;p__Verrucomicrobiota;c__Verrucomicrobiae;o__Opitutales;f__Opitutaceae;g__IMCC26134	0.04567	0.09339
d__Bacteria;p__Firmicutes;c__Bacilli;o__Paenibacillales;f__Paenibacillaceae;g__Paenibacillus	0.0265	0.09214
d__Bacteria;p__Firmicutes;c__Clostridia;o__Lachnospirales;f__Defluviitaleaceae;g__Defluviitalea	0.06	0.09161
d__Bacteria;p__Fusobacteriota;c__Fusobacteriia;o__Fusobacteriales;f__Leptotrichiaceae;g__uncultured	0.2065	0.09107
d__Bacteria;p__Firmicutes;c__Negativicutes;o__Veillonellales-Selenomonadales;f__Veillonellaceae;g__Anaeroglobus	0.05783	0.08929
d__Bacteria;p__Bacteroidota;c__Bacteroidia;o__Flavobacteriales;f__Flavobacteriaceae;g__Flavobacterium	0.04417	0.08821
d__Bacteria;p__Proteobacteria;c__Gammaproteobacteria;o__Burkholderiales;f__Rhodocyclaceae;g__Propionivibrio	0.106	0.08411
d__Bacteria;p__Firmicutes;c__Bacilli;o__Lactobacillales;f__Aerococcaceae;g__Abiotrophia	0.09717	0.08393
d__Bacteria;p__Firmicutes;c__Clostridia;o__Peptococcales;f__Peptococcaceae;g__Peptococcus	0.10233	0.07893
d__Bacteria;p__Bacteroidota;c__Bacteroidia;o__Bacteroidales;f__Bacteroidales_Incertae_Sedis;g__Phocaeicola	0.1375	0.07357
d__Bacteria;p__Proteobacteria;c__Gammaproteobacteria;o__Pseudomonadales;f__Pseudomonadaceae;g__Pseudomonas	0.06667	0.06839
d__Bacteria;p__Proteobacteria;c__Gammaproteobacteria;o__Burkholderiales;f__Comamonadaceae;g__Comamonas	0.093	0.06643
d__Bacteria;p__Firmicutes;c__Bacilli;o__Lactobacillales;f__Streptococcaceae;g__Lactococcus	0.0525	0.065
d__Bacteria;p__Firmicutes;c__Clostridia;o__Peptostreptococcales-Tissierellales;f__Anaerovoracaceae;g__[Eubacterium]_saphenum_group	0.05683	0.06321
d__Bacteria;p__Firmicutes;c__Clostridia;o__Peptostreptococcales-Tissierellales;f__Peptostreptococcales-Tissierellales;g__uncultured	0.03117	0.06143
d__Bacteria;p__Firmicutes;c__Bacilli;o__Erysipelotrichales;f__Erysipelotrichaceae;g__Erysipelothrix	0.03483	0.06125
d__Bacteria;p__Firmicutes;c__Clostridia;o__Peptostreptococcales-Tissierellales;f__Anaerovoracaceae;g__uncultured	0.07133	0.05732
d__Bacteria;p__Firmicutes;c__Bacilli;o__Lactobacillales;f__uncultured;g__uncultured	0.016	0.05714
d__Bacteria;p__Desulfobacterota;c__Desulfovibrionia;o__Desulfovibrionales;f__Desulfoplanaceae;g__Desulfoplanes	0	0.05554
d__Bacteria;p__Firmicutes;c__Clostridia;o__Peptostreptococcales-Tissierellales;f__Peptostreptococcaceae;g__Acetoanaerobium	0.01317	0.05429
d__Bacteria;p__Firmicutes;c__Negativicutes;o__Veillonellales-Selenomonadales;f__Veillonellaceae;g__Megasphaera	0.01233	0.05393
d__Bacteria;p__Firmicutes;c__Clostridia;o__Peptostreptococcales-Tissierellales;f__Peptostreptococcales-Tissierellales;__	0.0405	0.05321
d__Bacteria;p__Bacteroidota;c__Bacteroidia;o__Bacteroidales;f__Prevotellaceae;__	0.02483	0.05
d__Bacteria;p__Firmicutes;c__Clostridia;o__Peptostreptococcales-Tissierellales;f__Peptostreptococcaceae;g__Peptostreptococcus	0.09533	0.04911
d__Bacteria;p__Chloroflexi;c__Anaerolineae;o__Anaerolineales;f__Anaerolineaceae;g__Flexilinea	0.1245	0.04714
d__Bacteria;p__Firmicutes;c__Clostridia;o__Lachnospirales;f__Lachnospiraceae;__	0.07583	0.04089
d__Bacteria;p__Firmicutes;c__Clostridia;o__Peptostreptococcales-Tissierellales;f__Peptostreptococcaceae;g__Peptoanaerobacter	0.05733	0.04071
d__Bacteria;p__Firmicutes;c__Clostridia;o__Peptostreptococcales-Tissierellales;f__Fusibacteraceae;g__Fusibacter	0.01533	0.04036
d__Bacteria;p__Firmicutes;c__Clostridia;o__Lachnospirales;f__Lachnospiraceae;g__Roseburia	0.02967	0.03911
d__Bacteria;p__Firmicutes;c__Clostridia;o__Lachnospirales;f__Lachnospiraceae;g__uncultured	0.05167	0.03857
d__Bacteria;p__Firmicutes;c__Bacilli;o__Izemoplasmatales;f__Izemoplasmatales;g__Izemoplasmatales	0.02483	0.03536

d__Bacteria;p__Actinobacteriota;c__Coriobacteriia;o__Coriobacteriales;f__Atopobiaceae;g__Atopobium	0.09067	0.03464
d__Bacteria;p__Firmicutes;c__Clostridia;o__Oscillospirales;f__Ethanoligenenaceae;g__Ethanoligenens	0.00333	0.03304
d__Bacteria;p__Firmicutes;c__Bacilli;o__Erysipelotrichales;f__Erysipelotrichaceae;g__Solobacterium	0.01717	0.03286
d__Bacteria;p__Patescibacteria;c__Saccharimonadia;o__Saccharimonadales;__;__	0.04033	0.03268
d__Bacteria;p__Patescibacteria;c__Gracilibacteria;o__Gracilibacteria;f__Gracilibacteria;g__Gracilibacteria	0.00583	0.03179
d__Bacteria;p__Firmicutes;c__Clostridia;o__Clostridia_vadinBB60_group;f__Clostridia_vadinBB60_group;g__Clostridia_vadinBB60_group	0.03067	0.02946
d__Bacteria;p__Bacteroidota;c__Bacteroidia;o__Bacteroidales;f__Marinifilaceae;g__Odoribacter	0.00667	0.02732
d__Bacteria;p__Bacteroidota;c__Bacteroidia;o__Bacteroidales;f__Bacteroidaceae;g__Bacteroides	0.0185	0.02679
d__Bacteria;p__Firmicutes;c__Clostridia;o__Peptostreptococcales-Tissierellales;f__Peptostreptococcales-Tissierellales;g__Serpentinicella	0.0055	0.02589
d__Bacteria;p__Firmicutes;c__Clostridia;o__Peptostreptococcales-Tissierellales;f__Peptostreptococcales-Tissierellales;g__W5053	0.068	0.025
d__Bacteria;p__Firmicutes;c__Clostridia;o__Peptostreptococcales-Tissierellales;f__Anaerovoracaceae;g__[Eubacterium]_nodatum_group	0.064	0.02375
d__Bacteria;p__Firmicutes;c__Clostridia;o__Peptostreptococcales-Tissierellales;f__Thermotaleaceae;g__Geosporobacter	0.01583	0.02339
d__Bacteria;p__Firmicutes;c__Clostridia;o__Clostridiales;f__Clostridiaceae;g__Clostridium_sensu_stricto_3	0.00883	0.02321
d__Bacteria;p__Desulfobacterota;c__Desulfovibrionia;o__Desulfovibrionales;f__Desulfovibrionaceae;g__Desulfovibrio	0.02483	0.02304
d__Bacteria;p__Bacteroidota;c__Bacteroidia;o__Flavobacteriales;f__Weeksellaceae;g__Chryseobacterium	0.00783	0.0225
d__Bacteria;p__Proteobacteria;c__Alphaproteobacteria;o__Rhodospirillales;f__uncultured;g__uncultured	0.0045	0.02214
d__Bacteria;p__Proteobacteria;c__Gammaproteobacteria;o__Burkholderiales;f__Comamonadaceae;g__Brachymonas	0.0155	0.02196
d__Bacteria;p__Firmicutes;c__Clostridia;o__Oscillospirales;f__Ethanoligenenaceae;g__Incertae_Sedis	0.00083	0.02071
d__Bacteria;p__Proteobacteria;c__Gammaproteobacteria;o__Xanthomonadales;f__Xanthomonadaceae;g__Xanthomonas	0.04233	0.02054
d__Bacteria;p__Proteobacteria;c__Alphaproteobacteria;o__Caulobacterales;f__Caulobacteraceae;g__Brevundimonas	0.0075	0.01857
d__Bacteria;p__Proteobacteria;c__Alphaproteobacteria;o__Acetobacterales;f__Acetobacteraceae;g__Acetobacter	0	0.01857
d__Bacteria;p__Patescibacteria;c__Saccharimonadia;o__Saccharimonadales;f__Saccharimonadaceae;__	0.01033	0.01839
d__Bacteria;p__Actinobacteriota;c__Actinobacteria;o__Bifidobacteriales;f__Bifidobacteriaceae;g__Scardovia	0.042	0.01768
d__Bacteria;p__Firmicutes;c__Negativicutes;o__Veillonellales-Selenomonadales;f__Selenomonadaceae;g__uncultured	0.065	0.01732
d__Bacteria;p__Firmicutes;c__Clostridia;o__Peptostreptococcales-Tissierellales;f__Peptostreptococcales-Tissierellales;g__Alkaliphilus	0.01433	0.01732
d__Bacteria;p__Firmicutes;c__Clostridia;o__Oscillospirales;f__Ruminococcaceae;g__Caproiciproducens	0.00033	0.01696
d__Bacteria;p__Firmicutes;c__Clostridia;o__Lachnospirales;f__Lachnospiraceae;g__Stomatobaculum	0.04083	0.01536
d__Bacteria;p__Proteobacteria;c__Alphaproteobacteria;o__Rhodobacterales;f__Rhodobacteraceae;__	0.00517	0.01536
d__Bacteria;p__Proteobacteria;c__Gammaproteobacteria;o__Enterobacterales;f__Enterobacteriaceae;__	0.00417	0.01411
d__Bacteria;p__Bacteroidota;c__Bacteroidia;o__Cytophagales;f__Cyclobacteriaceae;g__Mongoliitalea	0.00633	0.01286
d__Bacteria;p__Desulfobacterota;c__Desulfovibrionia;o__Desulfovibrionales;f__Desulfomicrobiaceae;g__Desulfomicrobium	0.00833	0.01179
d__Bacteria;p__Bacteroidota;c__Bacteroidia;o__Bacteroidales;f__Rikenellaceae;g__uncultured	0.014	0.01089
d__Bacteria;p__Firmicutes;c__Bacilli;o__Staphylococcales;f__Staphylococcaceae;g__Staphylococcus	0.01683	0.01054
d__Bacteria;p__Proteobacteria;c__Gammaproteobacteria;o__Alteromonadales;f__Alteromonadales_Incertae_Sedis;g__Alkalimonas	0.01167	0.01
d__Bacteria;p__Actinobacteriota;c__Actinobacteria;o__Micrococcales;f__Microbacteriaceae;g__Microbacterium	0.009	0.01
d__Bacteria;p__Campilobacterota;c__Campylobacteria;o__Campylobacterales;f__Helicobacteraceae;g__Wolinella	0.00467	0.00929

d__Bacteria;p__Campilobacterota;c__Campylobacteria;o__Campylobacterales;f__Rs-M59_termite_group;g__Rs-M59_termite_group	0.001	0.00911
d__Bacteria;p__Firmicutes;c__Bacilli;o__Lactobacillales;__;__	0.01333	0.00893
d__Bacteria;p__Firmicutes;c__Clostridia;o__Oscillospirales;f__Oscillospirales;g__Hydrogenoanaerobacterium	0.00567	0.00893
d__Bacteria;p__Proteobacteria;c__Alphaproteobacteria;o__Rhodospirillales;f__Rhodospirillaceae;g__Candidatus_Riegeria	0.005	0.00893
d__Bacteria;p__Bacteroidota;c__Bacteroidia;o__Bacteroidales;f__Marinilabiliaceae;g__Alkaliflexus	0.00183	0.00893
d__Bacteria;p__Actinobacteriota;c__Actinobacteria;o__Bifidobacteriales;f__Bifidobacteriaceae;g__Parascardovia	0.00533	0.00875
d__Bacteria;p__Firmicutes;c__Bacilli;o__Erysipelotrichales;f__Erysipelotrichaceae;g__Erysipelotrichaceae_UCG-006	0.01017	0.00857
d__Bacteria;p__Firmicutes;c__Clostridia;o__Peptostreptococcales-Tissierellales;f__Anaerovoracaceae;g__Family_XIII_UCG-001	0.00767	0.00839
d__Bacteria;p__Proteobacteria;c__Gammaproteobacteria;o__Burkholderiales;f__Burkholderiaceae;g__Burkholderia-Caballeronia-Paraburkholderia	0.0065	0.00821
d__Bacteria;p__Firmicutes;c__Clostridia;o__Eubacteriales;f__Eubacteriaceae;g__Pseudoramibacter	0.027	0.00768
d__Bacteria;p__Proteobacteria;c__Gammaproteobacteria;o__Oceanospirillales;f__Nitrincolaceae;g__Nitrincola	0.00333	0.0075
d__Bacteria;p__Firmicutes;c__Clostridia;o__Lachnospirales;f__Lachnospiraceae;g__Butyrivibrio	0.01817	0.00714
d__Bacteria;p__Spirochaetota;c__Spirochaetia;o__Spirochaetales;f__Spirochaetaceae;g__Sediminispirochaeta	0	0.00714
d__Bacteria;p__Proteobacteria;c__Alphaproteobacteria;o__Sphingomonadales;f__Sphingomonadaceae;g__Sphingobium	0.00317	0.00696
d__Bacteria;p__Patescibacteria;c__Microgenomatia;o__Candidatus_Pacebacteria;f__Candidatus_Pacebacteria;g__Candidatus_Pacebacteria	0.01667	0.00607
d__Bacteria;p__Actinobacteriota;c__Actinobacteria;o__Propionibacteriales;f__Propionibacteriaceae;g__Aestuariimicrobium	0.00117	0.00607
d__Bacteria;p__Bacteroidota;c__Bacteroidia;o__Bacteroidales;f__Prevotellaceae;g__Prevotellaceae	0.0015	0.00589
d__Bacteria;p__Proteobacteria;c__Alphaproteobacteria;__;__;__	0.00083	0.00589
d__Bacteria;p__Firmicutes;c__Clostridia;o__Oscillospirales;f__Ruminococcaceae;g__Anaerofilum	0.00183	0.00571
d__Bacteria;p__Proteobacteria;c__Alphaproteobacteria;o__Rhodospirillales;__;__	0.00067	0.00554
d__Bacteria;p__Proteobacteria;c__Gammaproteobacteria;o__Pseudomonadales;f__Moraxellaceae;g__Acinetobacter	0.0035	0.00536
d__Bacteria;p__Proteobacteria;c__Gammaproteobacteria;o__Alteromonadales;f__Shewanellaceae;g__Shewanella	0.001	0.00518
d__Bacteria;p__Firmicutes;c__Negativicutes;o__Veillonellales-Selenomonadales;f__Veillonellaceae;__	0.0075	0.005
d__Bacteria;p__Firmicutes;c__Clostridia;o__Lachnospirales;f__Lachnospiraceae;g__Shuttleworthia	0.05083	0.00482
d__Bacteria;p__Desulfobacterota;c__Desulfuromonadia;o__Bradymonadales;f__Bradymonadales;g__Bradymonadales	0.0005	0.00464
d__Bacteria;p__Actinobacteriota;c__Actinobacteria;o__Propionibacteriales;f__Propionibacteriaceae;g__Cutibacterium	0.00133	0.00429
d__Bacteria;p__Proteobacteria;c__Alphaproteobacteria;o__Rhizobiales;f__Rhizobiaceae;g__Allorhizobium-Neorhizobium-Pararhizobium-Rhizobium	0.00817	0.00411
d__Bacteria;p__Firmicutes;c__Clostridia;o__Lachnospirales;f__Lachnospiraceae;g__Anaerostignum	0.00317	0.00411
d__Bacteria;p__Bacteroidota;c__Bacteroidia;o__Cytophagales;f__Spirosomaceae;g__Dyadobacter	0	0.00411
d__Bacteria;p__Bacteroidota;c__Bacteroidia;o__Bacteroidales;f__Paludibacteraceae;g__uncultured	0.00283	0.00393
d__Bacteria;p__Actinobacteriota;c__Actinobacteria;o__Propionibacteriales;f__Propionibacteriaceae;g__Tessaracoccus	0.00117	0.00393
d__Bacteria;p__Firmicutes;c__Clostridia;o__Oscillospirales;f__Oscillospiraceae;g__Oscillibacter	0.00167	0.00375
d__Bacteria;p__Bacteroidota;c__Bacteroidia;o__Sphingobacteriales;f__Sphingobacteriaceae;g__Sphingobacterium	0.00067	0.00357
d__Bacteria;p__Bacteroidota;c__Bacteroidia;__;__;__	0.00083	0.00339
d__Bacteria;p__Firmicutes;c__Bacilli;o__Erysipelotrichales;f__Erysipelotrichaceae;g__Bulleidia	0.00633	0.00286
d__Bacteria;p__Bacteroidota;c__Bacteroidia;o__Cytophagales;f__Spirosomaceae;g__Flectobacillus	0.00033	0.00286

d__Bacteria;p__Bacteroidota;c__Bacteroidia;o__Bacteroidales;f__Prevotellaceae;g__Prevotellaceae_UCG-004	0.00333	0.00268
d__Bacteria;p__Firmicutes;c__Clostridia;o__Peptostreptococcales-Tissierellales;f__Anaerovoracaceae;g__Mogibacterium	0.006	0.0025
d__Bacteria;p__Firmicutes;c__Bacilli;o__RF39;f__RF39;g__RF39	0.00483	0.0025
d__Bacteria;p__Patescibacteria;c__Saccharimonadia;o__Saccharimonadales;f__Saccharimonadaceae;g__TM7a	0.15483	0.00232
d__Bacteria;p__Firmicutes;c__Clostridia;o__Lachnospirales;f__Lachnospiraceae;g__Lachnotalea	0.00333	0.00232
d__Bacteria;p__Bacteroidota;c__Bacteroidia;o__Flavobacteriales;f__Crocinitomicaceae;__	0.00067	0.00232
d__Bacteria;p__Patescibacteria;c__Dojkabacteria;o__Dojkabacteria;f__Dojkabacteria;g__Dojkabacteria	0.00017	0.00232
d__Bacteria;p__Firmicutes;c__Bacilli;o__Bacillales;f__Sporolactobacillaceae;g__Sporolactobacillus	0	0.00232
d__Bacteria;p__Actinobacteriota;c__Actinobacteria;o__Bifidobacteriales;f__Bifidobacteriaceae;g__Bifidobacterium	0.0075	0.00196
d__Bacteria;p__Actinobacteriota;c__Actinobacteria;o__Propionibacteriales;f__Propionibacteriaceae;g__Propionibacterium	0.00383	0.00196
d__Bacteria;p__Proteobacteria;c__Alphaproteobacteria;o__Sphingomonadales;f__Sphingomonadaceae;g__Novosphingobium	0.00267	0.00196
d__Bacteria;p__Bacteroidota;c__Bacteroidia;o__Flavobacteriales;f__Cryomorphaceae;g__Owenweeksia	0.0015	0.00196
d__Bacteria;p__Bacteroidota;c__Bacteroidia;o__Cytophagales;f__Spirosomaceae;g__Persicitalea	0.0005	0.00161
d__Bacteria;p__Actinobacteriota;c__Actinobacteria;o__Frankiales;f__Geodermatophilaceae;g__Modestobacter	0	0.00161
d__Bacteria;p__Bacteroidota;c__Bacteroidia;o__Flavobacteriales;f__Weeksellaceae;g__uncultured	0	0.00161
d__Bacteria;p__Proteobacteria;c__Gammaproteobacteria;o__Cardiobacteriales;f__Cardiobacteriaceae;g__Suttonella	0	0.00143
d__Bacteria;p__Proteobacteria;c__Gammaproteobacteria;o__Enterobacterales;f__Erwiniaceae;__	0	0.00143
d__Bacteria;p__Bacteroidota;c__Bacteroidia;o__Bacteroidales;f__Prevotellaceae;g__uncultured	0.01317	0.00125
d__Bacteria;p__Actinobacteriota;c__Actinobacteria;o__Micrococcales;f__Cellulomonadaceae;g__Cellulomonas	0.0025	0.00125
d__Bacteria;p__Proteobacteria;c__Gammaproteobacteria;o__Cellvibrionales;f__Cellvibrionaceae;g__Cellvibrio	0.001	0.00125
d__Bacteria;p__Actinobacteriota;c__Coriobacteriia;o__Coriobacteriales;f__Eggerthellaceae;__	0	0.00125
d__Bacteria;p__Firmicutes;c__Clostridia;o__Oscillospirales;f__Oscillospiraceae;__	0	0.00125
d__Bacteria;p__Proteobacteria;c__Alphaproteobacteria;o__Sphingomonadales;f__Sphingomonadaceae;g__Rhizorhapis	0	0.00125
d__Bacteria;p__Elusimicrobiota;c__Elusimicrobia;o__Lineage_IV;f__Lineage_IV;g__Lineage_IV	0.00583	0.00107
d__Bacteria;p__Firmicutes;c__Clostridia;o__Oscillospirales;f__Ruminococcaceae;g__Ruminococcus	0.0015	0.00107
d__Bacteria;p__Proteobacteria;c__Alphaproteobacteria;o__Sphingomonadales;f__Sphingomonadaceae;g__Sphingomonas	0.0015	0.00107
d__Bacteria;p__Bacteroidota;c__Bacteroidia;o__Chitinophagales;f__Chitinophagaceae;g__Taibaiella	0.0005	0.00107
d__Bacteria;p__Actinobacteriota;c__Actinobacteria;o__Corynebacteriales;f__Corynebacteriaceae;g__Lawsonella	0	0.00107
d__Bacteria;p__Firmicutes;c__Clostridia;o__Oscillospirales;f__Ruminococcaceae;g__Candidatus_Soleaferrea	0	0.00107
d__Bacteria;p__Proteobacteria;c__Alphaproteobacteria;o__Azospirillales;f__Azospirillaceae;g__Nitrospirillum	0.00133	0.00089
d__Eukaryota;p__Parabasalia;c__Trichomonadea;o__Trichomonadea;f__Trichomonadea;g__Trichomonas	0.00133	0.00089
d__Bacteria;p__Actinobacteriota;c__Actinobacteria;o__Micrococcales;f__Microbacteriaceae;__	0	0.00089
d__Bacteria;p__Firmicutes;c__Clostridia;o__Peptostreptococcales-Tissierellales;f__Peptostreptococcaceae;__	0	0.00089
d__Bacteria;p__Firmicutes;c__Clostridia;o__Peptostreptococcales-Tissierellales;f__Peptostreptococcales-Tissierellales;g__Anaerococcus	0	0.00089
d__Bacteria;p__Proteobacteria;c__Gammaproteobacteria;o__Enterobacterales;f__Erwiniaceae;g__Pantoea	0	0.00089
d__Bacteria;p__Proteobacteria;c__Gammaproteobacteria;o__Pasteurellales;f__Pasteurellaceae;g__Actinobacillus	0.015	0.00071

d__Bacteria;p__Actinobacteriota;c__Actinobacteria;o__Propionibacteriales;f__Propionibacteriaceae;g__Acidipropionibacterium	0	0.00071
d__Bacteria;p__Proteobacteria;c__Alphaproteobacteria;o__Rhizobiales;f__Xanthobacteraceae;g__Pseudolabrys	0	0.00071
d__Bacteria;p__Firmicutes;c__Clostridia;o__Lachnospirales;f__Lachnospiraceae;g__Howardella	0.00667	0.00054
d__Bacteria;p__Firmicutes;c__Bacilli;o__Bacillales;f__Bacillaceae;g__Bacillus	0.003	0.00054
d__Bacteria;p__Verrucomicrobiota;c__Verrucomicrobiae;o__Opitutales;f__Puniceicoccaceae;g__Cerasicoccus	0.00217	0.00054
d__Bacteria;p__Proteobacteria;c__Gammaproteobacteria;o__Aeromonadales;f__Aeromonadaceae;g__Aeromonas	0.00133	0.00054
d__Bacteria;p__Actinobacteriota;c__Actinobacteria;o__Micrococcales;f__Microbacteriaceae;g__Leucobacter	0.00083	0.00054
d__Bacteria;p__Actinobacteriota;c__Actinobacteria;o__Micrococcales;__;__	0.0005	0.00054
d__Bacteria;p__Actinobacteriota;c__Actinobacteria;o__Bifidobacteriales;f__Bifidobacteriaceae;g__Gardnerella	0	0.00054
d__Bacteria;p__Firmicutes;c__Bacilli;o__Erysipelotrichales;f__Erysipelatoclostridiaceae;g__UCG-004	0	0.00054
d__Bacteria;p__Proteobacteria;c__Gammaproteobacteria;o__Burkholderiales;f__Comamonadaceae;g__Aquabacterium	0	0.00054
d__Bacteria;p__Proteobacteria;c__Gammaproteobacteria;o__Burkholderiales;f__Comamonadaceae;g__Hydrogenophaga	0	0.00054
d__Bacteria;p__Firmicutes;c__Clostridia;o__Peptostreptococcales-Tissierellales;f__Anaerovoracaceae;g__Amnipila	0	0.00036
d__Bacteria;p__Proteobacteria;c__Alphaproteobacteria;o__Rhizobiales;f__Devosiaceae;g__Pelagibacterium	0	0.00036
d__Bacteria;p__Proteobacteria;c__Gammaproteobacteria;o__Burkholderiales;f__Burkholderiaceae;g__Cupriavidus	0	0.00036
d__Bacteria;p__Proteobacteria;c__Gammaproteobacteria;o__Pasteurellales;f__Pasteurellaceae;__	0	0.00036
d__Bacteria;p__Proteobacteria;c__Alphaproteobacteria;o__Acetobacterales;f__Acetobacteraceae;g__Roseomonas	0.0005	0.00018
d__Bacteria;p__Firmicutes;c__Clostridia;o__Oscillospirales;f__Oscillospiraceae;g__Colidextribacter	0	0.00018
d__Bacteria;p__Firmicutes;c__Clostridia;o__Oscillospirales;f__UCG-011;g__UCG-011	0	0.00018
d__Bacteria;p__Firmicutes;c__Clostridia;o__Peptostreptococcales-Tissierellales;f__Peptostreptococcales-Tissierellales;g__Peptoniphilus	0	0.00018
d__Bacteria;p__Firmicutes;c__Negativicutes;o__Acidaminococcales;f__Acidaminococcaceae;g__Acidaminococcus	0	0.00018
d__Bacteria;p__Proteobacteria;c__Alphaproteobacteria;o__Rhizobiales;f__Stappiaceae;g__Pannonibacter	0	0.00018
d__Bacteria;p__Proteobacteria;c__Gammaproteobacteria;o__Pseudomonadales;f__Moraxellaceae;g__Moraxella	0.0625	0
d__Bacteria;p__Fusobacteriota;c__Fusobacteriia;o__Fusobacteriales;f__Leptotrichiaceae;g__Streptobacillus	0.015	0
d__Bacteria;p__Firmicutes;c__Bacilli;o__Bacillales;f__Bacillaceae;__	0.01067	0
d__Bacteria;p__Firmicutes;c__Bacilli;o__Mycoplasmatales;f__Mycoplasmataceae;g__Ureaplasma	0.00683	0
d__Bacteria;p__Proteobacteria;c__Gammaproteobacteria;o__Pasteurellales;f__Pasteurellaceae;g__Histophilus	0.00467	0
d__Bacteria;p__Firmicutes;c__Bacilli;o__Erysipelotrichales;f__Erysipelatoclostridiaceae;g__Eggerthia	0.00367	0
d__Bacteria;p__Proteobacteria;c__Gammaproteobacteria;o__Enterobacterales;f__Enterobacteriaceae;g__Enterobacter	0.00283	0
d__Archaea;p__Thermoplasmatota;c__Thermoplasmata;o__uncultured;f__uncultured;g__uncultured	0.00267	0
d__Bacteria;p__Proteobacteria;c__Gammaproteobacteria;o__Enterobacterales;f__Enterobacteriaceae;g__Escherichia-Shigella	0.0025	0
d__Bacteria;p__Verrucomicrobiota;c__Verrucomicrobiae;o__Opitutales;f__Puniceicoccaceae;g__Verruc-01	0.00233	0
d__Bacteria;p__Firmicutes;c__Bacilli;o__Bacillales;f__Planococcaceae;g__Rummeliibacillus	0.00217	0
d__Bacteria;p__Actinobacteriota;c__Coriobacteriia;o__Coriobacteriales;f__Eggerthellaceae;g__Slackia	0.002	0
d__Bacteria;p__Firmicutes;c__Clostridia;o__Peptostreptococcales-Tissierellales;f__Anaerovoracaceae;g__Family_XIII_AD3011_group	0.002	0
d__Bacteria;p__Bacteroidota;c__Bacteroidia;o__Bacteroidales;__;__	0.00183	0

d__Bacteria;p__Actinobacteriota;c__Coriobacteriia;o__Coriobacteriales;f__Eggerthellaceae;g__Cryptobacterium	0.00167	0
d__Bacteria;p__Actinobacteriota;c__Actinobacteria;o__Micrococcales;f__Dermatophilaceae;g__Piscicoccus	0.0015	0
d__Bacteria;p__Actinobacteriota;c__Actinobacteria;o__Actinomycetales;f__Actinomycetaceae;g__Trueperella	0.00133	0
d__Bacteria;p__Firmicutes;c__Clostridia;o__Monoglobales;f__Monoglobaceae;g__Monoglobus	0.00117	0
d__Bacteria;p__Proteobacteria;c__Gammaproteobacteria;o__Burkholderiales;f__Oxalobacteraceae;g__Herbaspirillum	0.001	0
d__Bacteria;p__Proteobacteria;c__Gammaproteobacteria;o__Coxiellales;f__Coxiellaceae;g__Coxiella	0.001	0
d__Archaea;p__Euryarchaeota;c__Methanobacteria;o__Methanobacteriales;f__Methanobacteriaceae;g__Methanobrevibacter	0.00083	0
d__Bacteria;p__Bacteroidota;c__Bacteroidia;o__Bacteroidales;f__Tannerellaceae;g__uncultured	0.0005	0
d__Bacteria;p__Bacteroidota;c__Bacteroidia;o__Flavobacteriales;f__Crocinitomicaceae;g__Wandonia	0.0005	0
d__Bacteria;p__Proteobacteria;c__Gammaproteobacteria;o__Oceanospirillales;f__Halomonadaceae;g__Halomonas	0.0005	0
d__Bacteria;p__Firmicutes;c__Clostridia;o__Proteinivoracales;f__uncultured;g__uncultured	0.00033	0
d__Bacteria;p__Fusobacteriota;c__Fusobacteriia;o__Fusobacteriales;f__Leptotrichiaceae;__	0.00033	0
d__Bacteria;p__Proteobacteria;c__Gammaproteobacteria;o__Burkholderiales;f__Comamonadaceae;g__Delftia	0.00017	0
d__Bacteria;p__Proteobacteria;c__Gammaproteobacteria;o__Oceanospirillales;f__Halomonadaceae;g__Marinospirillum	0.00017	0

Supplementary Table 1. Subgingival microbiome of gingival inflammation at the phylum, genus, and species levels.

Supplementary Table 1c. Species level.

Taxa	Baseline	6 weeks
d__Bacteria;p__Fusobacteriota;c__Fusobacteriia;o__Fusobacteriales;f__Fusobacteriaceae;g__Fusobacterium;__	8.882	10.09518
d__Bacteria;p__Fusobacteriota;c__Fusobacteriia;o__Fusobacteriales;f__Fusobacteriaceae;g__Fusobacterium;s__Fusobacterium_nucleatum	7.51267	5.01857
d__Bacteria;p__Actinobacteriota;c__Actinobacteria;o__Corynebacteriales;f__Corynebacteriaceae;g__Corynebacterium;s__Corynebacterium_matruchotii	2.072	2.81554
d__Bacteria;p__Firmicutes;c__Negativicutes;o__Veillonellales-Selenomonadales;f__Veillonellaceae;g__Veillonella;__	2.81233	2.55589
d__Bacteria;p__Firmicutes;c__Bacilli;o__Lactobacillales;f__Streptococcaceae;g__Streptococcus;__	1.8455	2.43964
d__Bacteria;p__Campilobacterota;c__Campylobacteria;o__Campylobacterales;f__Campylobacteraceae;g__Campylobacter;s__Campylobacter_gracilis	2.19383	2.14714
d__Bacteria;p__Firmicutes;c__Bacilli;o__Lactobacillales;f__Carnobacteriaceae;g__Alkalibacterium;__	1.2725	2.11054
d__Bacteria;p__Actinobacteriota;c__Actinobacteria;o__Micrococcales;f__Micrococcaceae;g__Rothia;__	2.2555	2.00893
d__Bacteria;p__Bacteroidota;c__Bacteroidia;o__Bacteroidales;f__Porphyromonadaceae;g__Porphyromonas;s__Porphyromonas_gingivalis	1.383	1.79875
d__Bacteria;p__Bacteroidota;c__Bacteroidia;o__Flavobacteriales;f__Flavobacteriaceae;g__Capnocytophaga;s__Capnocytophaga_ochracea	1.2345	1.77536
d__Bacteria;p__Actinobacteriota;c__Actinobacteria;o__Actinomycetales;f__Actinomycetaceae;g__Actinomyces;__	1.9115	1.76286
d__Bacteria;p__Firmicutes;c__Bacilli;o__Lactobacillales;f__Enterococcaceae;g__Enterococcus;__	1.14483	1.72839
d__Bacteria;p__Spirochaetota;c__Spirochaetia;o__Spirochaetales;f__Spirochaetaceae;g__Treponema;s__Treponema_medium	1.44917	1.43768
d__Bacteria;p__Bacteroidota;c__Bacteroidia;o__Bacteroidales;f__Prevotellaceae;g__Prevotella;s__Prevotella_intermedia	1.23	1.34411
d__Bacteria;p__Bacteroidota;c__Bacteroidia;o__Bacteroidales;f__Porphyromonadaceae;g__Porphyromonas;s__Porphyromonas_endodontalis	1.34533	1.32661
d__Bacteria;p__Bacteroidota;c__Bacteroidia;o__Bacteroidales;f__Paludibacteraceae;g__F0058;s__uncultured_bacterium	1.93767	1.27286
d__Bacteria;p__Fusobacteriota;c__Fusobacteriia;o__Fusobacteriales;f__Leptotrichiaceae;g__Leptotrichia;__	0.96383	1.23357
d__Bacteria;p__Proteobacteria;c__Gammaproteobacteria;o__Pasteurellales;f__Pasteurellaceae;g__Haemophilus;s__Haemophilus_parainfluenzae	1.31767	1.16571
d__Bacteria;p__Firmicutes;c__Bacilli;o__Lactobacillales;f__Streptococcaceae;g__Streptococcus;s__Streptococcus_sanguinis	0.82333	1.09339
d__Bacteria;p__Proteobacteria;c__Gammaproteobacteria;o__Burkholderiales;f__Neisseriaceae;g__Neisseria;__	0.99167	1.08982
d__Bacteria;p__Proteobacteria;c__Gammaproteobacteria;o__Burkholderiales;f__Neisseriaceae;g__Neisseria;s__Neisseria_oralis	0.605	1.06518
d__Bacteria;p__Bacteroidota;c__Bacteroidia;o__Flavobacteriales;f__Flavobacteriaceae;g__Capnocytophaga;s__Capnocytophaga_gingivalis	1.25033	0.99214
d__Bacteria;p__Proteobacteria;c__Gammaproteobacteria;o__Cardiobacteriales;f__Cardiobacteriaceae;g__Cardiobacterium;s__uncultured_bacterium	1.03767	0.97
d__Bacteria;p__Bacteroidota;c__Bacteroidia;o__Bacteroidales;f__Tannerellaceae;g__Tannerella;s__Tannerella_forsythia	1.01967	0.95411
d__Bacteria;p__Proteobacteria;c__Gammaproteobacteria;o__Burkholderiales;f__Neisseriaceae;g__Kingella;s__uncultured_bacterium	0.70017	0.94786
d__Bacteria;p__Spirochaetota;c__Spirochaetia;o__Spirochaetales;f__Spirochaetaceae;g__Treponema;s__Treponema_socranskii	1.08033	0.94518
d__Bacteria;p__Bacteroidota;c__Bacteroidia;o__Bacteroidales;f__Prevotellaceae;g__Prevotella;s__Prevotella_nigrescens	1.33667	0.92125
d__Bacteria;p__Bacteroidota;c__Bacteroidia;o__Flavobacteriales;f__Flavobacteriaceae;g__Capnocytophaga;__	0.64117	0.88
d__Bacteria;p__Bacteroidota;c__Bacteroidia;o__Sphingobacteriales;f__Lentimicrobiaceae;g__Lentimicrobium;s__Bacteroidales_genomosp.	0.80617	0.835
d__Bacteria;p__Bacteroidota;c__Bacteroidia;o__Bacteroidales;f__Prevotellaceae;g__Prevotella;s__Prevotella_oris	0.70317	0.82339

d__Bacteria;p__Bacteroidota;c__Bacteroidia;o__Flavobacteriales;f__Flavobacteriaceae;g__Capnocytophaga;s__Capnocytophaga_sp.	0.93167	0.81179
d__Bacteria;p__Bacteroidota;c__Bacteroidia;o__Bacteroidales;f__Porphyromonadaceae;g__Porphyromonas;s__uncultured_bacterium	0.45333	0.80143
d__Bacteria;p__Bacteroidota;c__Bacteroidia;o__Bacteroidales;f__Prevotellaceae;g__Alloprevotella;s__Alloprevotella_tannerae	1.21817	0.78929
d__Bacteria;p__Spirochaetota;c__Spirochaetia;o__Spirochaetales;f__Spirochaetaceae;g__Treponema;s__Treponema_denticola	1.051	0.73661
d__Bacteria;p__Synergistota;c__Synergistia;o__Synergistales;f__Synergistaceae;g__Fretibacterium;__	0.71583	0.72536
d__Bacteria;p__Campilobacterota;c__Campylobacteria;o__Campylobacterales;f__Campylobacteraceae;g__Campylobacter;__	0.73283	0.70964
d__Bacteria;p__Firmicutes;c__Bacilli;o__Exiguobacterales;f__Exiguobacteraceae;g__Exiguobacterium;__	0.50167	0.68518
d__Bacteria;p__Bacteroidota;c__Bacteroidia;o__Flavobacteriales;f__Flavobacteriaceae;g__Capnocytophaga;s__Capnocytophaga_sputigena	0.7595	0.67214
d__Bacteria;p__Bacteroidota;c__Bacteroidia;o__Bacteroidales;f__Prevotellaceae;g__Prevotella;s__Prevotella_loescheii	0.88	0.6675
d__Bacteria;p__Fusobacteriota;c__Fusobacteriia;o__Fusobacteriales;f__Leptotrichiaceae;g__Leptotrichia;s__Leptotrichia_goodfellowii	0.64133	0.61821
d__Bacteria;p__Spirochaetota;c__Spirochaetia;o__Spirochaetales;f__Spirochaetaceae;g__Treponema;__	0.55517	0.615
d__Bacteria;p__Fusobacteriota;c__Fusobacteriia;o__Fusobacteriales;f__Leptotrichiaceae;g__Leptotrichia;s__Leptotrichia_hofstadii	0.33017	0.60375
d__Bacteria;p__Firmicutes;c__Clostridia;o__Peptostreptococcales-Tissierellales;f__Peptostreptococcaceae;g__Filifactor;s__Filifactor_alocis	0.58283	0.58518
d__Bacteria;p__Firmicutes;c__Bacilli;o__Bacillales;f__Bacillaceae;g__Anaerobacillus;__	0.411	0.58161
d__Bacteria;p__Actinobacteriota;c__Actinobacteria;o__Actinomycetales;f__Actinomycetaceae;g__Actinomyces;s__Actinomyces_gerencseriae	0.40767	0.57446
d__Bacteria;p__Bacteroidota;c__Bacteroidia;o__Bacteroidales;f__Porphyromonadaceae;g__Porphyromonas;s__Porphyromonas_catoniae	0.67533	0.55607
d__Bacteria;p__Proteobacteria;c__Gammaproteobacteria;o__Pasteurellales;f__Pasteurellaceae;g__Aggregatibacter;s__uncultured_bacterium	0.61533	0.52071
d__Bacteria;p__Bacteroidota;c__Bacteroidia;o__Bacteroidales;f__Prevotellaceae;g__Prevotella;s__Prevotella_shahii	0.85383	0.50679
d__Bacteria;p__Actinobacteriota;c__Actinobacteria;o__Actinomycetales;f__Actinomycetaceae;g__F0332;s__uncultured_bacterium	0.57967	0.50339
d__Bacteria;p__Firmicutes;c__Negativicutes;o__Veillonellales-Selenomonadales;f__Selenomonadaceae;g__Selenomonas;s__Selenomonas_noxia	0.473	0.49536
d__Bacteria;p__Firmicutes;c__Clostridia;o__Clostridia_UCG-014;f__Clostridia_UCG-014;g__Clostridia_UCG-014;__	0.08433	0.48071
d__Bacteria;p__Firmicutes;c__Bacilli;o__Lactobacillales;f__Enterococcaceae;g__Enterococcus;s__Enterococcus_italicus	0.23317	0.47518
d__Bacteria;p__Proteobacteria;c__Gammaproteobacteria;o__Burkholderiales;f__Neisseriaceae;g__Neisseria;s__Neisseria_bacilliformis	0.3015	0.47286
d__Bacteria;p__Proteobacteria;c__Gammaproteobacteria;o__Pasteurellales;f__Pasteurellaceae;g__Aggregatibacter;s__Aggregatibacter_aphrophilus	0.80183	0.46804
d__Bacteria;p__Bacteroidota;c__Bacteroidia;o__Flavobacteriales;f__Flavobacteriaceae;g__Capnocytophaga;s__Capnocytophaga_leadbetteri	0.42733	0.45446
d__Bacteria;p__Bacteroidota;c__Bacteroidia;o__Bacteroidales;f__Prevotellaceae;g__Prevotella;s__Prevotella_melaninogenica	0.477	0.45179
d__Bacteria;p__Bacteroidota;c__Bacteroidia;o__Flavobacteriales;f__Weeksellaceae;g__Bergeyella;s__uncultured_bacterium	0.43167	0.44875
d__Bacteria;p__Bacteroidota;c__Bacteroidia;o__Bacteroidales;f__Tannerellaceae;g__Tannerella;s__Tannerella_sp.	0.30183	0.44661
d__Bacteria;p__Bacteroidota;c__Bacteroidia;o__Bacteroidales;f__Prevotellaceae;g__Prevotella;__	0.49067	0.43196
d__Bacteria;p__Firmicutes;c__Negativicutes;o__Veillonellales-Selenomonadales;f__Selenomonadaceae;g__Selenomonas;__	0.22717	0.43179
d__Bacteria;p__Patescibacteria;c__Saccharimonadia;o__Saccharimonadales;f__Saccharimonadaceae;g__Saccharimonadaceae;s__uncultured_Candidatus	0.52967	0.42446
d__Bacteria;p__Fusobacteriota;c__Fusobacteriia;o__Fusobacteriales;f__Leptotrichiaceae;g__Leptotrichia;s__Leptotrichia_buccalis	0.5215	0.41286
d__Bacteria;p__Proteobacteria;c__Gammaproteobacteria;o__Cardiobacteriales;f__Cardiobacteriaceae;g__Cardiobacterium;s__Cardiobacterium_valvarum	0.42833	0.40464
d__Bacteria;p__Actinobacteriota;c__Actinobacteria;o__Micrococcales;f__Micrococcaceae;g__Rothia;s__Rothia_aeria	0.52133	0.40446
d__Bacteria;p__Bacteroidota;c__Bacteroidia;o__Flavobacteriales;f__Flavobacteriaceae;g__Capnocytophaga;s__Capnocytophaga_granulosa	0.41883	0.40339
d__Bacteria;p__Firmicutes;c__Negativicutes;o__Veillonellales-Selenomonadales;f__Veillonellaceae;g__Dialister;s__Dialister_invisus	0.71817	0.39464

d__Bacteria;p__Patescibacteria;c__Saccharimonadia;o__Saccharimonadales;f__Saccharimonadaceae;g__Saccharimonadaceae;s__uncultured_bacterium	0.52683	0.38982
d__Bacteria;p__Proteobacteria;c__Gammaproteobacteria;o__Burkholderiales;f__Neisseriaceae;g__Eikenella;__	0.35417	0.38071
d__Bacteria;p__Proteobacteria;c__Gammaproteobacteria;o__Burkholderiales;f__Burkholderiaceae;g__Lautropia;__	0.61483	0.37125
d__Bacteria;p__Bacteroidota;c__Bacteroidia;o__Bacteroidales;f__Porphyromonadaceae;g__Porphyromonas;__	0.4	0.36482
d__Bacteria;p__Proteobacteria;c__Gammaproteobacteria;o__Pasteurellales;f__Pasteurellaceae;g__Haemophilus;__	0.44383	0.35929
d__Bacteria;p__Bacteroidota;c__Bacteroidia;o__Bacteroidales;f__Prevotellaceae;g__Prevotella;s__Prevotella_sp.	0.23383	0.35893
d__Bacteria;p__Proteobacteria;c__Gammaproteobacteria;o__Pasteurellales;f__Pasteurellaceae;g__Aggregatibacter;__	0.20983	0.3575
d__Bacteria;p__Patescibacteria;c__Saccharimonadia;o__Saccharimonadales;f__Saccharimonadaceae;g__Saccharimonadaceae;__	0.24883	0.35429
d__Bacteria;p__Bacteroidota;c__Bacteroidia;o__Bacteroidales;f__Prevotellaceae;g__Prevotella;s__Prevotella_pleuritidis	0.45617	0.35357
d__Bacteria;p__Bacteroidota;c__Bacteroidia;o__Bacteroidales;f__Tannerellaceae;g__Tannerella;__	0.16733	0.35089
d__Bacteria;p__Fusobacteriota;c__Fusobacteriia;o__Fusobacteriales;f__Leptotrichiaceae;g__Leptotrichia;s__uncultured_bacterium	0.2305	0.3425
d__Bacteria;p__Firmicutes;c__Negativicutes;o__Veillonellales-Selenomonadales;f__Veillonellaceae;g__Veillonella;s__Veillonella_parvula	0.20983	0.33929
d__Bacteria;p__Proteobacteria;c__Alphaproteobacteria;o__Rhodobacterales;f__Rhodobacteraceae;g__Ketogulonicigenium;s__Ketogulonicigenium_vulgare	0.19033	0.33554
d__Bacteria;p__Actinobacteriota;c__Actinobacteria;o__Corynebacteriales;f__Corynebacteriaceae;g__Corynebacterium;s__Corynebacterium_durum	0.3655	0.33304
d__Bacteria;p__Fusobacteriota;c__Fusobacteriia;o__Fusobacteriales;f__Leptotrichiaceae;g__Leptotrichia;s__Leptotrichia_wadei	0.20017	0.30714
d__Bacteria;p__Fusobacteriota;c__Fusobacteriia;o__Fusobacteriales;f__Leptotrichiaceae;g__Leptotrichia;s__Leptotrichia_sp.	0.24183	0.30089
d__Bacteria;p__Firmicutes;c__Bacilli;o__Bacillales;f__Salisediminibacteriaceae;g__Salipaludibacillus;__	0.2695	0.30071
d__Bacteria;p__Bacteroidota;c__Bacteroidia;o__Bacteroidales;f__Prevotellaceae;g__Prevotella;s__unidentified_eubacterium	0.60783	0.29393
d__Bacteria;p__Firmicutes;c__Clostridia;o__Lachnospirales;f__Lachnospiraceae;g__Lachnoanaerobaculum;s__uncultured_bacterium	0.2435	0.28946
d__Bacteria;p__Firmicutes;c__Clostridia;o__Peptostreptococcales-Tissierellales;f__Peptostreptococcaceae;g__[Eubacterium]_yurii_group;__	0.30117	0.28911
d__Bacteria;p__Fusobacteriota;c__Fusobacteriia;o__Fusobacteriales;f__Fusobacteriaceae;g__Fusobacterium;s__Fusobacterium_periodonticum	0.07867	0.28732
d__Bacteria;p__Spirochaetota;c__Spirochaetia;o__Spirochaetales;f__Spirochaetaceae;g__Treponema;s__uncultured_bacterium	0.17567	0.28643
d__Bacteria;p__Bacteroidota;c__Bacteroidia;o__Flavobacteriales;f__Flavobacteriaceae;g__Capnocytophaga;s__Capnocytophaga_haemolytica	0.1605	0.28375
d__Bacteria;p__Firmicutes;c__Clostridia;o__Peptostreptococcales-Tissierellales;f__Peptostreptococcales-Tissierellales;g__Parvimonas;__	0.40567	0.2725
d__Bacteria;p__Actinobacteriota;c__Actinobacteria;o__Micrococcales;f__Cellulomonadaceae;g__Actinotalea;__	0.11333	0.27054
d__Bacteria;p__Firmicutes;c__Clostridia;o__Lachnospirales;f__Lachnospiraceae;g__Johnsonella;s__Johnsonella_ignava	0.17067	0.26607
d__Bacteria;p__Spirochaetota;c__Spirochaetia;o__Spirochaetales;f__Spirochaetaceae;g__Treponema;s__Treponema_genomosp.	0.14717	0.26571
d__Bacteria;p__Firmicutes;c__Negativicutes;o__Veillonellales-Selenomonadales;f__Selenomonadaceae;g__Selenomonas;s__Selenomonas_sputigena	0.45833	0.25714
d__Bacteria;p__Firmicutes;c__Clostridia;o__Lachnospirales;f__Lachnospiraceae;g__Catonella;__	0.26017	0.25661
d__Bacteria;p__Bacteroidota;c__Bacteroidia;o__Bacteroidales;f__Prevotellaceae;g__Prevotella;s__Prevotella_denticola	1.04367	0.25643
d__Bacteria;p__Firmicutes;c__Bacilli;o__Lactobacillales;f__Carnobacteriaceae;g__Granulicatella;__	0.12167	0.245
d__Bacteria;p__Firmicutes;c__Bacilli;o__Mycoplasmatales;f__Mycoplasmataceae;g__Mycoplasma;__	0.22933	0.24143
d__Bacteria;p__Bacteroidota;c__Bacteroidia;o__Bacteroidales;f__Prevotellaceae;g__Prevotella;s__Prevotella_oulorum	0.20467	0.23732
d__Bacteria;p__Patescibacteria;c__Saccharimonadia;o__Saccharimonadales;f__Saccharimonadales;g__Saccharimonadales;s__Candidatus_Saccharibacteria	0.25433	0.23679
d__Bacteria;p__Fusobacteriota;c__Fusobacteriia;o__Fusobacteriales;f__Leptotrichiaceae;g__Leptotrichia;s__Leptotrichia_trevisanii	0.26817	0.22196
d__Bacteria;p__Patescibacteria;c__Saccharimonadia;o__Saccharimonadales;f__Saccharimonadaceae;g__TM7x;__	0.217	0.21286

d__Bacteria;p__Firmicutes;c__Bacilli;o__Lactobacillales;f__Streptococcaceae;g__Streptococcus;s__Streptococcus_gordonii	0.073	0.21036
d__Bacteria;p__Firmicutes;c__Clostridia;o__Lachnospirales;f__Lachnospiraceae;g__Oribacterium;__	0.224	0.20964
d__Bacteria;p__Proteobacteria;c__Gammaproteobacteria;o__Burkholderiales;f__Neisseriaceae;g__Eikenella;s__uncultured_bacterium	0.18517	0.20839
d__Bacteria;p__Actinobacteriota;c__Actinobacteria;o__Actinomycetales;f__Actinomycetaceae;g__Actinomyces;s__Actinomyces_massiliensis	0.28433	0.2075
d__Bacteria;p__Spirochaetota;c__Spirochaetia;o__Spirochaetales;f__Spirochaetaceae;g__Treponema;s__Treponema_maltophilum	0.33617	0.20304
d__Bacteria;p__Spirochaetota;c__Spirochaetia;o__Spirochaetales;f__Spirochaetaceae;g__Treponema;s__Treponema_sp.	0.18583	0.20125
d__Bacteria;p__Proteobacteria;c__Alphaproteobacteria;o__Rhodobacterales;f__Rhodobacteraceae;g__Paracoccus;__	0.07967	0.20054
d__Bacteria;p__Spirochaetota;c__Spirochaetia;o__Spirochaetales;f__Spirochaetaceae;g__Treponema;s__Treponema_lecithinolyticum	0.24733	0.19732
d__Bacteria;p__Firmicutes;c__Bacilli;o__Mycoplasmatales;f__Mycoplasmataceae;g__Mycoplasma;s__Metamycoplasma_salivarium	0.0795	0.19036
d__Bacteria;p__Actinobacteriota;c__Actinobacteria;o__Propionibacteriales;f__Propionibacteriaceae;g__Pseudopropionibacterium;s__uncultured_bacterium	0.13433	0.18357
d__Bacteria;p__Bacteroidota;c__Bacteroidia;o__Bacteroidales;f__Paludibacteraceae;g__Paludibacter;s__uncultured_bacterium	0.11283	0.18161
d__Bacteria;p__Firmicutes;c__Clostridia;o__Lachnospirales;f__Lachnospiraceae;g__Johnsonella;s__uncultured_bacterium	0.185	0.18143
d__Bacteria;p__Firmicutes;c__Clostridia;o__Lachnospirales;f__Defluviitaleaceae;g__Defluviitaleaceae_UCG-011;s__Lachnospiraceae_bacterium	0.16983	0.18036
d__Bacteria;p__Firmicutes;c__Bacilli;o__Staphylococcales;f__Gemellaceae;g__Gemella;__	0.1055	0.18
d__Bacteria;p__Bacteroidota;c__Bacteroidia;o__Bacteroidales;f__Prevotellaceae;g__Prevotella;s__Prevotella_salivae	0.09967	0.17964
d__Bacteria;p__Bacteroidota;c__Bacteroidia;o__Bacteroidales;f__Prevotellaceae;g__Prevotella;s__Prevotella_saccharolytica	0.17433	0.1775
d__Bacteria;p__Actinobacteriota;c__Coriobacteriia;o__Coriobacteriales;f__Atopobiaceae;g__Olsenella;s__Olsenella_sp.	0.11383	0.17071
d__Bacteria;p__Bacteroidota;c__Bacteroidia;o__Bacteroidales;f__Prevotellaceae;g__Prevotella;s__Prevotella_genomosp.	0.404	0.17036
d__Bacteria;p__Firmicutes;c__Negativicutes;o__Veillonellales-Selenomonadales;f__Selenomonadaceae;__;__	0.19433	0.16679
d__Bacteria;p__Desulfobacterota;c__Desulfobulbia;o__Desulfobulbales;f__Desulfobulbaceae;g__Desulfobulbus;__	0.33067	0.16375
d__Bacteria;p__Proteobacteria;c__Gammaproteobacteria;o__Burkholderiales;f__Neisseriaceae;__;__	0.017	0.16321
d__Bacteria;p__Spirochaetota;c__Spirochaetia;o__Spirochaetales;f__Spirochaetaceae;g__Treponema;s__Treponema_vincentii	0.13583	0.16161
d__Bacteria;p__Fusobacteriota;c__Fusobacteriia;o__Fusobacteriales;f__Leptotrichiaceae;g__Leptotrichia;s__uncultured_Leptotrichia	0.2665	0.16
d__Bacteria;p__Patescibacteria;c__Saccharimonadia;o__Saccharimonadales;f__Saccharimonadaceae;g__TM7x;s__uncultured_bacterium	0.08433	0.1575
d__Bacteria;p__Bacteroidota;c__Bacteroidia;o__Bacteroidales;f__Prevotellaceae;g__Prevotella;s__Prevotella_maculosa	0.16167	0.15446
d__Bacteria;p__Campilobacterota;c__Campylobacteria;o__Campylobacterales;f__Campylobacteraceae;g__Campylobacter;s__Campylobacter_concisus	0.14433	0.14893
d__Bacteria;p__Firmicutes;c__Clostridia;o__Peptostreptococcales-Tissierellales;f__Anaerovoracaceae;g__[Eubacterium]_brachy_group;s__[Eubacterium]_brachy	0.1635	0.14589
d__Bacteria;p__Proteobacteria;c__Gammaproteobacteria;o__Burkholderiales;f__Burkholderiaceae;g__Lautropia;s__uncultured_bacterium	0.19133	0.14268
d__Bacteria;p__Proteobacteria;c__Gammaproteobacteria;o__Alteromonadales;f__Alteromonadaceae;g__Alishewanella;__	0.077	0.14196
d__Bacteria;p__Firmicutes;c__Clostridia;o__Lachnospirales;f__Lachnospiraceae;g__Johnsonella;__	0.11333	0.14125
d__Bacteria;p__Bacteroidota;c__Bacteroidia;o__Bacteroidales;f__Tannerellaceae;g__Parabacteroides;s__Parabacteroides_sp.	0.01617	0.13893
d__Bacteria;p__Actinobacteriota;c__Actinobacteria;o__Actinomycetales;f__Actinomycetaceae;g__Actinomyces;s__unidentified	0.0095	0.13875
d__Bacteria;p__Firmicutes;c__Negativicutes;o__Veillonellales-Selenomonadales;f__Veillonellaceae;g__Dialister;s__Dialister_pneumosintes	0.1585	0.13661
d__Bacteria;p__Firmicutes;c__Clostridia;o__Lachnospirales;f__Lachnospiraceae;g__Anaerocolumna;s__uncultured_bacterium	0.062	0.135
d__Bacteria;p__Firmicutes;c__Negativicutes;o__Veillonellales-Selenomonadales;f__Selenomonadaceae;g__Selenomonas;s__Selenomonas_artemidis	0.085	0.13429
d__Bacteria;p__Bacteroidota;c__Bacteroidia;o__Bacteroidales;f__Prevotellaceae;g__Prevotella;s__Prevotella_veroralis	0.13617	0.12964

d__Bacteria;p__Proteobacteria;c__Gammaproteobacteria;o__Alteromonadales;f__Alteromonadaceae;g__Alishewanella;s__uncultured_Rheinheimera	0.08717	0.12929
d__Bacteria;p__Actinobacteriota;c__Actinobacteria;o__Actinomycetales;f__Actinomycetaceae;g__F0332;s__unidentified	0.09767	0.1275
d__Bacteria;p__Actinobacteriota;c__Actinobacteria;o__Actinomycetales;f__Actinomycetaceae;g__Actinomyces;s__uncultured_Actinomyces	0.13267	0.12375
d__Bacteria;p__Firmicutes;c__Negativicutes;o__Veillonellales-Selenomonadales;f__Selenomonadaceae;g__Selenomonas;s__Veillonellaceae_bacterium	0.16467	0.12054
d__Bacteria;p__Firmicutes;c__Negativicutes;o__Veillonellales-Selenomonadales;f__Selenomonadaceae;g__Selenomonas;s__unidentified	0.05783	0.11821
d__Bacteria;p__Bacteroidota;c__Bacteroidia;o__Bacteroidales;f__Prevotellaceae;g__Prevotella;s__Prevotella_oralis	0.09083	0.11661
d__Bacteria;p__Patescibacteria;c__Gracilibacteria;o__Absconditabacteriales_(SR1);f__Absconditabacteriales_(SR1);g__Absconditabacteriales_(SR1);s__SR1_bacterium	0.28233	0.10804
d__Bacteria;p__Firmicutes;c__Negativicutes;o__Veillonellales-Selenomonadales;f__Selenomonadaceae;g__Centipeda;__	0.11467	0.10679
d__Bacteria;p__Bacteroidota;c__Bacteroidia;o__Bacteroidales;f__uncultured;g__uncultured;s__uncultured_bacterium	0.01317	0.10643
d__Bacteria;p__Proteobacteria;c__Gammaproteobacteria;o__Burkholderiales;f__Neisseriaceae;g__Neisseria;s__Neisseria_elongata	0.17433	0.10589
d__Bacteria;p__Patescibacteria;c__Saccharimonadia;o__Saccharimonadales;f__Saccharimonadaceae;g__Candidatus_Saccharimonas;s__uncultured_bacterium	0.18067	0.09929
d__Bacteria;p__Patescibacteria;c__Gracilibacteria;o__JGI_0000069-P22;f__JGI_0000069-P22;g__JGI_0000069-P22;__	0.008	0.09911
d__Bacteria;p__Firmicutes;c__Negativicutes;o__Veillonellales-Selenomonadales;f__Selenomonadaceae;g__Selenomonas;s__Schwartzia_sp.	0.11033	0.09875
d__Bacteria;p__Bacteroidota;c__Bacteroidia;o__Bacteroidales;f__Porphyromonadaceae;g__Porphyromonas;s__Porphyromonas_pasteri	0.04983	0.09875
d__Bacteria;p__Bacteroidota;c__Bacteroidia;o__Bacteroidales;f__Prevotellaceae;g__Alloprevotella;__	0.175	0.09643
d__Bacteria;p__Firmicutes;c__Bacilli;o__Bacillales;f__Bacillaceae;g__Anaerobacillus;s__uncultured_bacterium	0.0125	0.095
d__Bacteria;p__Firmicutes;c__Clostridia;o__Lachnospirales;f__Lachnospiraceae;g__Lachnoanaerobaculum;__	0.09433	0.09393
d__Bacteria;p__Verrucomicrobiota;c__Verrucomicrobiae;o__Opitutales;f__Opitutaceae;g__IMCC26134;__	0.04567	0.09339
d__Bacteria;p__Bacteroidota;c__Bacteroidia;o__Flavobacteriales;f__Weeksellaceae;g__Bergeyella;s__uncultured_Bergeyella	0.149	0.09286
d__Bacteria;p__Firmicutes;c__Clostridia;o__Lachnospirales;f__Defluviitaleaceae;g__Defluviitalea;s__Lachnospiraceae_bacterium	0.06	0.09161
d__Bacteria;p__Fusobacteriota;c__Fusobacteriia;o__Fusobacteriales;f__Leptotrichiaceae;g__uncultured;s__Leptotrichia-like_sp.	0.2065	0.09107
d__Bacteria;p__Firmicutes;c__Bacilli;o__Lactobacillales;f__Lactobacillaceae;g__Lactobacillus;s__Lactobacillus_fermentum	0.064	0.08661
d__Bacteria;p__Firmicutes;c__Negativicutes;o__Veillonellales-Selenomonadales;f__Veillonellaceae;g__Anaeroglobus;s__Anaeroglobus_geminatus	0.05083	0.08607
d__Bacteria;p__Campilobacterota;c__Campylobacteria;o__Campylobacterales;f__Campylobacteraceae;g__Campylobacter;s__Campylobacter_rectus	0.0755	0.08571
d__Bacteria;p__Proteobacteria;c__Gammaproteobacteria;o__Burkholderiales;f__Rhodocyclaceae;g__Propionivibrio;s__Propionibacter_sp.	0.106	0.08411
d__Bacteria;p__Firmicutes;c__Bacilli;o__Lactobacillales;f__Aerococcaceae;g__Abiotrophia;s__uncultured_bacterium	0.09717	0.08393
d__Bacteria;p__Firmicutes;c__Bacilli;o__Paenibacillales;f__Paenibacillaceae;g__Paenibacillus;s__Paenibacillus_borealis	0.01933	0.07964
d__Bacteria;p__Firmicutes;c__Bacilli;o__Lactobacillales;f__Streptococcaceae;g__Streptococcus;s__Streptococcus_mutans	0.41733	0.07732
d__Bacteria;p__Proteobacteria;c__Gammaproteobacteria;o__Burkholderiales;f__Neisseriaceae;g__Neisseria;s__uncultured_bacterium	0.03117	0.07589
d__Bacteria;p__Bacteroidota;c__Bacteroidia;o__Bacteroidales;f__Bacteroidales_Incertae_Sedis;g__Phocaeicola;s__Phocaeicola_abscessus	0.1375	0.07357
d__Bacteria;p__Firmicutes;c__Bacilli;o__Acholeplasmatales;f__Acholeplasmataceae;g__Acholeplasma;__	0.03417	0.07339
d__Bacteria;p__Firmicutes;c__Bacilli;o__Staphylococcales;f__Gemellaceae;g__Gemella;s__uncultured_organism	0.0915	0.07268
d__Bacteria;p__Firmicutes;c__Clostridia;o__Clostridia_UCG-014;f__Clostridia_UCG-014;g__Clostridia_UCG-014;s__Clostridiales_bacterium	0.125	0.07196
d__Bacteria;p__Proteobacteria;c__Gammaproteobacteria;o__Burkholderiales;f__Neisseriaceae;g__Neisseria;s__uncultured_Kingella	0.017	0.07196
d__Bacteria;p__Firmicutes;c__Negativicutes;o__Veillonellales-Selenomonadales;f__Veillonellaceae;g__Veillonella;s__Veillonella_atypica	0.041	0.06804
d__Bacteria;p__Firmicutes;c__Bacilli;o__Lactobacillales;f__Streptococcaceae;g__Lactococcus;s__Lactococcus_lactis	0.03933	0.065

d__Bacteria;p__Proteobacteria;c__Gammaproteobacteria;o__Burkholderiales;f__Comamonadaceae;g__Comamonas;s__Ottowia_sp.	0.093	0.06411
d__Bacteria;p__Bacteroidota;c__Bacteroidia;o__Bacteroidales;f__Rikenellaceae;g__Rikenellaceae_RC9_gut_group;s__Bacteroidales_oral	0.039	0.06411
d__Bacteria;p__Firmicutes;c__Clostridia;o__Peptostreptococcales-Tissierellales;f__Anaerovoracaceae;g__[Eubacterium]_saphenum_group;s__Eubacterium_saphenum	0.05683	0.06321
d__Bacteria;p__Bacteroidota;c__Bacteroidia;o__Bacteroidales;f__Prevotellaceae;g__Prevotella;s__Prevotella_micans	0.064	0.06143
d__Bacteria;p__Firmicutes;c__Bacilli;o__Erysipelotrichales;f__Erysipelotrichaceae;g__Erysipelothrix;s__uncultured_bacterium	0.03483	0.06107
d__Bacteria;p__Bacteroidota;c__Bacteroidia;o__Flavobacteriales;f__Flavobacteriaceae;g__Flavobacterium;s__Flavobacterium_luticocti	0.02917	0.06107
d__Bacteria;p__Firmicutes;c__Clostridia;o__Peptostreptococcales-Tissierellales;f__Peptostreptococcales-Tissierellales;g__uncultured;s__uncultured_Peptostreptococcus	0.02367	0.05982
d__Bacteria;p__Bacteroidota;c__Bacteroidia;o__Flavobacteriales;f__Flavobacteriaceae;g__Capnocytophaga;s__uncultured_Capnocytophaga	0.25317	0.05857
d__Bacteria;p__Bacteroidota;c__Bacteroidia;o__Bacteroidales;f__Prevotellaceae;g__Prevotella;s__Prevotella_dentalis	0.074	0.05804
d__Bacteria;p__Firmicutes;c__Bacilli;o__Lactobacillales;f__Streptococcaceae;g__Streptococcus;s__Streptococcus_salivarius	0.01583	0.0575
d__Bacteria;p__Proteobacteria;c__Gammaproteobacteria;o__Cardiobacteriales;f__Cardiobacteriaceae;g__Cardiobacterium;s__uncultured_Cardiobacterium	0.00217	0.05732
d__Bacteria;p__Firmicutes;c__Bacilli;o__Lactobacillales;f__uncultured;g__uncultured;s__uncultured_bacterium	0.016	0.05714
d__Bacteria;p__Patescibacteria;c__Gracilibacteria;o__JGI_0000069-P22;f__JGI_0000069-P22;g__JGI_0000069-P22;s__Gracilibacteria_bacterium	0.04883	0.05661
d__Bacteria;p__Bacteroidota;c__Bacteroidia;o__Bacteroidales;f__Rikenellaceae;g__Rikenellaceae_RC9_gut_group;s__Porphyromonas-like_sp.	0.07983	0.05643
d__Bacteria;p__Firmicutes;c__Clostridia;o__Peptostreptococcales-Tissierellales;f__Peptostreptococcaceae;g__Acetoanaerobium;s__uncultured_bacterium	0.01317	0.05429
d__Bacteria;p__Bacteroidota;c__Bacteroidia;o__Bacteroidales;f__Tannerellaceae;g__Tannerella;s__uncultured_bacterium	0.10083	0.05375
d__Bacteria;p__Firmicutes;c__Clostridia;o__Peptostreptococcales-Tissierellales;f__Peptostreptococcales-Tissierellales;__;__	0.0405	0.05321
d__Bacteria;p__Bacteroidota;c__Bacteroidia;o__Bacteroidales;f__Dysgonomonadaceae;g__Dysgonomonas;s__uncultured_Bacteroidetes	0.02483	0.0525
d__Bacteria;p__Proteobacteria;c__Gammaproteobacteria;o__Pseudomonadales;f__Pseudomonadaceae;g__Pseudomonas;s__Pseudomonas_xinjiangensis	0.034	0.05196
d__Bacteria;p__Desulfobacterota;c__Desulfovibrionia;o__Desulfovibrionales;f__Desulfoplanaceae;g__Desulfoplanes;__	0	0.05196
d__Bacteria;p__Bacteroidota;c__Bacteroidia;o__Bacteroidales;f__Rikenellaceae;g__Rikenellaceae_RC9_gut_group;s__uncultured_eubacterium	0.08217	0.05
d__Bacteria;p__Bacteroidota;c__Bacteroidia;o__Bacteroidales;f__Prevotellaceae;__;__	0.02483	0.05
d__Bacteria;p__Firmicutes;c__Clostridia;o__Peptostreptococcales-Tissierellales;f__Peptostreptococcaceae;g__Peptostreptococcus;__	0.09533	0.04911
d__Bacteria;p__Firmicutes;c__Clostridia;o__Lachnospirales;f__Lachnospiraceae;g__Catonella;s__Catonella_sp.	0.02083	0.04893
d__Bacteria;p__Chloroflexi;c__Anaerolineae;o__Anaerolineales;f__Anaerolineaceae;g__Flexilinea;s__Anaerolineaceae_bacterium	0.1245	0.04714
d__Bacteria;p__Bacteroidota;c__Bacteroidia;o__Bacteroidales;f__Prevotellaceae;g__Prevotella;s__Prevotella_pallens	0.06133	0.045
d__Bacteria;p__Firmicutes;c__Clostridia;o__Lachnospirales;f__Lachnospiraceae;g__Lachnoclostridium;__	0.02333	0.045
d__Bacteria;p__Firmicutes;c__Clostridia;o__Peptococcales;f__Peptococcaceae;g__Peptococcus;s__Peptococcus_sp.	0.07317	0.04446
d__Bacteria;p__Bacteroidota;c__Bacteroidia;o__Sphingobacteriales;f__Lentimicrobiaceae;g__Lentimicrobium;s__Bacteroidia_bacterium	0.04317	0.04304
d__Bacteria;p__Firmicutes;c__Bacilli;o__Lactobacillales;f__Streptococcaceae;g__Streptococcus;s__Streptococcus_constellatus	0.028	0.04286
d__Bacteria;p__Firmicutes;c__Clostridia;o__Lachnospirales;f__Lachnospiraceae;__;__	0.07583	0.04089
d__Bacteria;p__Spirochaetota;c__Spirochaetia;o__Spirochaetales;f__Spirochaetaceae;g__Treponema;s__unidentified	0.08133	0.04071
d__Bacteria;p__Firmicutes;c__Clostridia;o__Peptostreptococcales-Tissierellales;f__Peptostreptococcaceae;g__Peptoanaerobacter;s__Peptoanaerobacter_stomatis	0.05733	0.04071
d__Bacteria;p__Actinobacteriota;c__Actinobacteria;o__Actinomycetales;f__Actinomycetaceae;g__Actinomyces;s__Schaalia_odontolytica	0.0635	0.04054
d__Bacteria;p__Firmicutes;c__Clostridia;o__Peptostreptococcales-Tissierellales;f__Fusibacteraceae;g__Fusibacter;__	0.01533	0.04036
d__Bacteria;p__Bacteroidota;c__Bacteroidia;o__Bacteroidales;f__Prevotellaceae;g__Prevotella;s__Prevotella_baroniae	0.04667	0.03964

d__Bacteria;p__Firmicutes;c__Clostridia;o__Lachnospirales;f__Lachnospiraceae;g__Roseburia;s__Lachnospiraceae_bacterium	0.02967	0.03911
d__Bacteria;p__Patescibacteria;c__Saccharimonadia;o__Saccharimonadales;f__Saccharimonadaceae;g__TM7x;s__uncultured_Candidatus	0.098	0.03839
d__Bacteria;p__Firmicutes;c__Negativicutes;o__Veillonellales-Selenomonadales;f__Selenomonadaceae;g__Centipeda;s__uncultured_bacterium	0.10933	0.03714
d__Bacteria;p__Bacteroidota;c__Bacteroidia;o__Bacteroidales;f__Prevotellaceae;g__Alloprevotella;s__Alloprevotella_rava	0.04917	0.03696
d__Bacteria;p__Synergistota;c__Synergistia;o__Synergistales;f__Synergistaceae;g__Fretibacterium;s__uncultured_Synergistetes	0.01267	0.03625
d__Bacteria;p__Firmicutes;c__Negativicutes;o__Veillonellales-Selenomonadales;f__Veillonellaceae;g__Megasphaera;s__uncultured_bacterium	0.00083	0.03571
d__Bacteria;p__Firmicutes;c__Bacilli;o__Izemoplasmatales;f__Izemoplasmatales;g__Izemoplasmatales;s__uncultured_bacterium	0.02483	0.03536
d__Bacteria;p__Firmicutes;c__Bacilli;o__Acholeplasmatales;f__Acholeplasmataceae;g__Acholeplasma;s__Mycoplasmataceae_genomosp.	0.2545	0.03446
d__Bacteria;p__Firmicutes;c__Clostridia;o__Peptococcales;f__Peptococcaceae;g__Peptococcus;__	0.029	0.03446
d__Bacteria;p__Firmicutes;c__Clostridia;o__Lachnospirales;f__Lachnospiraceae;g__Lachnoclostridium;s__Clostridium_sp.	0.023	0.03339
d__Bacteria;p__Firmicutes;c__Clostridia;o__Oscillospirales;f__Ethanoligenenaceae;g__Ethanoligenens;s__uncultured_bacterium	0.00333	0.03304
d__Bacteria;p__Patescibacteria;c__Saccharimonadia;o__Saccharimonadales;__;__;__	0.04033	0.03268
d__Bacteria;p__Patescibacteria;c__Saccharimonadia;o__Saccharimonadales;f__Saccharimonadaceae;g__Candidatus_Saccharimonas;s__TM7_phylum	0.0205	0.03179
d__Bacteria;p__Patescibacteria;c__Gracilibacteria;o__Gracilibacteria;f__Gracilibacteria;g__Gracilibacteria;s__Gracilibacteria_bacterium	0.00583	0.03179
d__Bacteria;p__Bacteroidota;c__Bacteroidia;o__Bacteroidales;f__Dysgonomonadaceae;g__Dysgonomonas;s__uncultured_bacterium	0.00817	0.03143
d__Bacteria;p__Firmicutes;c__Clostridia;o__Peptostreptococcales-Tissierellales;f__Anaerovoracaceae;g__uncultured;s__Peptostreptococcaceae_bacterium	0.02467	0.03107
d__Bacteria;p__Patescibacteria;c__Saccharimonadia;o__Saccharimonadales;f__Saccharimonadaceae;g__Candidatus_Saccharimonas;__	0.00883	0.03071
d__Bacteria;p__Firmicutes;c__Clostridia;o__Clostridia_vadinBB60_group;f__Clostridia_vadinBB60_group;g__Clostridia_vadinBB60_group;s__Clostridiales_bacterium	0.03067	0.02946
d__Bacteria;p__Bacteroidota;c__Bacteroidia;o__Bacteroidales;f__Prevotellaceae;g__Prevotella;s__Prevotella_fusca	0.07217	0.02893
d__Bacteria;p__Firmicutes;c__Bacilli;o__Lactobacillales;f__Lactobacillaceae;g__Lactobacillus;__	0.12967	0.02821
d__Bacteria;p__Firmicutes;c__Bacilli;o__Erysipelotrichales;f__Erysipelotrichaceae;g__Solobacterium;s__Solobacterium_moorei	0.01683	0.02821
d__Bacteria;p__Proteobacteria;c__Gammaproteobacteria;o__Pasteurellales;f__Pasteurellaceae;g__Aggregatibacter;s__Aggregatibacter_actinomycetemcomitans	0.12083	0.02804
d__Bacteria;p__Proteobacteria;c__Gammaproteobacteria;o__Burkholderiales;f__Neisseriaceae;g__Kingella;__	0.01467	0.02732
d__Bacteria;p__Bacteroidota;c__Bacteroidia;o__Bacteroidales;f__Marinifilaceae;g__Odoribacter;s__Bacteroidetes_sp.	0.00667	0.02732
d__Bacteria;p__Proteobacteria;c__Gammaproteobacteria;o__Burkholderiales;f__Neisseriaceae;g__Eikenella;s__Eikenella_sp.	0.011	0.02714
d__Bacteria;p__Firmicutes;c__Clostridia;o__Peptostreptococcales-Tissierellales;f__Anaerovoracaceae;g__uncultured;__	0.04667	0.02625
d__Bacteria;p__Firmicutes;c__Clostridia;o__Peptostreptococcales-Tissierellales;f__Peptostreptococcales-Tissierellales;g__Serpentinicella;__	0.0055	0.02589
d__Bacteria;p__Firmicutes;c__Clostridia;o__Lachnospirales;f__Lachnospiraceae;g__uncultured;s__uncultured_Roseburia	0.022	0.02554
d__Bacteria;p__Bacteroidota;c__Bacteroidia;o__Flavobacteriales;f__Flavobacteriaceae;g__Flavobacterium;__	0.01217	0.02536
d__Bacteria;p__Firmicutes;c__Clostridia;o__Peptostreptococcales-Tissierellales;f__Peptostreptococcales-Tissierellales;g__W5053;s__Peptostreptococcaceae_bacterium	0.068	0.025
d__Bacteria;p__Bacteroidota;c__Bacteroidia;o__Bacteroidales;f__Prevotellaceae;g__Prevotella;s__Prevotella_marshii	0.02983	0.02464
d__Bacteria;p__Actinobacteriota;c__Actinobacteria;o__Actinomycetales;f__Actinomycetaceae;g__Actinomyces;s__Actinomyces_genomosp.	0.0065	0.02429
d__Bacteria;p__Actinobacteriota;c__Coriobacteriia;o__Coriobacteriales;f__Atopobiaceae;g__Atopobium;s__Lancefieldella_parvula	0.03417	0.02411
d__Bacteria;p__Bacteroidota;c__Bacteroidia;o__Bacteroidales;f__Prevotellaceae;g__Prevotella;s__Prevotella_buccae	0.06567	0.02357
d__Bacteria;p__Firmicutes;c__Clostridia;o__Peptostreptococcales-Tissierellales;f__Thermotaleaceae;g__Geosporobacter;s__Geosporobacter_ferrireducens	0.01583	0.02339
d__Bacteria;p__Proteobacteria;c__Gammaproteobacteria;o__Pasteurellales;f__Pasteurellaceae;g__Haemophilus;s__Haemophilus_influenzae	0.01083	0.02339

d__Bacteria;p__Firmicutes;c__Clostridia;o__Clostridiales;f__Clostridiaceae;g__Clostridium_sensu_stricto_3;__	0.00883	0.02321
d__Bacteria;p__Proteobacteria;c__Gammaproteobacteria;o__Pasteurellales;f__Pasteurellaceae;g__Haemophilus;s__Haemophilus_pittmaniae	0.00767	0.02321
d__Bacteria;p__Bacteroidota;c__Bacteroidia;o__Flavobacteriales;f__Weeksellaceae;g__Chryseobacterium;s__uncultured_bacterium	0.00783	0.0225
d__Bacteria;p__Firmicutes;c__Bacilli;o__Lactobacillales;f__Streptococcaceae;g__Streptococcus;s__Streptococcus_anginosus	0.096	0.02232
d__Bacteria;p__Firmicutes;c__Clostridia;o__Lachnospirales;f__Lachnospiraceae;g__Lachnoclostridium;s__[Clostridium]_methoxybenzovorans	0.00233	0.02232
d__Bacteria;p__Proteobacteria;c__Gammaproteobacteria;o__Burkholderiales;f__Comamonadaceae;g__Brachymonas;s__unidentified	0.01367	0.02196
d__Bacteria;p__Bacteroidota;c__Bacteroidia;o__Bacteroidales;f__Prevotellaceae;g__Prevotella;s__Prevotella_multiformis	0.01333	0.02125
d__Bacteria;p__Actinobacteriota;c__Actinobacteria;o__Actinomycetales;f__Actinomycetaceae;g__Actinomyces;s__Schaalia_meyeri	0.02033	0.02071
d__Bacteria;p__Proteobacteria;c__Gammaproteobacteria;o__Xanthomonadales;f__Xanthomonadaceae;g__Xanthomonas;__	0.04233	0.02054
d__Bacteria;p__Firmicutes;c__Clostridia;o__Lachnospirales;f__Lachnospiraceae;g__Catonella;s__Catonella_morbi	0.04667	0.02
d__Bacteria;p__Proteobacteria;c__Gammaproteobacteria;o__Cardiobacteriales;f__Cardiobacteriaceae;g__Cardiobacterium;__	0.00533	0.01893
d__Bacteria;p__Firmicutes;c__Clostridia;o__Peptostreptococcales-Tissierellales;f__Peptostreptococcales-Tissierellales;g__Parvimonas;s__Peptostreptococcaceae_bacterium	0.02317	0.01875
d__Bacteria;p__Firmicutes;c__Negativicutes;o__Veillonellales-Selenomonadales;f__Selenomonadaceae;g__Selenomonas;s__Selenomonas_sp.	0.0255	0.01857
d__Bacteria;p__Patescibacteria;c__Saccharimonadia;o__Saccharimonadales;f__Saccharimonadaceae;__;__	0.01033	0.01839
d__Bacteria;p__Firmicutes;c__Clostridia;o__Lachnospirales;f__Lachnospiraceae;g__Catonella;s__uncultured_bacterium	0.01833	0.01821
d__Bacteria;p__Proteobacteria;c__Alphaproteobacteria;o__Caulobacterales;f__Caulobacteraceae;g__Brevundimonas;__	0.0075	0.01821
d__Bacteria;p__Bacteroidota;c__Bacteroidia;o__Bacteroidales;f__Prevotellaceae;g__Prevotella;s__Prevotella_histicola	0.0015	0.01821
d__Bacteria;p__Firmicutes;c__Negativicutes;o__Veillonellales-Selenomonadales;f__Veillonellaceae;g__Megasphaera;s__Megasphaera_micronuciformis	0.0115	0.01786
d__Bacteria;p__Actinobacteriota;c__Actinobacteria;o__Bifidobacteriales;f__Bifidobacteriaceae;g__Scardovia;s__Scardovia_wiggsiae	0.042	0.01768
d__Bacteria;p__Firmicutes;c__Negativicutes;o__Veillonellales-Selenomonadales;f__Selenomonadaceae;g__uncultured;s__Mitsuokella_sp.	0.05467	0.01732
d__Bacteria;p__Firmicutes;c__Clostridia;o__Peptostreptococcales-Tissierellales;f__Peptostreptococcales-Tissierellales;g__Alkaliphilus;__	0.01433	0.01732
d__Bacteria;p__Actinobacteriota;c__Actinobacteria;o__Actinomycetales;f__Actinomycetaceae;g__Actinomyces;s__Actinomyces_dentalis	0.01567	0.01714
d__Bacteria;p__Bacteroidota;c__Bacteroidia;o__Bacteroidales;f__Prevotellaceae;g__Prevotella;s__Prevotella_paludivivens	0.006	0.01696
d__Bacteria;p__Spirochaetota;c__Spirochaetia;o__Spirochaetales;f__Spirochaetaceae;g__Treponema;s__Treponema_parvum	0.01283	0.01643
d__Bacteria;p__Firmicutes;c__Clostridia;o__Peptostreptococcales-Tissierellales;f__Anaerovoracaceae;g__[Eubacterium]_nodatum_group;s__[Eubacterium]_nodatum	0.0345	0.01589
d__Bacteria;p__Proteobacteria;c__Gammaproteobacteria;o__Pseudomonadales;f__Pseudomonadaceae;g__Pseudomonas;__	0.03267	0.01571
d__Bacteria;p__Bacteroidota;c__Bacteroidia;o__Bacteroidales;f__Prevotellaceae;g__Alloprevotella;s__uncultured_Prevotella	0.03733	0.01554
d__Bacteria;p__Bacteroidota;c__Bacteroidia;o__Bacteroidales;f__Prevotellaceae;g__Alloprevotella;s__uncultured_Prevotellaceae	0.31567	0.01536
d__Bacteria;p__Firmicutes;c__Clostridia;o__Lachnospirales;f__Lachnospiraceae;g__Stomatobaculum;s__uncultured_bacterium	0.04083	0.01536
d__Bacteria;p__Proteobacteria;c__Alphaproteobacteria;o__Rhodobacterales;f__Rhodobacteraceae;__;__	0.00517	0.01536
d__Bacteria;p__Firmicutes;c__Clostridia;o__Oscillospirales;f__Ruminococcaceae;g__Caproiciproducens;__	0	0.01464
d__Bacteria;p__Firmicutes;c__Clostridia;o__Peptostreptococcales-Tissierellales;f__Peptostreptococcaceae;g__[Eubacterium]_yurii_group;s__uncultured_bacterium	0.01117	0.01446
d__Bacteria;p__Actinobacteriota;c__Actinobacteria;o__Propionibacteriales;f__Propionibacteriaceae;g__Pseudopropionibacterium;s__Pseudopropionibacterium_propionicum	0.007	0.01429
d__Bacteria;p__Proteobacteria;c__Gammaproteobacteria;o__Enterobacterales;f__Enterobacteriaceae;__;__	0.00417	0.01411
d__Bacteria;p__Bacteroidota;c__Bacteroidia;o__Bacteroidales;f__Bacteroidaceae;g__Bacteroides;s__uncultured_Bacteroides	0.0005	0.01411
d__Bacteria;p__Bacteroidota;c__Bacteroidia;o__Bacteroidales;f__Dysgonomonadaceae;g__Dysgonomonas;__	0.00883	0.01393

d__Bacteria;p__Bacteroidota;c__Bacteroidia;o__Bacteroidales;f__Prevotellaceae;g__Alloprevotella;s__uncultured_Bacteroidetes	0.017	0.01375
d__Bacteria;p__Proteobacteria;c__Alphaproteobacteria;o__Rhodospirillales;f__uncultured;g__uncultured;__	0.002	0.01375
d__Bacteria;p__Firmicutes;c__Negativicutes;o__Veillonellales-Selenomonadales;f__Veillonellaceae;g__Dialister;__	0.01317	0.01357
d__Bacteria;p__Firmicutes;c__Bacilli;o__Lactobacillales;f__Lactobacillaceae;g__Lactobacillus;s__Lactobacillus_harbinensis	0.00333	0.01339
d__Bacteria;p__Firmicutes;c__Bacilli;o__Acholeplasmatales;f__Acholeplasmataceae;g__Acholeplasma;s__Acholeplasmatales_bacterium	0.00233	0.01339
d__Bacteria;p__Firmicutes;c__Clostridia;o__Lachnospirales;f__Lachnospiraceae;g__uncultured;__	0.01283	0.01304
d__Bacteria;p__Bacteroidota;c__Bacteroidia;o__Bacteroidales;f__Prevotellaceae;g__Prevotella;s__Prevotella_nanceiensis	0.011	0.01286
d__Bacteria;p__Firmicutes;c__Clostridia;o__Oscillospirales;f__Ethanoligenenaceae;g__Incertae_Sedis;s__uncultured_Clostridia	0.00083	0.01286
d__Bacteria;p__Actinobacteriota;c__Actinobacteria;o__Propionibacteriales;f__Propionibacteriaceae;g__Pseudopropionibacterium;__	0.01667	0.0125
d__Bacteria;p__Desulfobacterota;c__Desulfovibrionia;o__Desulfovibrionales;f__Desulfovibrionaceae;g__Desulfovibrio;s__Desulfovibrio_fairfieldensis	0.00933	0.01232
d__Bacteria;p__Firmicutes;c__Bacilli;o__Paenibacillales;f__Paenibacillaceae;g__Paenibacillus;__	0.00717	0.01232
d__Bacteria;p__Desulfobacterota;c__Desulfovibrionia;o__Desulfovibrionales;f__Desulfomicrobiaceae;g__Desulfomicrobium;s__Desulfomicrobium_orale	0.00833	0.01179
d__Bacteria;p__Bacteroidota;c__Bacteroidia;o__Bacteroidales;f__Rikenellaceae;g__uncultured;s__uncultured_Clostridium	0.014	0.01089
d__Bacteria;p__Desulfobacterota;c__Desulfovibrionia;o__Desulfovibrionales;f__Desulfovibrionaceae;g__Desulfovibrio;__	0.0155	0.01071
d__Bacteria;p__Firmicutes;c__Bacilli;o__Staphylococcales;f__Staphylococcaceae;g__Staphylococcus;__	0.01683	0.01054
d__Bacteria;p__Fusobacteriota;c__Fusobacteriia;o__Fusobacteriales;f__Fusobacteriaceae;g__Fusobacterium;s__uncultured_bacterium	0	0.01054
d__Bacteria;p__Proteobacteria;c__Alphaproteobacteria;o__Acetobacterales;f__Acetobacteraceae;g__Acetobacter;__	0	0.01036
d__Bacteria;p__Actinobacteriota;c__Actinobacteria;o__Micrococcales;f__Microbacteriaceae;g__Microbacterium;__	0.009	0.01
d__Bacteria;p__Firmicutes;c__Bacilli;o__Lactobacillales;f__Lactobacillaceae;g__Lactobacillus;s__Lactobacillus_gasseri	0.12567	0.00964
d__Bacteria;p__Actinobacteriota;c__Actinobacteria;o__Actinomycetales;f__Actinomycetaceae;g__Actinomyces;s__Actinomyces_israelii	0.01233	0.00946
d__Bacteria;p__Bacteroidota;c__Bacteroidia;o__Cytophagales;f__Cyclobacteriaceae;g__Mongoliitalea;s__bacterium_SL3.16	0.00567	0.00946
d__Bacteria;p__Firmicutes;c__Negativicutes;o__Veillonellales-Selenomonadales;f__Veillonellaceae;g__Dialister;s__Dialister_micraerophilus	0.01033	0.00929
d__Bacteria;p__Campilobacterota;c__Campylobacteria;o__Campylobacterales;f__Helicobacteraceae;g__Wolinella;s__Wolinella_sp.	0.00467	0.00929
d__Bacteria;p__Proteobacteria;c__Gammaproteobacteria;o__Alteromonadales;f__Alteromonadales_Incertae_Sedis;g__Alkalimonas;__	0.011	0.00911
d__Bacteria;p__Campilobacterota;c__Campylobacteria;o__Campylobacterales;f__Rs-M59_termite_group;g__Rs-M59_termite_group;s__uncultured_bacterium	0.001	0.00911
d__Bacteria;p__Firmicutes;c__Bacilli;o__Lactobacillales;__;__;__	0.01333	0.00893
d__Bacteria;p__Firmicutes;c__Clostridia;o__Oscillospirales;f__Oscillospirales;g__Hydrogenoanaerobacterium;s__uncultured_bacterium	0.00567	0.00893
d__Bacteria;p__Proteobacteria;c__Alphaproteobacteria;o__Rhodospirillales;f__Rhodospirillaceae;g__Candidatus_Riegeria;s__uncultured_bacterium	0.005	0.00893
d__Bacteria;p__Bacteroidota;c__Bacteroidia;o__Bacteroidales;f__Marinilabiliaceae;g__Alkaliflexus;s__uncultured_bacterium	0.00183	0.00893
d__Bacteria;p__Firmicutes;c__Clostridia;o__Lachnospirales;f__Lachnospiraceae;g__Johnsonella;s__Firmicutes_oral	0	0.00893
d__Bacteria;p__Actinobacteriota;c__Actinobacteria;o__Bifidobacteriales;f__Bifidobacteriaceae;g__Parascardovia;s__Parascardovia_denticolens	0.00533	0.00875
d__Bacteria;p__Spirochaetota;c__Spirochaetia;o__Spirochaetales;f__Spirochaetaceae;g__Treponema;s__Treponema_pedis	0	0.00875
d__Bacteria;p__Actinobacteriota;c__Coriobacteriia;o__Coriobacteriales;f__Atopobiaceae;g__Atopobium;s__Lancefieldella_rimae	0.04217	0.00857
d__Bacteria;p__Proteobacteria;c__Alphaproteobacteria;o__Rhodospirillales;f__uncultured;g__uncultured;s__uncultured_bacterium	0.0025	0.00839
d__Bacteria;p__Proteobacteria;c__Alphaproteobacteria;o__Acetobacterales;f__Acetobacteraceae;g__Acetobacter;s__uncultured_bacterium	0	0.00839
d__Bacteria;p__Firmicutes;c__Clostridia;o__Oscillospirales;f__Ethanoligenenaceae;g__Incertae_Sedis;s__uncultured_Ruminococcaceae	0	0.00804

d__Bacteria;p__Patescibacteria;c__Saccharimonadia;o__Saccharimonadales;f__Saccharimonadales;g__Saccharimonadales;s__TM7_phylum	0.0275	0.00786
d__Bacteria;p__Proteobacteria;c__Gammaproteobacteria;o__Burkholderiales;f__Burkholderiaceae;g__Burkholderia-Caballeronia-Paraburkholderia;s__Paraburkholderia_caledonica	0.0065	0.00786
d__Bacteria;p__Firmicutes;c__Clostridia;o__Eubacteriales;f__Eubacteriaceae;g__Pseudoramibacter;__	0.027	0.00768
d__Bacteria;p__Firmicutes;c__Bacilli;o__Erysipelotrichales;f__Erysipelotrichaceae;g__Erysipelotrichaceae_UCG-006;__	0.00533	0.00768
d__Bacteria;p__Firmicutes;c__Bacilli;o__Mycoplasmatales;f__Mycoplasmataceae;g__Mycoplasma;s__Mycoplasmopsis_lipophila	0.00233	0.00768
d__Bacteria;p__Firmicutes;c__Clostridia;o__Lachnospirales;f__Lachnospiraceae;g__Oribacterium;s__uncultured_organism	0.009	0.00732
d__Bacteria;p__Firmicutes;c__Negativicutes;o__Veillonellales-Selenomonadales;f__Selenomonadaceae;g__Selenomonas;s__Selenomonas_flueggei	0	0.00732
d__Bacteria;p__Firmicutes;c__Clostridia;o__Lachnospirales;f__Lachnospiraceae;g__Butyrivibrio;s__Firmicutes_oral	0.01817	0.00714
d__Bacteria;p__Spirochaetota;c__Spirochaetia;o__Spirochaetales;f__Spirochaetaceae;g__Sediminispirochaeta;s__Spirochaeta_sp.	0	0.00714
d__Bacteria;p__Campilobacterota;c__Campylobacteria;o__Campylobacterales;f__Campylobacteraceae;g__Campylobacter;s__unidentified	0.06517	0.00696
d__Bacteria;p__Proteobacteria;c__Alphaproteobacteria;o__Sphingomonadales;f__Sphingomonadaceae;g__Sphingobium;__	0.00317	0.00625
d__Bacteria;p__Patescibacteria;c__Microgenomatia;o__Candidatus_Pacebacteria;f__Candidatus_Pacebacteria;g__Candidatus_Pacebacteria;s__uncultured_bacterium	0.01667	0.00607
d__Bacteria;p__Proteobacteria;c__Gammaproteobacteria;o__Oceanospirillales;f__Nitrincolaceae;g__Nitrincola;s__uncultured_bacterium	0.00333	0.00607
d__Bacteria;p__Actinobacteriota;c__Actinobacteria;o__Propionibacteriales;f__Propionibacteriaceae;g__Aestuariimicrobium;s__uncultured_Propionibacteriaceae	0.00117	0.00607
d__Bacteria;p__Bacteroidota;c__Bacteroidia;o__Bacteroidales;f__Prevotellaceae;g__Prevotellaceae;s__Prevotella_sp.	0.0015	0.00589
d__Bacteria;p__Proteobacteria;c__Alphaproteobacteria;__;__;__;__	0.00083	0.00589
d__Bacteria;p__Firmicutes;c__Clostridia;o__Oscillospirales;f__Ruminococcaceae;g__Anaerofilum;s__uncultured_Anaerofilum	0.00183	0.00571
d__Bacteria;p__Firmicutes;c__Bacilli;o__Acholeplasmatales;f__Acholeplasmataceae;g__Acholeplasma;s__Acholeplasma_brassicae	0.0035	0.00554
d__Bacteria;p__Proteobacteria;c__Alphaproteobacteria;o__Rhodospirillales;__;__;__	0.00067	0.00554
d__Bacteria;p__Proteobacteria;c__Gammaproteobacteria;o__Burkholderiales;f__Neisseriaceae;g__Neisseria;s__Eikenella_sp.	0.00183	0.00518
d__Bacteria;p__Proteobacteria;c__Gammaproteobacteria;o__Alteromonadales;f__Shewanellaceae;g__Shewanella;s__Shewanella_putrefaciens	0.001	0.00518
d__Bacteria;p__Firmicutes;c__Clostridia;o__Peptostreptococcales-Tissierellales;f__Anaerovoracaceae;g__[Eubacterium]_nodatum_group;s__[Eubacterium]_infirmum	0.01867	0.005
d__Bacteria;p__Firmicutes;c__Negativicutes;o__Veillonellales-Selenomonadales;f__Veillonellaceae;__;__	0.0075	0.005
d__Bacteria;p__Firmicutes;c__Clostridia;o__Lachnospirales;f__Lachnospiraceae;g__Shuttleworthia;s__Shuttleworthia_satelles	0.05083	0.00482
d__Bacteria;p__Firmicutes;c__Clostridia;o__Lachnospirales;f__Lachnospiraceae;g__Lachnoanaerobaculum;s__Lachnospiraceae_oral	0.00967	0.00482
d__Bacteria;p__Firmicutes;c__Clostridia;o__Peptostreptococcales-Tissierellales;f__Anaerovoracaceae;g__Family_XIII_UCG-001;s__Eubacterium_sp.	0.007	0.00482
d__Bacteria;p__Actinobacteriota;c__Coriobacteriia;o__Coriobacteriales;f__Atopobiaceae;g__Olsenella;s__Olsenella_uli	0.0265	0.00464
d__Bacteria;p__Proteobacteria;c__Gammaproteobacteria;o__Pasteurellales;f__Pasteurellaceae;g__Haemophilus;s__Haemophilus_sp.	0.01683	0.00464
d__Bacteria;p__Desulfobacterota;c__Desulfuromonadia;o__Bradymonadales;f__Bradymonadales;g__Bradymonadales;s__uncultured_rumen	0.0005	0.00464
d__Bacteria;p__Firmicutes;c__Bacilli;o__Erysipelotrichales;f__Erysipelotrichaceae;g__Solobacterium;__	0.00033	0.00464
d__Bacteria;p__Actinobacteriota;c__Coriobacteriia;o__Coriobacteriales;f__Atopobiaceae;g__Olsenella;__	0.02533	0.00446
d__Bacteria;p__Patescibacteria;c__Saccharimonadia;o__Saccharimonadales;f__Saccharimonadales;g__Saccharimonadales;__	0.00533	0.00429
d__Bacteria;p__Actinobacteriota;c__Actinobacteria;o__Propionibacteriales;f__Propionibacteriaceae;g__Cutibacterium;__	0.00133	0.00429
d__Bacteria;p__Bacteroidota;c__Bacteroidia;o__Bacteroidales;f__Porphyromonadaceae;g__Porphyromonas;s__Porphyromonas_uenonis	0.0095	0.00411
d__Bacteria;p__Proteobacteria;c__Alphaproteobacteria;o__Rhizobiales;f__Rhizobiaceae;g__Allorhizobium-Neorhizobium-Pararhizobium-Rhizobium;__	0.00817	0.00411
d__Bacteria;p__Firmicutes;c__Clostridia;o__Lachnospirales;f__Lachnospiraceae;g__Anaerostignum;s__Anaerotignum_propionicum	0.00317	0.00411

d__Bacteria;p__Bacteroidota;c__Bacteroidia;o__Cytophagales;f__Spirosomaceae;g__Dyadobacter;__	0	0.00411
d__Bacteria;p__Firmicutes;c__Negativicutes;o__Veillonellales-Selenomonadales;f__Veillonellaceae;g__Veillonella;s__Veillonella_sp.	0.0035	0.00393
d__Bacteria;p__Proteobacteria;c__Gammaproteobacteria;o__Pseudomonadales;f__Moraxellaceae;g__Acinetobacter;s__Acinetobacter_soli	0.00317	0.00393
d__Bacteria;p__Bacteroidota;c__Bacteroidia;o__Bacteroidales;f__Paludibacteraceae;g__uncultured;s__uncultured_bacterium	0.00283	0.00393
d__Bacteria;p__Actinobacteriota;c__Actinobacteria;o__Propionibacteriales;f__Propionibacteriaceae;g__Tessaracoccus;__	0.00117	0.00393
d__Bacteria;p__Bacteroidota;c__Bacteroidia;o__Bacteroidales;f__Bacteroidaceae;g__Bacteroides;s__Bacteroides_reticulotermitis	0.0005	0.00393
d__Bacteria;p__Bacteroidota;c__Bacteroidia;o__Bacteroidales;f__Bacteroidaceae;g__Bacteroides;s__Bacteroides_sp.	0.00033	0.00375
d__Bacteria;p__Firmicutes;c__Clostridia;o__Lachnospirales;f__Lachnospiraceae;g__Lachnoanaerobaculum;s__Eubacterium_sp.	0.00417	0.00357
d__Bacteria;p__Bacteroidota;c__Bacteroidia;o__Bacteroidales;f__Bacteroidaceae;g__Bacteroides;s__Bacteroides_graminisolvens	0.00183	0.00357
d__Bacteria;p__Firmicutes;c__Clostridia;o__Peptostreptococcales-Tissierellales;f__Anaerovoracaceae;g__Family_XIII_UCG-001;s__Peptostreptococcaceae_bacterium	0.00067	0.00357
d__Bacteria;p__Desulfobacterota;c__Desulfovibrionia;o__Desulfovibrionales;f__Desulfoplanaceae;g__Desulfoplanes;s__uncultured_bacterium	0	0.00357
d__Bacteria;p__Bacteroidota;c__Bacteroidia;__;__;__;__	0.00083	0.00339
d__Bacteria;p__Firmicutes;c__Negativicutes;o__Veillonellales-Selenomonadales;f__Veillonellaceae;g__Anaeroglobus;s__uncultured_organism	0.00433	0.00321
d__Bacteria;p__Actinobacteriota;c__Actinobacteria;o__Micrococcales;f__Cellulomonadaceae;g__Actinotalea;s__uncultured_bacterium	0	0.00321
d__Bacteria;p__Proteobacteria;c__Gammaproteobacteria;o__Burkholderiales;f__Neisseriaceae;g__Kingella;s__uncultured_Kingella	0	0.00304
d__Bacteria;p__Firmicutes;c__Bacilli;o__Erysipelotrichales;f__Erysipelotrichaceae;g__Bulleidia;s__Bulleidia_extructa	0.00633	0.00286
d__Bacteria;p__Fusobacteriota;c__Fusobacteriia;o__Fusobacteriales;f__Fusobacteriaceae;g__Fusobacterium;s__Fusobacterium_sp.	0.0005	0.00286
d__Bacteria;p__Bacteroidota;c__Bacteroidia;o__Cytophagales;f__Spirosomaceae;g__Flectobacillus;s__Flectobacillus_sp.	0.00033	0.00286
d__Bacteria;p__Firmicutes;c__Clostridia;o__Peptostreptococcales-Tissierellales;f__Anaerovoracaceae;g__[Eubacterium]_nodatum_group;s__[Eubacterium]_minutum	0.00967	0.00268
d__Bacteria;p__Bacteroidota;c__Bacteroidia;o__Bacteroidales;f__Prevotellaceae;g__Prevotellaceae_UCG-004;s__uncultured_bacterium	0.00333	0.00268
d__Bacteria;p__Bacteroidota;c__Bacteroidia;o__Cytophagales;f__Cyclobacteriaceae;g__Mongoliitalea;__	0	0.00268
d__Bacteria;p__Firmicutes;c__Clostridia;o__Peptostreptococcales-Tissierellales;f__Anaerovoracaceae;g__Mogibacterium;s__Mogibacterium_timidum	0.00517	0.0025
d__Bacteria;p__Proteobacteria;c__Gammaproteobacteria;o__Burkholderiales;f__Comamonadaceae;g__Comamonas;s__Comamonas_terrigena	0	0.0025
d__Bacteria;p__Patescibacteria;c__Saccharimonadia;o__Saccharimonadales;f__Saccharimonadaceae;g__TM7a;s__uncultured_bacterium	0.15483	0.00232
d__Bacteria;p__Firmicutes;c__Clostridia;o__Lachnospirales;f__Lachnospiraceae;g__Lachnotalea;s__uncultured_bacterium	0.00333	0.00232
d__Bacteria;p__Bacteroidota;c__Bacteroidia;o__Flavobacteriales;f__Crocinitomicaceae;__;__	0.00067	0.00232
d__Bacteria;p__Bacteroidota;c__Bacteroidia;o__Sphingobacteriales;f__Sphingobacteriaceae;g__Sphingobacterium;s__Sphingobacterium_faecium	0.00067	0.00232
d__Bacteria;p__Patescibacteria;c__Dojkabacteria;o__Dojkabacteria;f__Dojkabacteria;g__Dojkabacteria;__	0.00017	0.00232
d__Bacteria;p__Firmicutes;c__Bacilli;o__Bacillales;f__Sporolactobacillaceae;g__Sporolactobacillus;s__uncultured_Bacillus	0	0.00232
d__Bacteria;p__Firmicutes;c__Bacilli;o__RF39;f__RF39;g__RF39;s__uncultured_bacterium	0.00467	0.00214
d__Bacteria;p__Actinobacteriota;c__Coriobacteriia;o__Coriobacteriales;f__Atopobiaceae;g__Atopobium;__	0.0145	0.00196
d__Bacteria;p__Actinobacteriota;c__Actinobacteria;o__Propionibacteriales;f__Propionibacteriaceae;g__Propionibacterium;s__Propionibacterium_sp.	0.003	0.00196
d__Bacteria;p__Bacteroidota;c__Bacteroidia;o__Flavobacteriales;f__Cryomorphaceae;g__Owenweeksia;s__uncultured_bacterium	0.0015	0.00196
d__Bacteria;p__Firmicutes;c__Clostridia;o__Oscillospirales;f__Oscillospiraceae;g__Oscillibacter;s__uncultured_Clostridiales	0	0.00196
d__Bacteria;p__Firmicutes;c__Clostridia;o__Oscillospirales;f__Ruminococcaceae;g__Caproiciproducens;s__uncultured_Clostridia	0	0.00196
d__Bacteria;p__Firmicutes;c__Clostridia;o__Oscillospirales;f__Oscillospiraceae;g__Oscillibacter;s__uncultured_Oscillibacter	0.00167	0.00179

d__Bacteria;p__Proteobacteria;c__Gammaproteobacteria;o__Pasteurellales;f__Pasteurellaceae;g__Haemophilus;s__Haemophilus_haemolyticus	0.0015	0.00179
d__Bacteria;p__Proteobacteria;c__Alphaproteobacteria;o__Sphingomonadales;f__Sphingomonadaceae;g__Novosphingobium;s__uncultured_organism	0.0005	0.00179
d__Bacteria;p__Bacteroidota;c__Bacteroidia;o__Bacteroidales;f__Prevotellaceae;g__Prevotella;s__Prevotella_enoeca	0.01117	0.00161
d__Bacteria;p__Firmicutes;c__Clostridia;o__Peptostreptococcales-Tissierellales;f__Peptostreptococcales-Tissierellales;g__uncultured;s__Tissierella_sp.	0.0075	0.00161
d__Bacteria;p__Bacteroidota;c__Bacteroidia;o__Cytophagales;f__Spirosomaceae;g__Persicitalea;s__uncultured_bacterium	0.0005	0.00161
d__Bacteria;p__Actinobacteriota;c__Actinobacteria;o__Bifidobacteriales;f__Bifidobacteriaceae;g__Bifidobacterium;s__Bifidobacterium_subtile	0	0.00161
d__Bacteria;p__Actinobacteriota;c__Actinobacteria;o__Frankiales;f__Geodermatophilaceae;g__Modestobacter;__	0	0.00161
d__Bacteria;p__Bacteroidota;c__Bacteroidia;o__Flavobacteriales;f__Flavobacteriaceae;g__Flavobacterium;s__unidentified	0	0.00161
d__Bacteria;p__Bacteroidota;c__Bacteroidia;o__Flavobacteriales;f__Weeksellaceae;g__uncultured;s__Daejeonia_ginsenosidivorans	0	0.00161
d__Bacteria;p__Proteobacteria;c__Gammaproteobacteria;o__Burkholderiales;f__Neisseriaceae;g__Kingella;s__Neisseria_sp.	0.00233	0.00143
d__Bacteria;p__Proteobacteria;c__Gammaproteobacteria;o__Cardiobacteriales;f__Cardiobacteriaceae;g__Suttonella;s__uncultured_bacterium	0	0.00143
d__Bacteria;p__Proteobacteria;c__Gammaproteobacteria;o__Enterobacterales;f__Erwiniaceae;__;__	0	0.00143
d__Bacteria;p__Proteobacteria;c__Gammaproteobacteria;o__Oceanospirillales;f__Nitrincolaceae;g__Nitrincola;__	0	0.00143
d__Bacteria;p__Bacteroidota;c__Bacteroidia;o__Bacteroidales;f__Prevotellaceae;g__uncultured;s__uncultured_Prevotella	0.01317	0.00125
d__Bacteria;p__Actinobacteriota;c__Actinobacteria;o__Micrococcales;f__Cellulomonadaceae;g__Cellulomonas;__	0.0025	0.00125
d__Bacteria;p__Bacteroidota;c__Bacteroidia;o__Bacteroidales;f__Bacteroidaceae;g__Bacteroides;s__Prevotella_heparinolytica	0.00117	0.00125
d__Bacteria;p__Actinobacteriota;c__Actinobacteria;o__Actinomycetales;f__Actinomycetaceae;g__Actinomyces;s__Actinomyces_timonensis	0	0.00125
d__Bacteria;p__Actinobacteriota;c__Coriobacteriia;o__Coriobacteriales;f__Eggerthellaceae;__;__	0	0.00125
d__Bacteria;p__Firmicutes;c__Clostridia;o__Oscillospirales;f__Oscillospiraceae;__;__	0	0.00125
d__Bacteria;p__Proteobacteria;c__Alphaproteobacteria;o__Sphingomonadales;f__Sphingomonadaceae;g__Rhizorhapis;s__uncultured_bacterium	0	0.00125
d__Bacteria;p__Campilobacterota;c__Campylobacteria;o__Campylobacterales;f__Campylobacteraceae;g__Campylobacter;s__Campylobacter_curvus	0.01433	0.00107
d__Bacteria;p__Elusimicrobiota;c__Elusimicrobia;o__Lineage_IV;f__Lineage_IV;g__Lineage_IV;s__uncultured_bacterium	0.00583	0.00107
d__Bacteria;p__Bacteroidota;c__Bacteroidia;o__Bacteroidales;f__Prevotellaceae;g__Prevotella;s__uncultured_Prevotella	0.0025	0.00107
d__Bacteria;p__Firmicutes;c__Clostridia;o__Oscillospirales;f__Ruminococcaceae;g__Ruminococcus;__	0.0015	0.00107
d__Bacteria;p__Proteobacteria;c__Alphaproteobacteria;o__Sphingomonadales;f__Sphingomonadaceae;g__Sphingomonas;__	0.0015	0.00107
d__Bacteria;p__Bacteroidota;c__Bacteroidia;o__Chitinophagales;f__Chitinophagaceae;g__Taibaiella;s__bacterium_enrichment	0.0005	0.00107
d__Bacteria;p__Firmicutes;c__Negativicutes;o__Veillonellales-Selenomonadales;f__Selenomonadaceae;g__Selenomonas;s__Selenomonas_genomosp.	0.00033	0.00107
d__Bacteria;p__Actinobacteriota;c__Actinobacteria;o__Corynebacteriales;f__Corynebacteriaceae;g__Lawsonella;s__uncultured_bacterium	0	0.00107
d__Bacteria;p__Bacteroidota;c__Bacteroidia;o__Bacteroidales;f__Prevotellaceae;g__Prevotella;s__Prevotella_copri	0	0.00107
d__Bacteria;p__Firmicutes;c__Clostridia;o__Oscillospirales;f__Ruminococcaceae;g__Candidatus_Soleaferrea;s__uncultured_rumen	0	0.00107
d__Bacteria;p__Firmicutes;c__Clostridia;o__Clostridia_UCG-014;f__Clostridia_UCG-014;g__Clostridia_UCG-014;s__uncultured_bacterium	0.00817	0.00089
d__Bacteria;p__Firmicutes;c__Bacilli;o__Erysipelotrichales;f__Erysipelotrichaceae;g__Erysipelotrichaceae_UCG-006;s__uncultured_Erysipelotrichaceae	0.00483	0.00089
d__Bacteria;p__Firmicutes;c__Clostridia;o__Lachnospirales;f__Lachnospiraceae;g__Anaerocolumna;s__Clostridium_sp.	0.002	0.00089
d__Bacteria;p__Proteobacteria;c__Alphaproteobacteria;o__Azospirillales;f__Azospirillaceae;g__Nitrospirillum;s__Rhodospirillaceae_bacterium	0.00133	0.00089
d__Eukaryota;p__Parabasalia;c__Trichomonadea;o__Trichomonadea;f__Trichomonadea;g__Trichomonas;s__Trichomonas_tenax	0.00133	0.00089
d__Bacteria;p__Proteobacteria;c__Gammaproteobacteria;o__Alteromonadales;f__Alteromonadales_Incertae_Sedis;g__Alkalimonas;s__Alkalimonas_collagenimarina	0.00067	0.00089

d__Bacteria;p__Proteobacteria;c__Gammaproteobacteria;o__Pseudomonadales;f__Moraxellaceae;g__Acinetobacter;__	0.00033	0.00089
d__Bacteria;p__Actinobacteriota;c__Actinobacteria;o__Micrococcales;f__Microbacteriaceae;__;__	0	0.00089
d__Bacteria;p__Bacteroidota;c__Bacteroidia;o__Sphingobacteriales;f__Sphingobacteriaceae;g__Sphingobacterium;__	0	0.00089
d__Bacteria;p__Firmicutes;c__Clostridia;o__Peptostreptococcales-Tissierellales;f__Peptostreptococcaceae;__;__	0	0.00089
d__Bacteria;p__Firmicutes;c__Clostridia;o__Peptostreptococcales-Tissierellales;f__Peptostreptococcales-Tissierellales;g__Anaerococcus;s__Anaerococcus_prevotii	0	0.00089
d__Bacteria;p__Proteobacteria;c__Gammaproteobacteria;o__Cellvibrionales;f__Cellvibrionaceae;g__Cellvibrio;s__Cellvibrio_sp.	0	0.00089
d__Bacteria;p__Proteobacteria;c__Gammaproteobacteria;o__Enterobacterales;f__Erwiniaceae;g__Pantoea;__	0	0.00089
d__Bacteria;p__Proteobacteria;c__Gammaproteobacteria;o__Pasteurellales;f__Pasteurellaceae;g__Actinobacillus;s__Haemophilus_parahaemolyticus	0.015	0.00071
d__Bacteria;p__Bacteroidota;c__Bacteroidia;o__Bacteroidales;f__Prevotellaceae;g__Prevotella;s__Prevotella_aurantiaca	0.00383	0.00071
d__Bacteria;p__Bacteroidota;c__Bacteroidia;o__Cytophagales;f__Cyclobacteriaceae;g__Mongoliitalea;s__Mongoliitalea_lutea	0.00067	0.00071
d__Bacteria;p__Actinobacteriota;c__Actinobacteria;o__Propionibacteriales;f__Propionibacteriaceae;g__Acidipropionibacterium;s__Acidipropionibacterium_thoenii	0	0.00071
d__Bacteria;p__Fusobacteriota;c__Fusobacteriia;o__Fusobacteriales;f__Leptotrichiaceae;g__Leptotrichia;s__Leptotrichia_hongkongensis	0	0.00071
d__Bacteria;p__Proteobacteria;c__Alphaproteobacteria;o__Rhizobiales;f__Xanthobacteraceae;g__Pseudolabrys;__	0	0.00071
d__Bacteria;p__Proteobacteria;c__Alphaproteobacteria;o__Sphingomonadales;f__Sphingomonadaceae;g__Sphingobium;s__Sphingobium_yanoikuyae	0	0.00071
d__Bacteria;p__Firmicutes;c__Bacilli;o__Bacillales;f__Bacillaceae;g__Bacillus;__	0.003	0.00054
d__Bacteria;p__Verrucomicrobiota;c__Verrucomicrobiae;o__Opitutales;f__Puniceicoccaceae;g__Cerasicoccus;s__uncultured_bacterium	0.00217	0.00054
d__Bacteria;p__Proteobacteria;c__Gammaproteobacteria;o__Aeromonadales;f__Aeromonadaceae;g__Aeromonas;__	0.00133	0.00054
d__Bacteria;p__Actinobacteriota;c__Actinobacteria;o__Micrococcales;f__Microbacteriaceae;g__Leucobacter;s__uncultured_bacterium	0.00083	0.00054
d__Bacteria;p__Actinobacteriota;c__Actinobacteria;o__Micrococcales;__;__;__	0.0005	0.00054
d__Bacteria;p__Actinobacteriota;c__Actinobacteria;o__Bifidobacteriales;f__Bifidobacteriaceae;g__Gardnerella;s__Gardnerella_vaginalis	0	0.00054
d__Bacteria;p__Bacteroidota;c__Bacteroidia;o__Bacteroidales;f__Porphyromonadaceae;g__Porphyromonas;s__Porphyromonas_gulae	0	0.00054
d__Bacteria;p__Firmicutes;c__Bacilli;o__Erysipelotrichales;f__Erysipelatoclostridiaceae;g__UCG-004;s__uncultured_bacterium	0	0.00054
d__Bacteria;p__Proteobacteria;c__Gammaproteobacteria;o__Burkholderiales;f__Comamonadaceae;g__Aquabacterium;__	0	0.00054
d__Bacteria;p__Proteobacteria;c__Gammaproteobacteria;o__Burkholderiales;f__Comamonadaceae;g__Hydrogenophaga;__	0	0.00054
d__Bacteria;p__Proteobacteria;c__Gammaproteobacteria;o__Pseudomonadales;f__Moraxellaceae;g__Acinetobacter;s__Acinetobacter_guillouiae	0	0.00054
d__Bacteria;p__Proteobacteria;c__Gammaproteobacteria;o__Pseudomonadales;f__Pseudomonadaceae;g__Pseudomonas;s__Azomonas_agilis	0	0.00054
d__Bacteria;p__Proteobacteria;c__Gammaproteobacteria;o__Cellvibrionales;f__Cellvibrionaceae;g__Cellvibrio;__	0.001	0.00036
d__Bacteria;p__Actinobacteriota;c__Actinobacteria;o__Bifidobacteriales;f__Bifidobacteriaceae;g__Bifidobacterium;__	0.00067	0.00036
d__Bacteria;p__Bacteroidota;c__Bacteroidia;o__Sphingobacteriales;f__Sphingobacteriaceae;g__Sphingobacterium;s__Sphingobacterium_psychroaquaticum	0	0.00036
d__Bacteria;p__Firmicutes;c__Bacilli;o__Erysipelotrichales;f__Erysipelotrichaceae;g__Erysipelothrix;s__uncultured_Firmicutes	0	0.00036
d__Bacteria;p__Firmicutes;c__Bacilli;o__RF39;f__RF39;g__RF39;__	0	0.00036
d__Bacteria;p__Firmicutes;c__Clostridia;o__Lachnospirales;f__Lachnospiraceae;g__Howardella;__	0	0.00036
d__Bacteria;p__Firmicutes;c__Clostridia;o__Peptostreptococcales-Tissierellales;f__Anaerovoracaceae;g__Amnipila;s__Peptostreptococcaceae_bacterium	0	0.00036
d__Bacteria;p__Firmicutes;c__Negativicutes;o__Veillonellales-Selenomonadales;f__Veillonellaceae;g__Megasphaera;s__Megasphaera_elsdenii	0	0.00036
d__Bacteria;p__Proteobacteria;c__Alphaproteobacteria;o__Caulobacterales;f__Caulobacteraceae;g__Brevundimonas;s__Brevundimonas_terrae	0	0.00036
d__Bacteria;p__Proteobacteria;c__Alphaproteobacteria;o__Rhizobiales;f__Devosiaceae;g__Pelagibacterium;__	0	0.00036

d__Bacteria;p__Proteobacteria;c__Gammaproteobacteria;o__Burkholderiales;f__Burkholderiaceae;g__Burkholderia-Caballeronia-Paraburkholderia;s__Paraburkholderia_bryophila	0	0.00036
d__Bacteria;p__Proteobacteria;c__Gammaproteobacteria;o__Burkholderiales;f__Burkholderiaceae;g__Cupriavidus;s__Cupriavidus_basilensis	0	0.00036
d__Bacteria;p__Proteobacteria;c__Gammaproteobacteria;o__Pasteurellales;f__Pasteurellaceae;__;__	0	0.00036
d__Bacteria;p__Fusobacteriota;c__Fusobacteriia;o__Fusobacteriales;f__Leptotrichiaceae;g__Leptotrichia;s__Leptotrichia_shahii	0.01167	0.00018
d__Bacteria;p__Bacteroidota;c__Bacteroidia;o__Bacteroidales;f__Prevotellaceae;g__Prevotella;s__Prevotella_scopos	0.0085	0.00018
d__Bacteria;p__Firmicutes;c__Clostridia;o__Lachnospirales;f__Lachnospiraceae;g__Howardella;s__Eubacterium_sp.	0.003	0.00018
d__Bacteria;p__Proteobacteria;c__Alphaproteobacteria;o__Sphingomonadales;f__Sphingomonadaceae;g__Novosphingobium;s__Novosphingobium_capsulatum	0.00217	0.00018
d__Bacteria;p__Campilobacterota;c__Campylobacteria;o__Campylobacterales;f__Campylobacteraceae;g__Campylobacter;s__uncultured_Treponema	0.0005	0.00018
d__Bacteria;p__Proteobacteria;c__Alphaproteobacteria;o__Acetobacterales;f__Acetobacteraceae;g__Roseomonas;s__Roseomonas_cervicalis	0.0005	0.00018
d__Bacteria;p__Firmicutes;c__Clostridia;o__Oscillospirales;f__Oscillospiraceae;g__Colidextribacter;__	0	0.00018
d__Bacteria;p__Firmicutes;c__Clostridia;o__Oscillospirales;f__Ruminococcaceae;g__Caproiciproducens;s__uncultured_organism	0	0.00018
d__Bacteria;p__Firmicutes;c__Clostridia;o__Oscillospirales;f__UCG-011;g__UCG-011;s__uncultured_bacterium	0	0.00018
d__Bacteria;p__Firmicutes;c__Clostridia;o__Peptostreptococcales-Tissierellales;f__Peptostreptococcales-Tissierellales;g__Peptoniphilus;s__uncultured_organism	0	0.00018
d__Bacteria;p__Firmicutes;c__Negativicutes;o__Acidaminococcales;f__Acidaminococcaceae;g__Acidaminococcus;s__uncultured_bacterium	0	0.00018
d__Bacteria;p__Proteobacteria;c__Alphaproteobacteria;o__Rhizobiales;f__Stappiaceae;g__Pannonibacter;s__Pannonibacter_phragmitetus	0	0.00018
d__Bacteria;p__Proteobacteria;c__Gammaproteobacteria;o__Pseudomonadales;f__Moraxellaceae;g__Moraxella;__	0.0625	0
d__Bacteria;p__Actinobacteriota;c__Actinobacteria;o__Actinomycetales;f__Actinomycetaceae;g__Actinomyces;s__Actinomyces_oris	0.0185	0
d__Bacteria;p__Proteobacteria;c__Gammaproteobacteria;o__Pasteurellales;f__Pasteurellaceae;g__Haemophilus;s__Haemophilus_sputorum	0.0185	0
d__Bacteria;p__Proteobacteria;c__Gammaproteobacteria;o__Pasteurellales;f__Pasteurellaceae;g__Aggregatibacter;s__Aggregatibacter_sp.	0.01817	0
d__Bacteria;p__Firmicutes;c__Clostridia;o__Lachnospirales;f__Lachnospiraceae;g__uncultured;s__Eubacterium_sp.	0.01683	0
d__Bacteria;p__Actinobacteriota;c__Actinobacteria;o__Actinomycetales;f__Actinomycetaceae;g__Actinomyces;s__Actinomyces_oricola	0.016	0
d__Bacteria;p__Fusobacteriota;c__Fusobacteriia;o__Fusobacteriales;f__Leptotrichiaceae;g__Streptobacillus;__	0.015	0
d__Bacteria;p__Firmicutes;c__Bacilli;o__Lactobacillales;f__Streptococcaceae;g__Lactococcus;s__Lactococcus_raffinolactis	0.01317	0
d__Bacteria;p__Bacteroidota;c__Bacteroidia;o__Bacteroidales;f__Bacteroidaceae;g__Bacteroides;s__Bacteroides_fragilis	0.013	0
d__Bacteria;p__Firmicutes;c__Clostridia;o__Lachnospirales;f__Lachnospiraceae;g__Lachnoanaerobaculum;s__uncultured_Lachnospiraceae	0.013	0
d__Bacteria;p__Firmicutes;c__Bacilli;o__Bacillales;f__Bacillaceae;__;__	0.01067	0
d__Bacteria;p__Campilobacterota;c__Campylobacteria;o__Campylobacterales;f__Campylobacteraceae;g__Campylobacter;s__uncultured_Campylobacter	0.00783	0
d__Bacteria;p__Firmicutes;c__Clostridia;o__Lachnospirales;f__Lachnospiraceae;g__Oribacterium;s__uncultured_Lachnospiraceae	0.00733	0
d__Bacteria;p__Actinobacteriota;c__Actinobacteria;o__Bifidobacteriales;f__Bifidobacteriaceae;g__Bifidobacterium;s__Bifidobacterium_dentium	0.00683	0
d__Bacteria;p__Firmicutes;c__Bacilli;o__Mycoplasmatales;f__Mycoplasmataceae;g__Ureaplasma;s__Ureaplasma_sp.	0.00683	0
d__Bacteria;p__Firmicutes;c__Negativicutes;o__Veillonellales-Selenomonadales;f__Veillonellaceae;g__Dialister;s__uncultured_bacterium	0.00567	0
d__Bacteria;p__Firmicutes;c__Negativicutes;o__Veillonellales-Selenomonadales;f__Selenomonadaceae;g__uncultured;s__uncultured_Selenomonas	0.0055	0
d__Bacteria;p__Bacteroidota;c__Bacteroidia;o__Bacteroidales;f__Prevotellaceae;g__Prevotella;s__Prevotella_multisaccharivorax	0.00533	0
d__Bacteria;p__Firmicutes;c__Negativicutes;o__Veillonellales-Selenomonadales;f__Selenomonadaceae;g__uncultured;s__Veillonellaceae_bacterium	0.00483	0
d__Bacteria;p__Proteobacteria;c__Gammaproteobacteria;o__Pasteurellales;f__Pasteurellaceae;g__Histophilus;s__Histophilus_somni	0.00467	0
d__Bacteria;p__Bacteroidota;c__Bacteroidia;o__Bacteroidales;f__Prevotellaceae;g__Prevotella;s__unidentified	0.00417	0

d__Bacteria;p__Firmicutes;c__Bacilli;o__Erysipelotrichales;f__Erysipelatoclostridiaceae;g__Eggerthia;s__Eggerthia_catenaformis	0.00367	0
d__Bacteria;p__Firmicutes;c__Clostridia;o__Lachnospirales;f__Lachnospiraceae;g__Howardella;s__Catonella_sp.	0.00367	0
d__Bacteria;p__Fusobacteriota;c__Fusobacteriia;o__Fusobacteriales;f__Leptotrichiaceae;g__Leptotrichia;s__unidentified	0.00367	0
d__Bacteria;p__Bacteroidota;c__Bacteroidia;o__Flavobacteriales;f__Flavobacteriaceae;g__Flavobacterium;s__Cytophaga_sp.	0.003	0
d__Bacteria;p__Proteobacteria;c__Gammaproteobacteria;o__Enterobacterales;f__Enterobacteriaceae;g__Enterobacter;__	0.00283	0
d__Archaea;p__Thermoplasmatota;c__Thermoplasmata;o__uncultured;f__uncultured;g__uncultured;s__uncultured_archaeon	0.00267	0
d__Bacteria;p__Firmicutes;c__Negativicutes;o__Veillonellales-Selenomonadales;f__Veillonellaceae;g__Anaeroglobus;__	0.00267	0
d__Bacteria;p__Proteobacteria;c__Gammaproteobacteria;o__Enterobacterales;f__Enterobacteriaceae;g__Escherichia-Shigella;__	0.0025	0
d__Bacteria;p__Bacteroidota;c__Bacteroidia;o__Bacteroidales;f__Prevotellaceae;g__Prevotella;s__Prevotella_disiens	0.00233	0
d__Bacteria;p__Verrucomicrobiota;c__Verrucomicrobiae;o__Opitutales;f__Puniceicoccaceae;g__Verruc-01;__	0.00233	0
d__Bacteria;p__Actinobacteriota;c__Actinobacteria;o__Actinomycetales;f__Actinomycetaceae;g__Actinomyces;s__uncultured_bacterium	0.00217	0
d__Bacteria;p__Firmicutes;c__Bacilli;o__Bacillales;f__Planococcaceae;g__Rummeliibacillus;s__Rummeliibacillus_suwonensis	0.00217	0
d__Bacteria;p__Actinobacteriota;c__Coriobacteriia;o__Coriobacteriales;f__Eggerthellaceae;g__Slackia;s__Slackia_exigua	0.002	0
d__Bacteria;p__Bacteroidota;c__Bacteroidia;o__Bacteroidales;f__Tannerellaceae;g__Tannerella;s__uncultured_Tannerella	0.002	0
d__Bacteria;p__Firmicutes;c__Clostridia;o__Peptostreptococcales-Tissierellales;f__Anaerovoracaceae;g__Family_XIII_AD3011_group;s__uncultured_Eubacteriaceae	0.002	0
d__Bacteria;p__Bacteroidota;c__Bacteroidia;o__Bacteroidales;__;__;__	0.00183	0
d__Bacteria;p__Proteobacteria;c__Gammaproteobacteria;o__Burkholderiales;f__Comamonadaceae;g__Brachymonas;__	0.00183	0
d__Bacteria;p__Actinobacteriota;c__Coriobacteriia;o__Coriobacteriales;f__Eggerthellaceae;g__Cryptobacterium;s__Cryptobacterium_curtum	0.00167	0
d__Bacteria;p__Firmicutes;c__Clostridia;o__Lachnospirales;f__Lachnospiraceae;g__Lachnoanaerobaculum;s__Lachnoanaerobaculum_umeaense	0.00167	0
d__Bacteria;p__Actinobacteriota;c__Actinobacteria;o__Actinomycetales;f__Actinomycetaceae;g__Actinomyces;s__Schaalia_cardiffensis	0.0015	0
d__Bacteria;p__Actinobacteriota;c__Actinobacteria;o__Micrococcales;f__Dermatophilaceae;g__Piscicoccus;s__Piscicoccus_intestinalis	0.0015	0
d__Bacteria;p__Actinobacteriota;c__Actinobacteria;o__Actinomycetales;f__Actinomycetaceae;g__Trueperella;s__Trueperella_pyogenes	0.00133	0
d__Bacteria;p__Firmicutes;c__Clostridia;o__Peptostreptococcales-Tissierellales;f__Anaerovoracaceae;g__[Eubacterium]_brachy_group;s__Eubacterium_sp.	0.00133	0
d__Bacteria;p__Firmicutes;c__Clostridia;o__Peptostreptococcales-Tissierellales;f__Anaerovoracaceae;g__[Eubacterium]_nodatum_group;s__uncultured_bacterium	0.00133	0
d__Bacteria;p__Bacteroidota;c__Bacteroidia;o__Bacteroidales;f__Bacteroidaceae;g__Bacteroides;__	0.00117	0
d__Bacteria;p__Firmicutes;c__Clostridia;o__Monoglobales;f__Monoglobaceae;g__Monoglobus;__	0.00117	0
d__Bacteria;p__Proteobacteria;c__Gammaproteobacteria;o__Burkholderiales;f__Oxalobacteraceae;g__Herbaspirillum;__	0.001	0
d__Bacteria;p__Proteobacteria;c__Gammaproteobacteria;o__Coxiellales;f__Coxiellaceae;g__Coxiella;s__uncultured_proteobacterium	0.001	0
d__Archaea;p__Euryarchaeota;c__Methanobacteria;o__Methanobacteriales;f__Methanobacteriaceae;g__Methanobrevibacter;s__uncultured_rumen	0.00083	0
d__Bacteria;p__Actinobacteriota;c__Actinobacteria;o__Propionibacteriales;f__Propionibacteriaceae;g__Propionibacterium;__	0.00083	0
d__Bacteria;p__Firmicutes;c__Clostridia;o__Peptostreptococcales-Tissierellales;f__Anaerovoracaceae;g__Mogibacterium;__	0.00083	0
d__Bacteria;p__Proteobacteria;c__Gammaproteobacteria;o__Burkholderiales;f__Burkholderiaceae;g__Lautropia;s__unidentified	0.00083	0
d__Bacteria;p__Bacteroidota;c__Bacteroidia;o__Bacteroidales;f__Rikenellaceae;g__Rikenellaceae_RC9_gut_group;s__uncultured_bacterium	0.00067	0
d__Bacteria;p__Bacteroidota;c__Bacteroidia;o__Bacteroidales;f__Tannerellaceae;g__uncultured;__	0.0005	0
d__Bacteria;p__Bacteroidota;c__Bacteroidia;o__Flavobacteriales;f__Crocinitomicaceae;g__Wandonia;s__uncultured_bacterium	0.0005	0
d__Bacteria;p__Proteobacteria;c__Gammaproteobacteria;o__Oceanospirillales;f__Halomonadaceae;g__Halomonas;__	0.0005	0

d__Bacteria;p__Spirochaetota;c__Spirochaetia;o__Spirochaetales;f__Spirochaetaceae;g__Treponema;s__Treponema_pectinovorum	0.0005	0
d__Bacteria;p__Actinobacteriota;c__Actinobacteria;o__Micrococcales;f__Micrococcaceae;g__Rothia;s__Rothia_dentocariosa	0.00033	0
d__Bacteria;p__Bacteroidota;c__Bacteroidia;o__Bacteroidales;f__Prevotellaceae;g__Prevotella;s__Prevotella_amnii	0.00033	0
d__Bacteria;p__Firmicutes;c__Bacilli;o__Lactobacillales;f__Lactobacillaceae;g__Lactobacillus;s__Lactobacillus_rhamnosus	0.00033	0
d__Bacteria;p__Firmicutes;c__Clostridia;o__Oscillospirales;f__Ruminococcaceae;g__Caproiciproducens;s__bacterium_MS4	0.00033	0
d__Bacteria;p__Firmicutes;c__Clostridia;o__Proteinivoracales;f__uncultured;g__uncultured;s__uncultured_Anaerobranca	0.00033	0
d__Bacteria;p__Fusobacteriota;c__Fusobacteriia;o__Fusobacteriales;f__Leptotrichiaceae;__;__	0.00033	0
d__Bacteria;p__Firmicutes;c__Bacilli;o__RF39;f__RF39;g__RF39;s__Firmicutes_oral	0.00017	0
d__Bacteria;p__Proteobacteria;c__Gammaproteobacteria;o__Burkholderiales;f__Comamonadaceae;g__Delftia;__	0.00017	0
d__Bacteria;p__Proteobacteria;c__Gammaproteobacteria;o__Oceanospirillales;f__Halomonadaceae;g__Marinospirillum;__	0.00017	0
d__Bacteria;p__Bacteroidota;c__Bacteroidia;o__Bacteroidales;f__Prevotellaceae;g__Alloprevotella;s__unidentified_eubacterium	0	0

Supplementary Table 2. Dynamics of the different genera according to toothpaste group, after 6 weeks of use of the respective toothpastes.

**CPC/cym**		**MFP**	
**Increase**	**Decrease**	**Increase**	**Decrease**
Corynebacterium	*Atopobium*	*Actinotalea*	*Phocaeicola*
Alkaliflexus	*Phocaeicola*	*Bacteroides*	F0058
Parabacteroides	*Flexilinea*	*Dysgonomonas*	*Rikenellaceae* RC9 gut group
Acholeplasma	*Desulfobulbus*	*Alkaliflexus*	*Flexilinea*
Solobacterium	*Desulfovibrio*	*Paludibacter*	*Desulfobulbus*
Granulicatella	*Erysipelotrichaceae* UCG-006	*Parabacteroides*	*Mycoplasma*
Mycoplasma	*Lactobacillus*	*Mongoliitalea*	*Clostridia* vadinBB60 group
Clostridia UCG-014	*Lactococcus*	*Flavobacterium*	*Defluviitaleaceae* UCG-011
Defluviitaleaceae UCG-011	*Staphylococcus*	*Desulfovibrio*	genera of *Lachnospiraceae*
Ethanoligenens	*Pseudoramibacter*	*Erysipelothrix*	*Stomatobaculum*
Family XIII UCG-001	*Stomatobaculum*	*Solobacterium*	*Peptococcus*
Megasphaera	*Lachnospiraceae* uncultured	*Exiguobacterium*	[*Eubacterium*] *nodatum* group
JGI 0000069-P22	[*Eubacterium*] *nodatum* group	*Alkalibacterium*	[*Eubacterium*] *saphenum* group
genera of *Neisseriaceae*	*Peptostreptococcus*	*Enterococcus*	*Peptoanaerobacter*
Eikenella	*Serpentinicella*	*Lactococcus*	*Absconditabacteriales* (SR1)
	Selenomonadaceae uncultured	*Paenibacillus*	*Saccharimonadales*
	Dialister	*Anaerocolumna*	*Alkalimonas*
	Absconditabacteriales (SR1)	*Lachnoclostridium*	*Lautropia*
	Allorhizobium-Neorhizobium-Pararhizobium-Rhizobium group	*Ethanoligenens*	*Comamonas*
	Pseudomonas	*Fusibacter*	
	Xanthomonas	*Megasphaera*	
		JGI 0000069-P22	
		Brevundimonas	
		Ketogulonicigenium	
		Paracoccus	
		Candidatus Riegeria	
		Alishewanella	
		Brachymonas	
		genera of *Neisseriaceae*	
		Pseudomonas	
		IMCC26134	

Treatment groups: CPC/cym, toothpaste with cetylpyridinium chloride (CPC) and cymenol (cym), as main active ingredients; MFP, toothpaste with sodium monofluorophosphate (MFP).

Supplementary Table 3. Dynamics of the different species according to toothpaste group, after 6 weeks of use of the respective toothpastes.

CPC/cym		MFP	
**Increase**	**Decrease**	**Increase**	**Decrease**
Actinomyces genomosp.	*Actinomyces massiliensis*	*Actinomyces* genomosp.	*Phocaeicola abscessus*
Actinomyces unidentified	*Schaalia odontolytica*	*Actinomyces massiliensis*	F0058 uncultured bacterium
F0332 unidentified	*Phocaeicola abscessus*	*Actinomyces* unidentified	*Porphyromonas* spp.
Corynebacterium matruchotii	*Alloprevotella* spp.	*Actinotalea* spp.	*Alloprevotella tannerae*
Dysgonomonas uncultured bacterium	uncultured *Prevotella*	*Dysgonomonas* uncultured *Bacteroidetes*	*Prevotella buccae*
Alkaliflexus uncultured bacterium	*Prevotella denticola*	*Dysgonomonas* uncultured bacterium	*Prevotella dentalis*
Porphyromonas spp.	*Prevotella genomosp.*	*Alkaliflexus* uncultured bacterium	*Prevotella nanceiensis*
Prevotella nanceiensis	*Prevotella* unidentified *eubacterium*	*Paludibacter* uncultured bacterium	*Prevotella saccharolytica*
Prevotella sp.	*Rikenellaceae* RC9 gut group uncultured *eubacterium*	*Prevotella oralis*	*Prevotella shahii*
Parabacteroides sp.	uncultured *Capnocytophaga*	*Rikenellaceae* RC9 gut group uncultured eubacterium	*Prevotella* sp.
Tannerella spp.	*Anaerolineaceae bacterium*	*Parabacteroides* sp.	*Prevotella* unidentified *eubacterium*
Capnocytophaga spp.	*Desulfobulbus* spp.	*Capnocytophaga haemolytica*	*Porphyromonas*-like sp.
Capnocytophaga granulosa	*Streptococcus anginosus*	*Capnocytophaga leadbetteri*	*Tannerella* uncultured bacterium
Capnocytophaga haemolytica	*Paenibacillus* spp.	*Flavobacterium luticocti*	uncultured *Capnocytophaga*
Capnocytophaga ochracea	*Staphylococcus* spp.	*Anaerobacillus* uncultured bacterium	uncultured *Bergeyella*
Bacteroidia bacterium	*Pseudoramibacter* spp.	*Erysipelothrix* uncultured bacterium	*Campylobacter* spp.
Acholeplasma brassicae	*Stomatobaculum* uncultured bacterium	*Exiguobacterium* spp.	*Anaerolineaceae bacterium*
Anaerobacillus uncultured bacterium	[*Eubacterium*] *infirmum*	*Alkalibacterium* spp.	*Desulfobulbus* spp.
Granulicatella spp.	*Anaerovoracaceae* uncultured spp.	*Solobacterium moorei*	*Streptococcus anginosus*
Enterococcus italicus	*Peptostreptococcus* spp.	*Enterococcus* spp.	*Mycoplasma* spp.
Streptococcus spp.	*Centipeda* uncultured bacterium	*Enterococcus italicus*	*Metamycoplasma salivarium*
Streptococcus gordonii	*Selenomonas sputigena*	*Streptococcus gordonii*	*Clostridiales bacterium* (genus *Clostridia* UCG-014)
Streptococcus salivarius	*Dialister invisus*	*Streptococcus salivarius*	*Clostridiales bacterium* (genus *Clostridia* vadinBB60 group)
Mycoplasma spp.	*Fusobacterium nucleatum*	*Paenibacillus* spp.	*Lachnospiraceae bacterium*
Metamycoplasma salivarium	uncultured *Leptotrichia*	*Clostridia* UCG-014 spp.	*Johnsonella* uncultured bacterium
Clostridia UCG-014 spp.	Absconditabacteriales (SR1) bacterium	*Anaerocolumna* uncultured bacterium	*Stomatobaculum* uncultured bacterium
Lachnospiraceae bacterium	*Candidatus* Saccharimonas uncultured bacterium	*Catonella* sp.	*Peptococcus* sp.
Catonella sp.	TM7x uncultured *Candidatus*	*Johnsonella ignava*	[*Eubacterium*] *infirmum*
Johnsonella ignava	*Allorhizobium-Neorhizobium-Pararhizobium-Rhizobium* spp.	*Clostridium* sp.	[*Eubacterium*] *nodatum*
Ethanoligenens uncultured bacterium	*Xanthomonas* spp.	*Ethanoligenens* uncultured bacterium	*Eubacterium saphenum*
Serpentinicella spp.	*Treponema maltophilum*	*Fusibacter* spp.	*Peptoanaerobacter stomatis*
Selenomonas spp.		*Selenomonas artemidis*	*Schwartzia* sp.
Selenomonas unidentified		*Fusobacterium periodonticum*	*Selenomonas sputigena*
Anaeroglobus geminatus		*Leptotrichia hofstadii*	*Veillonellaceae bacterium*
Veillonella parvula		JGI_0000069-P22 spp.	*Absconditabacteriales* (SR1) bacterium
Fusobacterium periodonticum		*Gracilibacteria bacterium*	*Lautropia* spp.
Leptotrichia sp.		*Brevundimonas* spp.	*Lautropia* uncultured bacterium

Leptotrichia uncultured bacterium<br>JGI_0000069-P22 spp.<br>*Gracilibacteria bacterium*<br>*Saccharimonadaceae* spp.<br>TM7x uncultured bacterium<br>*Lautropia* uncultured bacterium<br>*Ottowia* sp.<br>*Eikenella* uncultured bacterium<br>*Neisseria bacilliformis*<br>*Aggregatibacter* uncultured bacterium<br>*Treponema* spp.<br>*Treponema* genomosp.		*Ketogulonicigenium vulgare*<br>*Paracoccus* spp.<br>*Candidatus* Riegeria uncultured bacterium<br>*Alishewanella* spp.<br>*Brachymonas* unidentified<br>*Neisseria oralis*<br>*Aggregatibacter* spp.<br>*Treponema* uncultured bacterium<br>IMCC26134 spp.	*Ottowia* sp.<br>*Neisseria elongata*<br>*Aggregatibacter* uncultured bacterium<br>*Pseudomonas* spp.<br>*Treponema denticola*<br>*Treponema maltophilum*<br>*Treponema socranskii*<br>*Treponema* sp.<br>*Treponema* unidentified

Treatment groups: CPC/cym, toothpaste with cetylpyridinium chloride (CPC) and cymenol (cym), as main active ingredients; MFP, toothpaste with sodium monofluorophosphate (MFP).

.

Supplementary Figure 1. Rarefaction curves calculated for the observed amplifier sequence variants (ASVs) (A), Shannon diversity (B) and Faith's index (C).

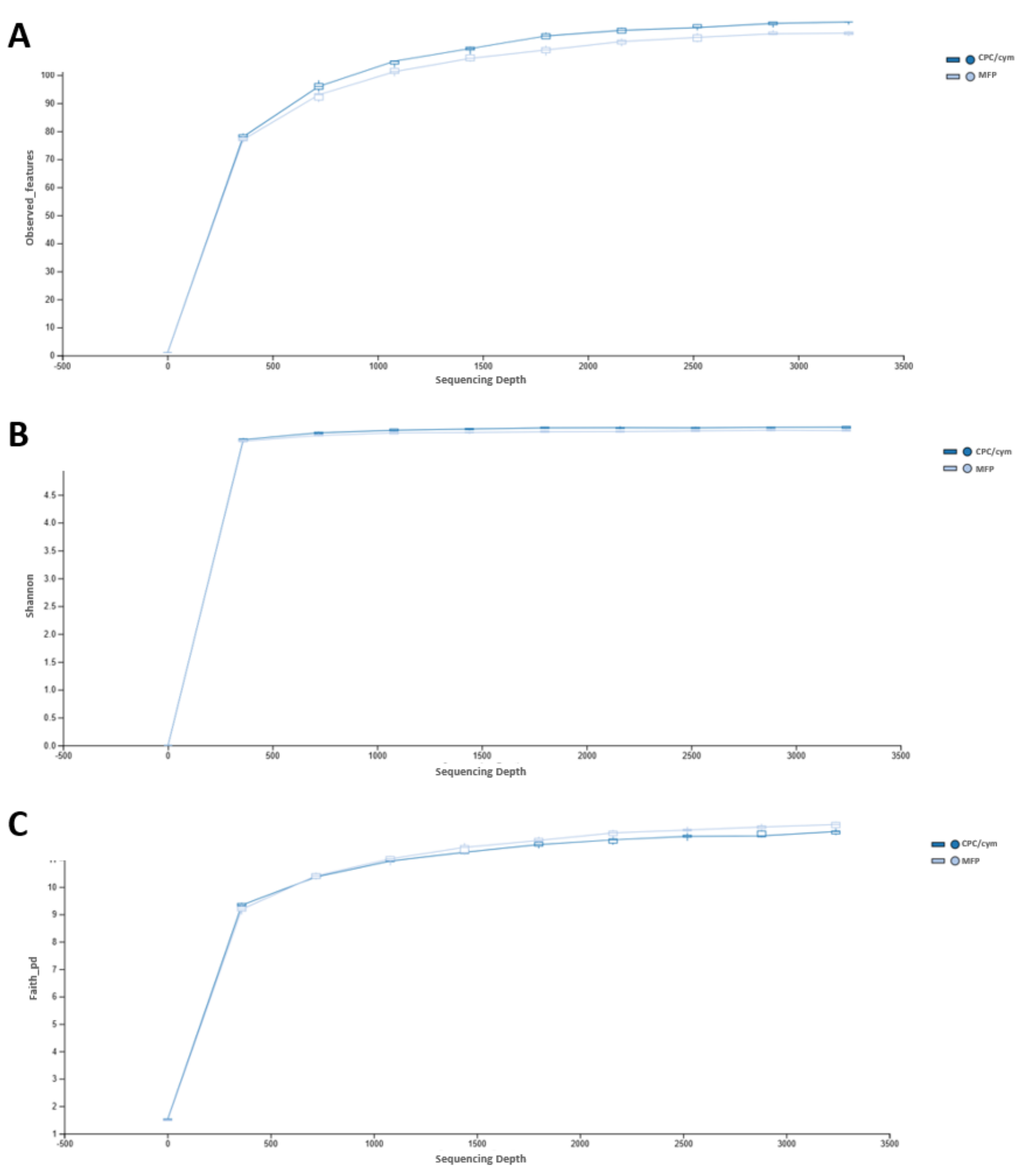

Treatment groups: CPC/cym, toothpaste with cetylpyridinium chloride (CPC) and cymenol (cym), as main active ingredients; MFP, toothpaste with sodium monofluorophosphate (MFP).

Supplementary Figure 2. Heat map grouping samples by genus abundance. Abundance data have been centered log-ratio transformed. Row labels add phylogenetic information. Colors represent the standardized abundances. Red indicates high abundance of the given genus, while blue indicates low abundance.

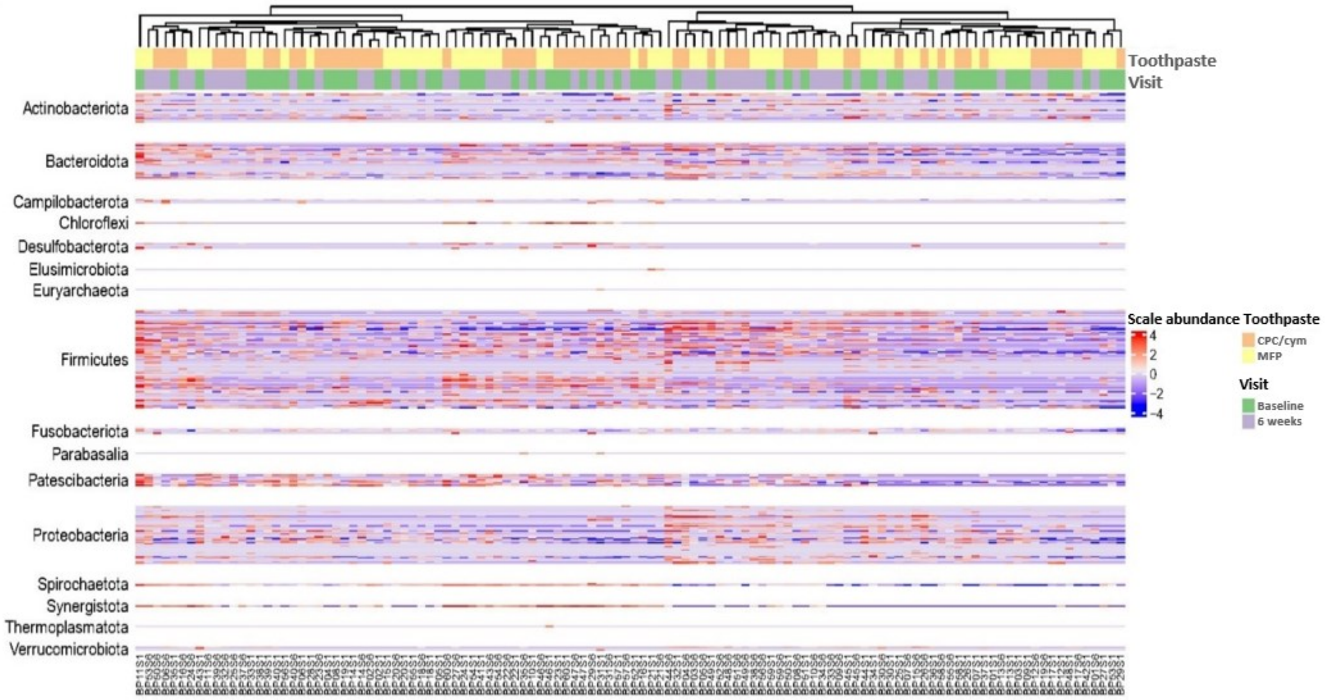

Treatment groups: CPC/cym, toothpaste with cetylpyridinium chloride (CPC) and cymenol (cym), as main active ingredients; MFP, toothpaste with sodium monofluorophosphate (MFP).
